# Formation and Investigation of Cell‐Derived Nanovesicles as Potential Therapeutics against Chronic Liver Disease

**DOI:** 10.1002/adhm.202300811

**Published:** 2023-09-17

**Authors:** Aymar Abel Ganguin, Ivo Skorup, Sebastian Streb, Alaa Othman, Paola Luciani

**Affiliations:** ^1^ Department of Chemistry Biochemistry and Pharmaceutical Sciences University of Bern Bern 3012 Switzerland; ^2^ Functional Genomics Center Zurich (FGCZ) University of Zurich/ETH Zurich Zurich 8057 Switzerland

**Keywords:** cell‐derived nanovesicles (cdNVs), corona, extracellular vesicles mimetics, liver fibrosis, LX‐2 cells

## Abstract

A new therapeutic approach using cell‐derived nanovesicles (cdNVs) is offered here to overcome the lack of effective treatments for liver fibrosis, a reversible chronic liver disease. To achieve this goal the formation and purification of cdNVs from untreated, quiescent‐like, or activated LX‐2 cells, an immortalized human hepatic stellate cell (HSC) line with key features of transdifferentiated HSCs are established. Analysis of the genotype and phenotype of naïve and transdifferentiated LX‐2 cells activated through transforming growth factor beta 1, following treatment with cdNVs, reveals a concentration‐dependent fibrosis regression. The beneficial fibrosis‐resolving effects of cdNVs are linked to their biomolecular corona. Liposomes generated using lipids extracted from cdNVs exhibit a reduced antifibrotic response in perpetuated LX‐2 cells and show a reduced cellular uptake. However, incubation with soluble factors collected during purification results in a new corona, thereby restoring fibrosis regression activity. Overall, cdNVs display encouraging therapeutic properties, making them a promising candidate for the development of liver fibrosis resolving therapeutics.

## Graphical abstract

Cell‐derived nanovesicles (cdNVs) have gained increasing attention as therapeutic biomaterial. cdNVs from hepatic stellate cells, the main collagen‐producing cells during hepatic fibrogenesis, are here produced, characterized and tested on activated LX‐2 cells, resulting in a concentration‐dependent reduction of cell activation. The fibrosis‐resolving effects of cdNVs are linked to their surface functional corona, making them a next‐generation biomaterial for liver fibrosis resolution.

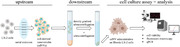

## 1 Introduction

The liver is involved in various vital functions, such as vitamin storage,^[^ ^1^ ^]^ clearance of damaged erythrocytes,^[^ ^2^ ^]^ elimination of xenobiotics,^[^ ^3^ ^]^ synthesis of plasma proteins,^[^ ^4^ ^]^ lipid management,^[^ ^5^ ^]^ and immune response.^[^ ^6^ ^]^ Injuries to this organ, caused, e.g., by viral infections, fatty diets, and/or alcohol abuse^[^ ^7^ ^]^ lead to the formation of extracellular matrix (ECM) proteins,^[^ ^8^ ^]^ which are an essential part of the wound‐healing response. However, if the cause of injury is not resolved, such as in chronic liver disease, fibrosis formation will be promoted leading, eventually, to switching from a reversible condition^[^ ^7^ ^]^ to irreversible liver cirrhosis, liver cancer, and liver failure, due to nonresolved excessive ECM protein production with consequent scar formation and loss of organ function.^[^ ^9^ ^]^ In such cases only a transplant can save the patient, making the liver the second most transplanted solid organ worldwide.^[^ ^10^ ^]^ The development of liver fibrosis involves different cell types, cytokines, and response cascades.^[^ ^7^, ^11^ ^]^ Hepatic stellate cells (HSCs) have been described to be the major driver of fibrosis progression in the liver.^[^ ^12^ ^]^ In physiologically healthy conditions, HSCs are in a quiescent (inactive) state in which they store vitamin A (retinol) in cytoplasmic lipid droplets.^[^ ^13^ ^]^ Upon activation, HSCs undergo a transdifferentiation into a highly proliferative myofibroblast‐like status.^[^ ^12^, ^14^ ^]^ In this activated state, HSCs proliferate, rapidly lose their ability to store lipid droplets, and start producing various ECM components, such as alpha‐smooth muscle actin (αSMA), fibronectin, type I and III collagens, as well as the profibrogenic cytokine transforming growth factor beta 1 (TGF‐β_1_) which further activates other HSCs.^[^ ^9^, ^12^ ^]^ The Global Burden of Diseases, Injuries, and Risk Factors Study 2017 (GBD 2017) estimated that 1.5 billion people were suffering from chronic liver diseases worldwide,^[^ ^15^ ^]^ 2 million annual deaths^[^ ^16^ ^]^ with the tendency increasing. Considering worldwide rising obesity and diabetes rates^[^ ^17^ ^]^ and the fact that only 11 high‐income countries are projected to be able to eliminate all hepatitis virus C infections by 2030,^[^ ^18^ ^]^ new effective therapies are needed to fight the already present increase in patients, in order to reduce the need of lifesaving transplantations.

Extracellular vesicles (EVs) have been extensively studied in the past two decades and show great promise for both diagnostic and therapeutic purposes in a variety of diseases.^[^ ^19^ ^]^ Because of their physicochemical properties, EVs can pass through biological barriers, elicit a low immune response, and thus have a longer circulation time in vivo.^[^ ^20^ ^]^ Proteins, peptides, nucleic acids, metabolites, and lipids can be carried by EVs and functionally transferred to target cells.^[^ ^20^ ^]^ Expression of cell‐specific epitopes on the EV surface enhanced targeted administration in vivo.^[^ ^21^, ^22^, ^23^ ^]^ As a result, EVs released from various cell sources, such as hepatocytes,^[^ ^24^ ^]^ endothelial cells,^[^ ^25^ ^]^ fibrocytes,^[^ ^24^ ^]^ macrophages,^[^ ^24^ ^]^ mesenchymal stem cells,^[^ ^24^, ^26^ ^]^ induced pluripotent stem cells^[^ ^11^, ^24^ ^]^ and HSCs^[^ ^24^, ^27^ ^]^ have been used to treat liver fibrosis. Depending on the cell state and harvesting point, various outcomes can be obtained. For example, EVs from palmitic acid (fibrotic)‐treated hepatocytes,^[^ ^28^ ^]^ as well as EVs from injured hepatocytes,^[^ ^24^, ^29^ ^]^ led to the activation and progression of HSCs. Consequently, it is crucial to understand the source of the EVs. Yet, the use of EVs is not without its challenges and limitations, which pose obstacles to their translation into clinical trials and their viability as a biotherapeutic option. These drawbacks include heterogeneity of the particle size, composition, and cargo, resulting in functional diversity, poor scalability, low yields, and a lack of regulations.^[^ ^20^, ^27^ ^]^ In order to address these challenges, engineered nanovesicles have emerged as a promising alternative.^[^ ^30^ ^]^ Inspired by the biological characteristics of natural EVs, engineered nanovesicles have demonstrated the ability to retain key biological and therapeutic features while addressing production, scalability, and standardization issues.^[^ ^31^, ^32^, ^33^, ^34^ ^]^

In a prior study conducted by our group, we demonstrated that EVs released from quiescent‐like LX‐2 cells^[^ ^35^ ^]^ induced an antifibrotic shift in LX‐2 cells.^[^ ^36^ ^]^ Inspired by these findings, we explored the potential of generating particles from quiescent LX‐2 cells as a therapeutic approach against chronic liver disease. Particles generated directly from cells have been referred to by various names in the literature, including artificial exosomes,^[^ ^31^ ^]^ exosome mimetic nanovesicles,^[^ ^37^ ^]^ cell‐engineered nanovesicles,^[^ ^38^ ^]^ and nanoghosts.^[^ ^39^ ^]^ In our study, we adopted the term “cell‐derived nanovesicles” (cdNVs) used in previously published work.^[^ ^40^ ^]^

To induce quiescence in LX‐2 cells, two established approaches were applied: treatment with retinol and palmitic acid, recognized as the gold standard for in vitro deactivation of HSCs,^[^ ^41^ ^]^ and treatment with the polyenylphosphatidylcholine‐rich (>75%) S80 liposomes, which our group demonstrated to exhibit antifibrotic effects on LX‐2 cells.^[^ ^42^ ^]^ To generate perpetuated, highly fibrogenic LX‐2 cells, TGF‐β_1_ was employed, as previously described.^[^ ^35^, ^43^ ^]^ Afterward, cdNVs were produced by subjecting differently pretreated LX‐2 cells (antifibrotic/fibrotic) to serial extrusion. cdNVs were then purified using a combination of density‐gradient ultracentrifugation steps, followed by standard ultracentrifugation. Characterization of the formed cdNVs included assessment of their size, particle concentration, protein and lipid content, and their biological activity on both naïve LX‐2 cells and TGF‐β_1_‐perpetuated LX‐2 cells.

## 2 Results

### 2.1 Formation and Purification of Cell‐Derived Nanovesicles

**Figure 1:**
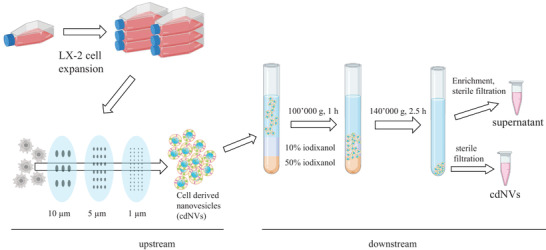
Formation and purification of cdNVs. Illustration of the formation of cell‐derived nanovesicles (cdNVs) from LX‐2 cells. Untreated or treated LX‐2 cells are harvested and serially extruded to form cdNVs, which are then purified with density gradient ultracentrifugation followed by ultracentrifugation. The supernatant (sn) was used as control in activity experiments.

The most suitable working protocol was established by forming cdNVs from untreated Dulbecco's Modified Eagle's Medium (DMEM) LX‐2 cells (3.0 × 10^7^ LX‐2 cells). Subsequently, the resulting particles were characterized in terms of size, yield, morphology, protein content, and zeta potential. As schematized in **Figure** **1**, LX‐2 cells were harvested from T175 flasks and cdNVs were formed by serial extrusion. Subsequently, the formed particles were purified by density gradient ultracentrifugation on a 50% w/v iodixanol and 10% w/v iodixanol cushion, followed by ultracentrifugation. The resulting cdNV sample contained 40% of the particle amount but less than 5% of the protein amount (Figure S1a, Supporting Information). The soluble factors, which are mostly peptides and proteins and not nucleic acids, ended up in the supernatant (sn). Hence, the sn contained more protein but less RNA compared to cdNVs (Figure S2, Supporting Information).

**Figure 2:**
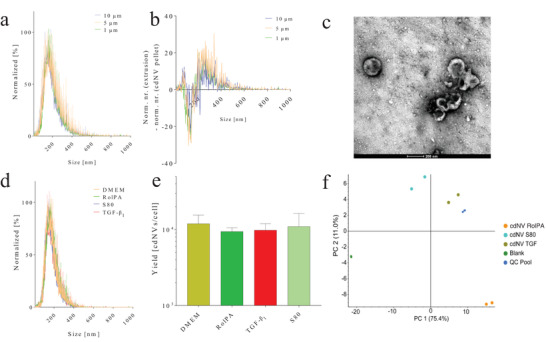
Characterization of cdNVs. a) Size distribution profile of cell‐derived nanovesicles (cdNVs) after formation through serial extrusion of untreated LX‐2 cells (mean ± SD (Standard deviation), *n* = 3). b) Subtraction of the particle number after purification (cdNV pellet) from the particle number after extrusion of untreated LX‐2 cells (mean ± SD, *n* = 3). c) TEM image of purified cdNVs derived from untreated LX‐2 cells. d) Size distribution profile of purified cdNVs derived from differently treated LX‐2 cells (mean ± SD, *n* = 3). e) cdNVs derived from differently treated LX‐2 cells yield normalized to the number of LX‐2 cells used for the formation (mean ± SD, *n* = 5). f) Principal component analysis (PCA) plot clustering the cdNV samples based on their similarity (*n* = 2).

The formed cdNVs showed a hydrodynamic diameter below 200 nm from the first extrusion on (**Figure** **2**a), whereas further purification lowered the number of particles bigger than 200 nm and increased the number of particles smaller than 200 nm (Figure 2b). The hydrodynamic diameter of cdNVs was 175 nm and was constant over cdNVs derived from all tested LX‐2 cells pretreatments (Figure S1b, Supporting Information). The surface charge of cdNVs was −38 mV at neutral pH and did not change with different LX‐2 conditions (Figure S1c, Supporting Information). Transmission electron microscopy (TEM) images confirmed the spherical morphology of the particles and the measured diameter (Figure 2c). Different pretreatments of LX‐2 cells prior to cdNV formation did not change the size distribution (Figure 2d) nor did they significantly change the yield/cell (Figure 2e) (ordinary one‐way ANOVA (Analysis of Variance) not significant (*p* value > 0.1)). To identify the various lipid species present in the cdNVs derived from DMEM‐, RolPA‐, TGF‐β_1_‐, and S80‐treated LX‐2 cells, a qualitative analysis of their lipid content was undertaken. In total, 255 different lipids from 13 different lipid classes were identified (Figure S3a, Supporting Information). The two replicates tested for each species were very similar, showing clear differences between treatments (Figure 2f). Pretreating HSCs with S80 clearly enriched the resulting cdNVs with dilinoleoylphosphatidylcholine (18:2; 36:4 in the phosphatidylcholine (PC) heatmap, Figure S3b, Supporting Information), as expected from our previous lipidomics analysis on LX‐2 cells treated with polyenylphosphatidylcholines. Supplementation of S80 to parent LX‐2 resulted in cdNV_S80 with a distinct composition of PC and ether phosphatidylcholine (PC O), phosphatidylethanolamine (PE) and ether phosphatidylethanolamine (PE O), phosphatidylinositol (PI) and lysophosphatidylcholine, while triglycerides (TG) lipids were enriched in cdNV_RolPA but not in cdNV_S80, nor cdNV_TGF (Figure S3b, Supporting Information).

### 2.2 Treatment of Naïve LX‐2 Cells with Cell‐Derived Nanovesicles Obtained from LX‐2 cells Treated with Either S80 or TGF‐β_1_

As previously shown by our group, S80 liposomes have a beneficial effect on the resolution of naïve LX‐2 cells,^[^ ^42^ ^]^ whereas TGF‐β_1_, a primary fibrotic cytokine in liver diseases,^[^ ^35^ ^]^ perpetuates LX‐2 cells by activation. Due to their opposite effect, S80 and TGF‐β_1_ were selected as pretreatment for LX‐2 cells. The formed cdNVs of fibrotic and quiescent (cdNV_TGF/cdNV_S80) origin were tested on naïve LX‐2 cells. First, the impact of freshly formed cdNVs on the metabolic activity of naïve LX‐2 cells was evaluated by resuspending the particles after purification in cell medium to a final concentration of 4.5 × 10^10^ cdNVs mL^−1^ and incubating them on the seeded naïve LX‐2 cells for 24 h. Treatment of naïve LX‐2 cells with 4.5 × 10^10^ cdNVs mL^−1^ led to a 25% increase in metabolic activity for both cdNV_S80 and cdNV_TGF, showing no toxicity (Figure S4a, Supporting Information). This was more than the result obtained by direct treatments with TGF‐β_1_ or S80 respectively, which both barely changed the cell metabolism.

**Figure 3:**
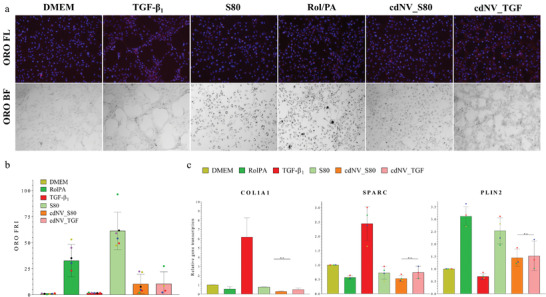
Effect of cdNVs on naïve LX‐2 cells. a) Representative 10x microscopy images of lipid droplets stained with Oil red O (ORO) in differently treated naïve LX‐2 cells. Lipid droplets in fluorescence (ORO FL) show in red (nuclei counterstained with DAPI (blue)) and in bright field (ORO BF) show in brown. b) Graph showing the ORO fluorescence normalized to the cell count in the DAPI field (FRI) and normalized to the ORO FRI of DMEM (mean ± SD, *n* ≥ 3) (*p*‐values from ordinary one‐way ANOVA with post post‐hoc Tukey test in Table S2, Supporting Information). c) Relative mRNA transcription in naïve LX‐2 cells of three fibrosis markers (COL1A1, SPARC, and PLIN2) normalized to GAPDH mRNA transcription and normalized to the DMEM condition after treatment with 4.5 × 10^10^ cell‐derived nanovesicles (cdNVs) mL^−1^ derived from S80 or TGF‐β_1_ treated LX‐2 cells and the corresponding controls (mean ± SD, *n* ≥ 3) (*p*‐values from ordinary one‐way ANOVA with post post‐hoc Tukey test in Tables S3–S5, Supporting Information).

Lipid droplets can be used to determine the phenotype of LX‐2 cells.^[^ ^36^, ^41^, ^42^ ^]^ They are present in a quiescent‐like state, such as after treatment with retinol supplemented with palmitic acid, the gold standard for in vitro deactivation of HSCs, and absent in naïve or activated LX‐2 cells.^[^ ^44^, ^45^ ^]^ Lipid droplets can be stained with Oil Red O (ORO) and appear as red spots in fluorescent images (ORO FL) or as brown spots in bright field images (ORO BF) under a fluorescent microscope. After treatment with 4.5 × 10^10^ cdNVs mL^−1^, we semi‐quantified lipid droplets in naïve LX‐2 cells through normalization of the area of droplets by the number of cells (stained with the fluorescent stain 4',6‐diamidino‐2‐phenylindole (DAPI)) (**Figure** **3**a). No difference was observed in the amount of lipid droplets upon treatment with cdNV_S80 and cdNV_TGF, suggesting an antifibrotic phenotype in LX‐2 cells after administration of both nanovesicle samples respectively (Figure 3b). The quiescent phenotype observed in naïve LX‐2 cells after cdNV application was not as pronounced as with the antifibrotic controls of direct treatment with 10 µm retinol and 300 µm palmitic acid or 5 mm S80 and was not significantly different from the vehicle control, where no lipid droplets were observed (Figure 3a,b). However, if the S80 liposomes were diluted to reach the same particle concentration as in the cdNV treatment, no difference in droplet presence was observed (ordinary one‐way ANOVA) (Figure S4b, Supporting Information).

After observing a transformation of the naïve LX‐2 phenotype, we measured the effect of cdNVs on the genotype of naïve LX‐2 cells by qPCR to confirm the quiescent‐like state. In order to determine the genotype, three different fibrotic mRNA markers were analyzed. The first gene of interest was COL1A1, which codes for the procollagene‐alpha‐type‐1 protein and which is upregulated in activated hepatic stellate cells as a wound healing response.^[^ ^35^, ^46^ ^]^ The second analyzed gene was SPARC, which codes for the secreted protein acidic and cysteine rich (SPARC) protein, which is upregulated in activated HSCs.^[^ ^36^, ^47^ ^]^ The function of SPARC is to enhance cell migration to the site of injury by binding and remodeling extracellular matrix proteins.^[^ ^48^ ^]^ The third studied gene was PLIN2, which codes for the adipose differentiation‐related protein (ADRP), enhanced in quiescent cells and responsible for the regulation and lipolysis of lipid droplets.^[^ ^41^, ^49^ ^]^ COL1A1 mRNA translation was decreased by 70% upon cdNV_S80 treatment and 50% upon cdNV_TGF treatment (Figure 3c). SPARC mRNA translation was decreased 50% upon cdNV_S80 and 30% upon cdNV_TGF treatments, respectively. PLIN2 mRNA translation increased 50% after both cdNV treatments. Hence, all three studied markers confirmed the antifibrotic potential of cdNVs. Even though treating naïve LX‐2 cells with cdNV_S80 showed a more enhanced trend toward quiescent transformation in naïve LX‐2 cells compared to cdNV_TGF, it is still unclear if the pretreatment of LX‐2 cells prior to cdNV formation influenced their outcome, as the difference was not significant (ordinary one‐way ANOVA). Also, if there was pretreatment specificity, a profibrotic response would have been expected from cdNV_TGF. If the S80 liposomes were diluted to the same particle concentration as in cdNVs prior treatment, the same amount of lipid droplets and PLIN2 mRNA expression was observed as upon cdNV administration (Figure S4c, Supporting Information). However, a minimal effect on COL1A1, like the observed decrease after control treatment with 1 mm HEPES, and no effect on SPARC was obtained. This indicates that the addition of specific lipids and the resulting increase in PLIN2 mRNA transcription alone were not enough to trigger an antifibrotic response and that therefore, other factors in the cdNVs impacted the fibrosis regression.

### 2.3 Treatment of TGF‐β_1_ Activated LX‐2 Cells with Cell‐Derived Nanovesicles Derived from LX‐2 Cells Treated with DMEM, RolPA, S80, or TGF‐β_1_

**Figure 4:**
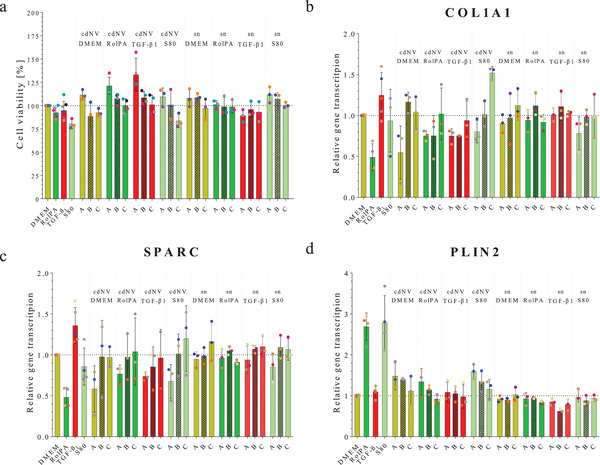
Effect of cdNVs and sn on TGF‐β1 activated LX‐2 cells. a) Cell viability of activated LX‐2 cells treated with three different cell‐derived nanovesicles (cdNVs) concentrations (*A* = 1.5 × 10^10^ cdNVs mL^−1^; *B* = 3.0 × 10^09^ cdNVs mL^−1^; *C* = 6.0 × 10^08^ cdNVs mL^−1^) derived from DMEM, RolPA, TGF‐β_1_, or S80 treated LX‐2 cells (mean ± SD, *n* ≥ 3) and treated with the sn control kept during cdNV production (*p*‐values from ordinary one‐way ANOVA with post post‐hoc Tukey test in Table S6, Supporting Information). b–d) Relative mRNA transcription in TGF‐β_1_ activated LX‐2 cells of three fibrosis markers (b, COL1A1, c) SPARC, and d) PLIN2) normalized to GAPDH mRNA transcription and normalized to the DMEM condition after treatment with three different cdNV concentrations (*A* = 1.5 × 10^10^ cdNVs mL^−1^; *B* = 3.0 × 10^09^ cdNVs mL^−1^; *C* = 6.0 × 10^08^ cdNVs mL^−1^) and three different sn protein concentrations (*A* = 20 µg mL^−1^; *B* = 4.0 µg mL^−1^, *C* = 0.8 µg mL^−1^) derived from DMEM, RolPA, TGF‐β_1_, or S80 treated LX‐2 cells (mean ± SD, *n* ≥ 3) (*p*‐values from ordinary one‐way ANOVA with post post‐hoc Tukey test in Tables S7–S9, Supporting Information).

HSCs release TGF‐β_1_ in fibrosis development which leads to a strong activation of neighboring HSCs.^[^ ^50^ ^]^ Therefore, the effect of cdNVs derived from untreated, RolPA, TGF‐β_1_, and S80 treated LX‐2 cells on TGF‐β_1_ activated LX‐2 cell viability was tested in a dose‐dependent manner with three different concentrations (*A* = 1.5 × 10^10^ cdNVs mL^−1^; *B* = 3.0 × 10^09^ cdNVs mL^−1^; *C* = 6.0 × 10^08^ cdNVs mL^−1^) and compared to the direct treatments with DMEM, RolPA, TGF‐β_1_, and S80, as well as the sn collected during cdNV production diluted to the same protein amount as in the cdNV samples (*A* = 20 µg mL^−1^; *B* = 4.0 µg mL^−1^, *C* = 0.8 µg mL^−1^) (Table S1, Supporting Information). The cells treated with the highest concentration of cdNVs showed a higher cell viability compared to those with the direct treatments, DMEM, RolPA, TGF‐β_1_, and S80 (111% vs 100% for cdNV_DMEM_A; 121% vs 92% for cdNV_RolPA_A; 133% vs 95% for cdNV_ TGF_A; 109% vs 80% for cdNV_S80_A), whereas only cdNV_ TGF_A was significantly higher from its direct treatment with TGF‐β_1_ (ordinary one‐way ANOVA, *p*‐value = 0.0006) (**Figure** **4**a). The induced cell proliferation was observed to be dose‐dependent in all cdNV samples, wherein lower concentrations led to a smaller increase and in the cases of cdNV_DMEM_B, cdNV_DMEM_C, and cdNV_S80_C, even a decrease in cell viability was observed (88%, 92%, and 84%, respectively). The difference in the increase in the cell viability increases of cdNV_RolPA and cdNV_TGF compared to cdNV_DMEM and cdNV_S80 were not significant and might be explained due to general biological variations obtained in cell culture experiments. Treatment with the sn samples barely had an influence on cell metabolism. The sn_DMEM and sn_S80 treatments increased the metabolism by 10% in a concentration‐dependent manner, whereas treatment with sn_RolPA had no effect at all and treatment with sn_TGF decreased the viability by 10% for all tested concentrations.

To further investigate the effect of cdNVs and sn derived from differently pretreated LX‐2 cells on TGF‐β_1_‐activated LX‐2 cells, we compared their influence on the translation of the COL1A1, SPARC, and PLIN2 genes. Proliferated LX‐2 cells showed a sixfold and 2.5‐fold increase in COL1A1 and SPARC mRNA levels respectively compared to DMEM‐treated naïve LX‐2 cells (Figure 3c). PLIN2 mRNA levels barely changed, which is consistent with a study performed by O'Mahony et al. in 2015, which showed that PLIN2 levels remained constant upon LX‐2 activation.^[^ ^51^ ^]^ The results show a dose‐dependent translational change of all tested fibrosis‐specific genes for cdNVs derived from all differently treated LX‐2 cells (Figure 4b,c,d).

The response is weakened upon dilution of cdNVs until the C dilution that drove no change in translation of the three investigated markers, except for the lowest dose of cdNV_S80, which led to further perpetuation of the TGF‐β_1_ activated LX‐2 cells by increasing COL1A1 mRNA translation 50% and increasing SPARC mRNA translation 20% (no significant difference to other cdNV_C samples by ordinary one‐way ANOVA). The most pronounced effect of all tested cdNV samples was observed in cdNV_DMEM_A; however, with a big standard deviation making it not significantly different from the other cdNV_A treatments. Among all cdNV samples, there was a smaller change in the translation of the three tested markers compared to direct treatment with RolPA, with the most pronounced difference observed in the transcription of the PLIN2 mRNA. On the other hand, compared to direct S80 treatment, treatments with 1.5 × 10^10^ cdNVs mL^−1^ (cdNV_A) led to a more pronounced decrease in SPARC and COL1A1 mRNA expression. When the same particle concentration was administered, the transcription of PLIN2 mRNA in perpetuated LX‐2 cells after S80 treatment did not show a greater enhancement compared to cdNV (Figure S5, Supporting Information), except if compared to cdNV_TGF which did not lead to an increase in PLIN2 mRNA transcription. No investigation into the translation of PLIN2 mRNA to ADRP protein was done. Nevertheless, these results suggest that the antifibrotic effect of cdNVs on LX‐2 cells is not, or only partially driven by ADRP and lipid droplet formation, which was discovered to be an important driver in fibrosis resolution of HSCs by Friedman et al. in 2010.^[^ ^41^ ^]^

Direct treatments with S80 and RolPA led to a 2.7‐fold increase in PLIN2 mRNA transcription. However, RolPA treatment resulted in a greater decrease in COL1A1 and SPARC mRNA levels compared to S80 treatment (51% vs 4% for COL1A1 mRNA and 52% vs 14% for SPARC mRNA). These findings indicate the involvement of different mechanisms in fibrosis resolution following RolPA or S80 treatments, respectively. While direct RolPA treatment induced the same relative change in mRNA translation of COL1A1, SPARC and PLIN2 in naïve and TGF‐β_1_ activated LX‐2 cells, direct S80 treatment had a smaller effect on TGF‐β_1_ activated LX‐2 cells (24–4% decrease for COL1A1 mRNA and 27–14% decrease for SPARC mRNA) (Figures 3c and 4b), suggesting that the antifibrotic response of S80 is lower in perpetuated LX‐2 cells compared to naïve LX‐2 cells. However, over 150 HSC markers^[^ ^52^ ^]^ are involved in fibrosis and therefore only looking at the full transcriptome and proteome could give a clear answer on the activity of S80 liposomes in liver fibrosis.

The sn samples showed a very small transcriptional change on the three tested markers in activated LX‐2 cells for the highest tested concentration, which tended to turn in a profibrotic direction upon dilution, proving the presence of active molecules in the sn. Only on PLIN2 mRNA expression no beneficial effect was observed after treatment with the highest tested sn concentration. This indicates that the addition of specific lipids through the cdNV membrane had an influence on the PLIN2 expression, whereas the decrease of COL1A1 and SPARC could have been triggered more by the nonlipidic fraction present in the sn and in the cdNV sample. To test if this hypothesis is correct, lipids were extracted from cdNVs using the Bligh and Dyer total lipid extraction protocol (see the Experimental Section) and their effect on LX‐2 cells was tested.

### 2.4 Treatment of TGF‐β_1_ Activated LX‐2 Cells with Liposomes Composed of Lipids Extracted from Cell‐Derived Nanovesicles Derived from DMEM, RolPA, S80 or TGF‐β_1_ Treated LX‐2 Cells

**Figure 5:**
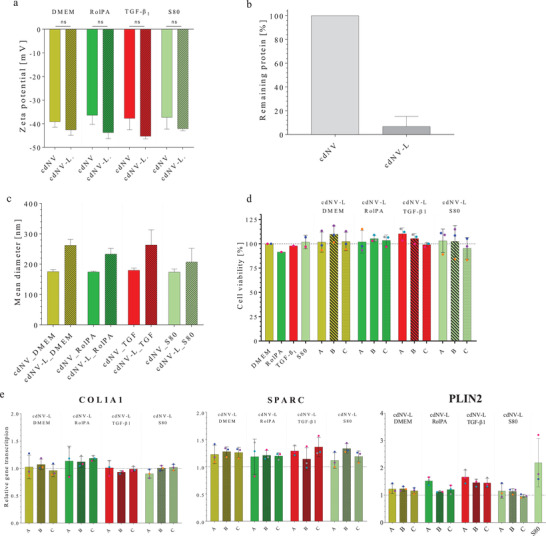
Characterization and activity of liposomes derived from cdNV extracted lipids. a) Zeta potential of cell‐derived nanovesicle (cdNV) liposomes (cdNV‐L) compared to cdNVs (mean ± SD, *n* = 3). b) Protein removal during the lipid extraction procedure (mean ± SD, *n* = 6). c) Hydrodynamic diameter comparison of cdNVs with cdNV‐L (mean ± SD, *n* ≤ 3). d) Cell viability of activated LX‐2 cells treated with three different cdNV‐L concentrations (*A* = 1.5 × 10^10^ cdNV‐L mL^−1^; *B* = 3.0 × 10^09^ cdNV‐L mL^−1^; *C* = 6.0 × 10^08^ cdNV‐L mL^−1^) (mean ± SD, *n* = 3). e) Relative mRNA transcription in TGF‐β_1_ activated LX‐2 cells of three fibrosis markers (COL1A1, SPARC, and PLIN2) normalized to GAPDH mRNA transcription and normalized to the DMEM condition after treatment with three cdNV extracted lipid liposome concentrations (*A* = 1.5 × 10^10^ cdNV‐L mL^−1^; *B* = 3.0 × 10^09^ cdNV‐L mL^−1^; *C* = 6.0 × 10^08^ cdNV‐L mL^−1^) (mean ± SD, *n* = 3).

After extracting lipids from cdNVs, lipid films were formed by evaporating the organic solvent under nitrogen gas. Liposomes were formed by simple rehydration of the lipid films in 10 mm HEPES for characterization experiments and in fetal bovine serum (FBS)‐free high glucose DMEM for activity studies and named cdNV‐liposomes (cdNV‐L). The formed cdNV‐L showed a more negative zeta potential, suggesting a charge‐masking effect on cdNVs through its membrane composition and surface corona (**Figure** **5**a). During lipid extraction, 93.5% ± 8.7% of all protein was removed indicating cdNV‐Ls that are almost purely composed of lipids (Figure 5b). As the rehydrated lipids were neither frozen/thawed nor extruded but simply sterile filtered, they displayed an increase in hydrodynamic diameter compared to cdNVs (Figure 5c), explaining the average polydispersity index (PDI) increased from 0.15 ± 0.03 for cdNVs to 0.26 ± 0.05 for the liposomes.

The effect of the cdNV‐L on TGF‐β_1_ activated LX‐2 cell viability was evaluated in a dose‐dependent manner using the same three concentrations *A*, *B*, and *C* (*A* = 1.5 × 10^10^ cdNV‐L mL^−1^; *B* = 3.0 × 10^09^ cdNV‐L mL^−1^; *C* = 6.0 × 10^08^ cdNV‐L mL^−1^) used above. The treated LX‐2 cells hardly showed a change in cell viability after treatment with all cdNV‐L tested conditions (Figure 5d), proving that the increase in cell metabolism after treatment with the highest cdNV concentration depended not only on the lipids but on the entire cdNV corona, cdNV content, and cdNV membrane composition. Furthermore, the potency of cdNV‐Ls was studied by investigating the expression of the same three qPCR targets (COL1A1, SPARC, and PLIN2) upon treatment of proliferated LX‐2 cells.

The tested cdNV‐L conditions did not influence the COL1A1 mRNA transcription, except for cdNV‐RolPA‐L which led to a slight increase (not significantly different to the DMEM control or other cdNV‐L treatments (ordinary one‐way ANOVA)) (Figure 5e). The SPARC mRNA translation increased by 20% after all cdNV‐L treatments. However, no concentration dependence was observed, suggesting that low amounts of extracted lipids led to an SPARC transcription increase which plateaued at an increase of roughly 20%. Only the PLIN2 mRNA transcription suggested that the treatment with cdNV‐L could result in a genotype with reduced fibrotic features. The cdNV‐Ls led to a concentration‐dependent increase in PLIN2 mRNA levels, similar to what was observed with cdNVs. The difference in PLIN2 mRNA increase observed between the different treatments (cdNV‐DMEM‐L/cdNV‐S80‐L ≈20% increase, cdNV‐RolPA‐L/cdNV‐TGF‐L ≈50% increase) might be explained by the LX‐2 cells used for the specific experiments, as not all treatments were performed on the same set of cells. Direct S80 treatment showed the highest PLIN2 mRNA transcription in the replicate which represents the cells used for cdNV‐RolPA‐L and cdNV‐TGF‐L treatments, and less high PLIN2 levels in the replicates which represent the cells used for cdNV‐DMEM‐L and cdNV‐S80‐L treatments.

Taken together, the results from treatments with extracted lipids from cdNV suggest that the lipid component of the vesicles only exerts an effect on lipid droplet formation, as proven by the increase in PLIN2 mRNA. We cannot exclude that the increase in PLIN2 mRNA upon cdNV‐L treatment was not enough to raise the ADRP to a level where it induces, according to a mechanism still to be understood, a decrease in ECM as described by Friedman et al. in 2010.^[^ ^41^ ^]^ Hence, the membrane structure and nonlipid membrane components, such as membrane proteins, along with potentially the cdNV content, play a significant role in modulating the transdifferentiation of HSCs induced by cdNVs. Moreover, the cdNV may offer a membrane surface for soluble factors and hydrophobic molecules. Together, they form a corona, which is involved in functional changes of recipient cells, as described in previous studies.^[^ ^53^, ^54^ ^]^

### 2.5 Treatment of TGF‐β_1_ Activated LX‐2 Cells with Proteoliposomes (PL) Formed through Reconstitution of Soluble Factors on the cdNV‐Liposome Surface

To address the effect of the corona on the activity of cdNVs, we reconstituted the soluble factors of the sn on cdNV‐Ls by incubation for 1 h at 37 °C in FBS‐free DMEM medium. The sn and cdNV‐L were derived from DMEM treated LX‐2 cells and the formed particles were termed PL. The sn and cdNV‐L concentrations were chosen to form PLs containing the same protein amount per particle as found on cdNVs (Table S1, Supporting Information). Also tested were proteoliposomes with 200 µg mL^−1^ of protein on 1.5 × 10^10^ cdNV‐L mL^−1^ termed PL_200. A concentration of 200 µg mL^−1^ protein was used, as an increase of the protein amount in sn led to an increased fibrosis‐resolving activity, even though the linear correlation was not as high as for cdNVs (Figure S6a,b, Supporting Information).

**Figure 6:**
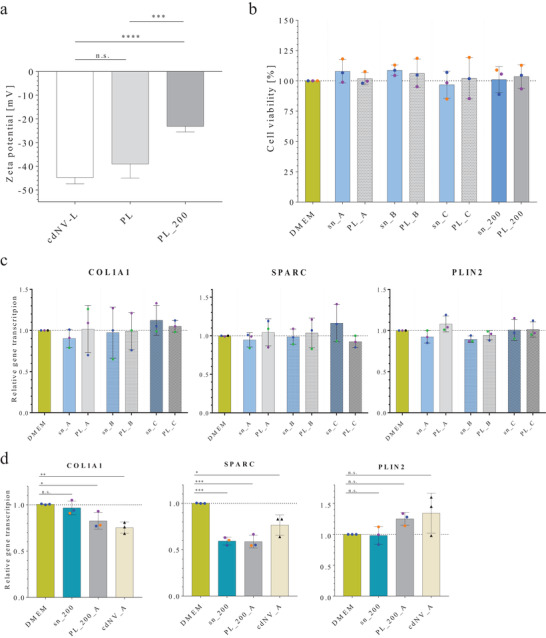
Characterization and activity of proteoliposomes. a) Zeta potential of cdNV‐L compared to PL and PL_200 (mean ± SD, *n* = 6) (*p*‐values from ordinary one‐way ANOVA with post post‐hoc Tukey test in Table S10, Supporting Information). b) Cell viability of activated LX‐2 cells treated with three different PL concentrations and the corresponding sn (*A* = 1.5 × 10^10^ cdNV‐L mL^−1^ + 20 µg mL^−1^; *B* = 3.0 × 10^09^ cdNV‐L mL^−1^ + 4.0 µg mL^−1^; *C* = 6.0 × 10^08^ cdNV‐L mL^−1^ + 0.8 µg mL^−1^) and PL_200 and the corresponding sn (*A* = 1.5 × 10^10^ cdNV‐L mL^−1^ + 200 µg mL^−1^) (mean ± SD, *n* = 3). c) Relative mRNA transcription in TGF‐β_1_ activated LX‐2 cells of three fibrosis markers (COL1A1, SPARC, and PLIN2) normalized to GAPDH mRNA transcription and normalized to the DMEM condition after treatment with the same three PL concentrations and the corresponding sn (mean ± SD, *n* = 3). d) Relative mRNA transcription in TGF‐β_1_ activated LX‐2 cells after treatment with PL_200 and the corresponding sn (mean ± SD, *n* = 3) (*p*‐values from ordinary one‐way ANOVA with post post‐hoc Tukey test in Tables S11 and S12, Supporting Information).

Physicochemical characterization of the PL and PL_200 samples by nanoparticle tracking analysis (NTA) did not reveal differences in the size distribution compared to cdNV‐Ls (Figure S7, Supporting Information). On the other hand, the zeta potential of PLs was less negative compared to cdNV‐L, even though not significant (Ordinary one‐way ANOVA), restoring the surface charge values measured for cdNVs (≈−40 mV) (**Figure** **6**a). Addition of more proteins further decreased the surface charge to −22 mV for PL_200 (Ordinary one‐way ANOVA, *p*‐value = 0.0001 vs cdNV‐L, *p*‐value = 0.0005 vs PL).

Cell viability studies showed that none of the tested protein and particle concentrations in the sn and PL samples had an influence on the cell metabolism (Figure 6b). Hence, the observed concentration‐dependent increase in cell viability after treatment with cdNVs (Figure 6a) must be influenced not just by a corona but by its composition and structure as well as other membrane components which are not present in the sn. Addition of sn or PL did not significantly change the expression of COL1A1, SPARC, and PLIN2 mRNA transcription in perpetuated LX‐2 cells compared to DMEM treatment (Figure 6c). However, the increase in SPARC mRNA transcription that was observed after cdNV‐L treatment was not observed in PL, hinting to a beneficial effect coming from the corona.

On the other hand, sn_200, as well as PL_200 did lead to an antifibrotic response of perpetuated LX‐2 cells upon treatment (Figure 6d). The COL1A1 mRNA transcription was decreased by 20% after PL_200 treatment, which was almost as much as observed after cdNV treatment. However, when treated with sn_200, no change in the COL1A1 mRNA transcription was observed attributing the observed activity to the PL_200 particles. However, SPARC mRNA transcription was decreased by 40% after PL_200 and sn_200 treatment, which was a more enhanced decrease than obtained after cdNV treatment (ordinary one‐way ANOVA, PL_200 vs cdNV_A *p*‐value = 0.0486; sn_200 vs cdNV_A *p*‐value = 0.0566). The PLIN2 mRNA did not increase after sn_200 treatment but almost 30% after PL_200 treatment. Hence, the antifibrotic potential in the sn can be enhanced with the presence of lipid particles due to the formation of a corona on the particles, leading to a synergistic effect of soluble factors and vesicles.

### 2.6 Cellular Uptake of Particles

**Figure 7:**
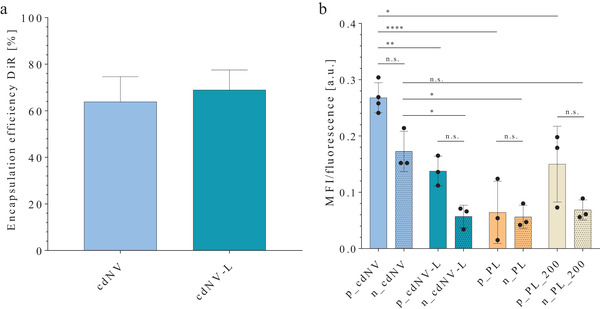
Cellular particle uptake. a) Encapsulation efficiency of the hydrophobic DiR‐dye in cell‐derived nanovesicles (cdNVs) and cdNV liposomes (cdNV‐L) (mean ± SD, *n* = 6). The encapsulation efficiency was calculated by division of the fluorescent intensity of the particles after removal of free drug by size exclusion chromatography (SEC) by the fluorescent intensity of the particles prior SEC. b) Cellular uptake of cdNVs, cdNV‐Ls, proteoliposomes with low protein amount (PL), and proteoliposomes with high protein amounts (PL_200) in naïve (n) and perpetuated (p) LX‐2 cells analyzed by flow cytometry. LX‐2 cells were seeded on 12‐well plates at 1.2 × 10^5^ cells per well. DiR‐labeled particles were added to the cells at 4.2 × 10^4^ particles per cell concentration and incubated at 37 °C for 24 h. The fluorescence intensity was measured using a flow cytometer to determine the uptake efficiency and the mean of all positive cells (MFI) was normalized to the fluorescence of the particles (MFI/fluorescence) (mean ± SD, *n* ≥ 3) (*p*‐values from ordinary one‐way ANOVA with post post‐hoc Tukey test in Table S13, Supporting Information).

Even though controversial, there is evidence of homing function in EVs due to their natural tropism.^[^ ^55^, ^56^, ^57^ ^]^ Artificial EVs have been previously described to preserve the targeting capacity of natural EVs.^[^ ^34^, ^58^ ^]^ To evaluate if cdNVs show an enhanced uptake in LX‐2 cells and to determine whether it is attributed to the membrane components and structure, a comparison was made between the uptake of cdNV, cdNV ‐L, PL, PL_200 by LX‐2 cells after 24 h. The particles were labeled with the hydrophobic nonexchangeable dye DiR and the uptake was measured via flow cytometry. The labeling efficiency for cdNVs and cdNV‐Ls was 65–70% (**Figure** **7**a). After 24 h cdNVs showed an almost threefold higher uptake in naïve LX‐2 cells compared to cdNV‐Ls (Figure 7b).

Addition of proteins on the cdNV‐L surface did not increase the particle uptake. Hence, the increased particle uptake observed in cdNVs must be due to a specific corona and other membrane components, such as membrane proteins. Glycosylation was shown to be involved in interactions of EVs with cell surfaces and represent the first contact points of cells with particles and might therefore also be a reason for the enhanced uptake.^[^ ^59^ ^]^ If the LX‐2 cells were perpetuated with TGF‐β_1_, an enhanced uptake was observed for all particles, even though it was not significantly different compared to naïve cells (Figure 7b). The reason for the observed enhancement of uptake in highly active LX‐2 cells compared to mildly activated naïve cells remains unknown. Since all particles demonstrated an increased uptake, it is possible that this may not be a specific mechanism and therefore could be due to a slight increase in membrane fluidity after TGF‐β_1_ treatment.^[^ ^42^ ^]^

Those observed uptake results correlate with the observed increase in PLIN2 mRNA transcription after treatment of perpetuated LX‐2 cells (Figure S8, Supporting Information). Even though the PLIN2 mRNA expression changes were not significant and not as enhanced as the cellular uptake changes, it shows that for lipid droplet formation lipids must be taken up and internalized.

## 3 Discussion

We described a method to generate and purify cell‐derived nanovesicles from untreated, quiescent, and fibrotic LX‐2 cells. The purification involving density‐gradient ultracentrifugation on an iodixanol cushion followed by ultracentrifugation removed over 95% of the proteins while preserving 40% of all generated particles. Still, a big variation in particle yield was observed which originated from the variability obtained after extrusion, showing an improvement potential at the formation step. Introduction of a prelysis such as through sonication or freeze/thawing could be beneficial to facilitate extrusion. Also, the addition of more extrusion cycles as well as extrusion through membranes with smaller pores should be considered, as it has already successfully been done by other groups.^[^ ^34^, ^40^, ^60^ ^]^ TEM microscopy as well as physicochemical characterization revealed a spherical shape of the particles with a nanoranged diameter and narrow size distribution. The cdNVs, derived from all tested conditions, induced a concentration‐dependent deactivation in naïve and TGF‐β_1_ activated LX‐2 cells, restoring parts of the lost lipid droplets, and decreasing COL1A1, as well as SPARC mRNA transcription.

Liposomes formed with extracted lipids from cdNV (cdNV‐L) revealed that the lipids were not the reason for the decrease in COL1A1 and SPARC mRNA expression, but that they are involved, through a mechanism yet to be understood, in the formation of new lipid droplets and the increase in PLIN2 mRNA transcription. Proteoliposomes were successfully formed by incubation of the supernatant, containing soluble factors, with cdNV‐L, as shown by the decrease in the particles’ zeta potential. Restoring the same number of proteins on cdNV‐L as present in cdNV did not restore any lost antifibrotic activity. However, increasing the protein amount further to 200 µg mL^−1^ recovered fibrosis‐resolving potential, proving a synergistic effect of particles and soluble factors. Further studies are needed to fully understand the role of lipids and soluble factors in the transdifferentiation of activated LX‐2 cells to a less active state, as the soluble factors in the sn and the soluble factors in/on the cdNV membrane are not necessarily the same.

Therefore, the purification process needs to be selected wisely, as it has an influence on the particle corona.^[^ ^53^ ^]^ Hence, as long as safety guidelines on Good Manufacturing Practice (GMP) by the Food and Drug Administration (FDA) and efficacy can be assured, the International Society of Extracellular Vesicles emphasized the importance of prioritizing functionality over purity in 2022.^[^ ^61^ ^]^ Additionally, a switch from harsh purification techniques such as ultracentrifugation to more gentle and scalable techniques, such as tangential flow filtration needs to progress, which has already started in the EV and EV‐mimetic field.^[^ ^34^, ^62^, ^63^ ^]^

Cellular uptake studies revealed that the cdNV membrane composition plays an integral role in the particle uptake. Forming cdNV‐Ls showed an almost 50% decreased uptake in perpetuated LX‐2 cells compared to cdNVs, reflecting the different response observed on the expression of PLIN2 mRNA. Addition of proteins on the cdNV‐L surface did not increase the particle uptake. The particles were preferentially internalized by highly active HSCs, providing an advantage as these cells are the primary target for fibrosis resolution.

## 4 Conclusion

Our results show the possibility to form, out of LX‐2 cells, nanoparticles with antifibrotic potential and enhanced uptake in fibrotic LX‐2 cells. However, to achieve a complete and thorough comprehension of the cdNVs’ mode of action, further investigations will be needed on the treated cells, nanoparticles, as well as sn, which should mostly reflect the LX‐2 cytosol.

In conclusion, we believe that our research helps the understanding of bioderived vesicles and contributes to the field of personalized medicine against liver diseases, which could become the next‐generation approach against liver fibrosis with the potential to positively impact millions of individuals globally and alleviate the rising demand for liver transplantation procedures.

## 5 Experimental Section

### Liposome Preparation

Liposomal formulations with soybean phospholipids with 75% phosphatidylcholine (S80; Lipoid, Germany) were prepared by the thin film hydration method. The calculated amount of lipids was weighed and dissolved with chloroform (Biosolve, Netherlands) in a round‐bottom glass flask. The organic solvent was evaporated under N_2_ stream and the lipid film was dried overnight in a desiccator. The lipid film was then rehydrated with 10 mm HEPES (4‐(2‐hydroxyethyl)‐1‐piperazineethanesulfonic acid, Carl Roth, Germany) and extruded ten times through a 0.2 µm polycarbonate membrane (Sterlitech, USA) with an LIPEX extruder (Evonik, Canada). The particles' size distribution (PDI) and hydrodynamic diameter were measured using a Litesizer 500 (Anton Paar, Austria).

### Cell Culture

LX‐2 cells were cultured in high glucose (4.5 g L^−1^) DMEM (Carl Roth, Germany) supplemented with 10 000 units L^−1^ of penicillin and streptomycin (P/S; Gibco, USA), 200 mm l‐glutamine (Sigma, USA), and 2% v/v of sterile filtered (0.2 µm, cellulose acetate membrane) FBS (Merck Millipore, USA) at 37 °C in a humidified atmosphere containing 5% CO_2_. The cells were treated with 10 µm retinol (Merck Millipore, USA) and 300 µm palmitic acid (RolPA; Merck Millipore, USA), 10 ng mL^−1^ TGF‐β_1_ (BioVendor, USA), or 5 mm S80 liposomes in serum‐free DMEM for 24 h. After treatment, the cells were detached with 2 mL Accutase (Merck Millipore, USA) per flask and incubated for 4 min at 37 °C.

### Cell‐Derived Nanovesicle Formation

(Pretreated) LX‐2 cells were washed with preheated (37 °C) phosphate‐buffered saline (PBS) (Carl Roth, Germany) and were detached as described above. The total cell amount was counted mixing a small aliquot 1:1 with trypan blue (Thermo Fisher Scientific, USA) and a 0.100 mm Neubauer chamber (Hecht Assistant, Germany). LX‐2 cells were washed by discarding the supernatant after centrifugation for 3 min at 500 g. The pellets were resuspended to reach a desired concentration between 1 × 10^6^ and 10 × 10^6^ LX‐2 mL^−1^ with fresh PBS. LX‐2 cells were extruded serially five times through a 10, 5, and 1 µm polycarbonate membrane (Sterlitech, USA) with an LIPEX extruder. The formed cdNVs were stored on ice till further procedure.

### Cell‐Derived Nanovesicle Purification

In a thick wall 10 mL PC centrifugal tube (Beckman Coulter, USA), 1 mL of cold (4 °C) 10% w/v iodixanol (Alere Technologies AS, Norway) was stacked on top of 1 mL cold 50% w/v iodixanol. Then, a cdNV solution (5 mL) was carefully pipetted on top of the iodixanol sandwich by holding the tube almost flat (75°) while pipetting. The tubes were centrifuged for 1 h at 4 °C and 100 000 g in an Optima L‐90K Ultracentrifuge (Beckman Coulter, USA), and 1 mL fractions were collected in precooled Axygen reaction tubes (Corning, USA). The cdNV fractions were mixed, diluted to 8 mL with PBS, and ultracentrifuged for 2.5 h at 4 °C with a speed of 140 000 g. The supernatant (sn) was enriched using a 10 000 MWCO (molecular weight cut off) Amicon Ultra‐4 Centrifugal Filter Unit (Merck Millipore, USA) and the pellet was resuspended in FBS‐free DMEM and sterilized by filtration through a 0.22 µm regenerated cellulose (RC) syringe filter (ThermoFisher, USA) after being vigorously vortexed and pipetted. Nanoparticle tracking analysis (NTA, ZetaView 8.05.05 SP2, Particle Metrix, Germany; Sensitivity: 70.0; Shutter: 100) was used to determine size distribution, yield, and zeta potential of cdNVs. The protein content was measured using a micro‐BCA protein assay kit (ThermoFisher, USA) following the manufacturer's instructions and RNA content was measured using a Nanodrop (ThermoFisher, USA).

### Lipid Analysis

Lipid extraction, lipidomics, and data analysis were adapted from Alshehry et al.^[^ ^64^ ^]^ Briefly, 100 µL of each sample were mixed with 1000 µL 1‐butanol/methanol (1:1, v/v) + 5 mm ammonium formate and vortexed. The solutions were then sonicated at 20 °C for 1 h. A centrifugation step was performed (16 000 × *g*, 10 min, 20 °C) before removal of 1000 µL supernatant which was dried down with N_2_ and resuspended in 200 µL isopropanol:MeOH (1:1) by shaking for 20 min (20 °C, 1000 rpm). The samples were centrifuged (16 000 × *g*, 10 min, 20 °C) again and 150 µL of the supernatant was transferred into a glass vial for LC‐MS. The QCpool consisted of 20 µL of each sample.

For the LC method a Waters Premier BEH C18 column (50 mm x 2.1 mm) was used at 60 °C together with a Thermo Vanquish Horizon Binary Pump. The method used a 2 µL sample with a flow rate of 1 mL for 7.5 min. The gradient went from 15% Buffer B to 99% Buffer B (Buffer A: 60% acetonitrile, 40% H_2_O, 5 mm NH_4_acetate; Buffer B: 90% isopropanol, 10% acetonitrile, 5 mm NH_4_acetate)

For the MS method a Thermo Q Exactive HF DDA top5 with two different resolutions (MS1 resolution 60`000; MS2 resolution 15`000) was used. The stepped normalized collision energy was 10, 20, and 30.

For the data analysis, the Thermo Compound Discoverer software 3.3 was used. Lipid identification was performed by matching to lipidblast and lipidmaps library. Afterward, the mirror plots (library vs data) were visually inspected to confirm the match and the presence of diagnostic fragments according to the lipids standard initiative guidelines. Only lipids which match the expected fragmentation spectra are included in the analysis.

### TEM

TEM was performed by the Microscopy Imaging Centre (MIC) of the University of Bern. Briefly, for imaging of negatively stained samples, 5 µL of the vesicle suspension was adsorbed on glow discharged and carbon coated 400 mesh copper grids (Plano, Germany) for 3 min. Grids were washed and stained with 2% uranyl acetate in water (Electron Microscopy Science, Switzerland) for 45 s. The excess fluid was removed by gently pushing them sideways to filter paper. Samples were then examined with a transmission electron microscope (Tecnai Spirit; FEI, USA) at 80 kV and equipped with a digital camera (Veleta; Olympus Life Science, Germany).

### General Design of Cell Assays

LX‐2 cells were seeded in cell culture plates in high glucose DMEM supplemented with 10 000 units L^−1^ of P/S, 200 mm L‐Glutamine, and 2% v/v FBS. After 18 h the medium was discarded and cells were washed with PBS. Either treatments were performed for 24 h directly on naïve LX‐2 cells in FBS‐free medium or the fibrotic LX‐2 cells were perpetuated with 10 ng mL^−1^ TGF‐β_1_ for 24 h prior to the treatments.^[^ ^65^ ^]^

### Quantitation of Viable Cell Number in Cytotoxicity Assays

10 000 LX‐2 cells were seeded in a 96‐well plate (Faust, Switzerland). The CCK‐8 assay (Merck Millipore, USA) was used to determine cell viability following the manufacturer's instructions. Briefly, the cells were washed after treatment with PBS and 90 µL FBS‐free medium + 10 µL CCK‐8 solution was added to each well. After further 2 h of incubation at 37 °C and 5% CO_2_, the absorbance at 450 nm was measured with a plate reader (Spark 10 M; Tecan, Switzerland). The cell viability was calculated with following equation

(1) $$\mathrm{Cell}\;\mathrm{viability}\;\left[\%\right]=\frac{{\mathrm{OD}}_{\mathrm{treatment}}}{{\mathrm{OD}}_{\mathrm{DMEM}}}\times 100$$

where “OD treatment” refers to the optical density of the LX‐2 cells treated with the specific treatment and “OD DMEM” refers to the optical density of LX‐2 cells treated with the vehicle (FBS‐free DMEM).

### Lipid Droplet Content Analysis

30 000 LX‐2 cells were seeded in a 48‐well plate (Faust, Switzerland) and the lipid droplet analysis was performed as described before with slight adjustments.^[^ ^42^ ^]^ Briefly, the treated cells were washed and fixed with 200 µL per well^−1^ Roti‐Histofix 4% (Carl Roth, Germany) for 10 min at room temperature (RT) and washed once with 1 mL per well deionized MilliQ water. LX‐2 cells were stained with 200 µL per well of a filtered (0.45 µm, polyvinylidene fluoreide (PVDF); Merck Millipore, USA) Oil Red O (ORO, 0.5% w/v; Merck Millipore, USA) solution in propylene glycol for 15 min at RT. The remaining ORO was removed and cells were washed twice with PBS. Nuclei were counterstained with 200 µL per well of a 4 µm DAPI solution (ThermoFisher, USA) in PBS for 5 min at RT. Afterward, cells were rinsed with PBS and acquired phase contrast (bright field, BF) as well as fluorescent images using an inverted Nikon Ti‐U microscope (Nikon Instruments, Japan) equipped with a Plan Fluor DL WD 15.2 10x, with a numerical aperture of 0.3 (Nikon instruments, Japan), DAPI filter (ex 360, em 460, 100 ms exposure), and Texas Red filter (ex 560, em 645, and 300 ms exposure).

The fluorescent binary area and the cell count were performed with the open‐source FIJI software using a script. The fluorescent binary area was then divided by the cell count to analyze how many lipid droplets [µm^2^] per cell are present.

### mRNA Transcription

60 000 LX‐2 cells were seeded in a 24‐well plate (Faust, Switzerland). After treatment, total RNA was isolated using TRIzol reagent (ThermoFisher, USA) following the manufacturer's instructions. Briefly, cells were lysed with TRIzol (ThermoFisher, USA) directly in the plate and transferred to Axygen reaction tubes (Corning, USA). A volume of chloroform equivalent to one‐fifth of the total TRIzol volume was added to the samples. The tube was vortexed vigorously for 10 s and was incubated at room temperature for 10 min before being centrifuged for 20 min at 4 °C and 16 000 g with a Hermle Z 366 K centrifuge (Faust, Switzerland). The upper phase was transferred to a new reaction tube and 1 µL glycogen (Merck Millipore, USA) as well as 1 volume of isopropanol (Biosolve, Netherlands) was added, prior to RNA precipitation on ice for 10 min. The RNA was pelleted by centrifugation for 10 min at 4 °C and 24 000 g. The supernatant was discarded and the pellet was washed with 1 mL 70% EtOH in diethyl pyrocarbonate(DEPC)‐treated (RNase‐free) MilliQ‐water (Carl Roth, Germany). The centrifugation was repeated twice and the final pellet was resuspended in DEPC‐treated MilliQ‐water. The RNA concentration was measured with a NanoDrop (ThermoFisher, USA).

The isolated RNA was reverse transcribed into cDNA. Briefly, 1000 ng of RNA were diluted in DEPC‐treated MilliQ‐water and were incubated for 5 min at 65 °C after addition of 3 µL of a 150 ng µL^−1^ random hexamer (Microsynth, Switzerland). After 10 min incubation at RT 13.5 µL^−1^ of premixed master mix was added (**Table** **1**) and the samples were incubated for another 10 min at RT, followed by a 1 h incubation at 50 °C and 20 min incubation at 75 °C. DEPC‐treated MilliQ‐water was added to reach a theoretical concentration of 8 ng/µL cDNA.

**Table 1:** Reverse transcription master mix composition.

1x master mix			
Reverse transcriptase buffer 10x	Agilent	5	µL
Dithiothreitol (DTT) 100 mm	Stratagene	5	µL
dNTPs 10 mm	ThermoFisher	2	µL
40 U µL^−1^ RiboLock RNase inhibitor	Fermentas	0.5	µL
Reverse transcriptase 200 rxn	Agilent	1	µL
Total volume		13.5	µL

The primers (**Table** **2**) were diluted in MilliQ to a primer pair solution of 2.5 µm of forward and reverse primer each. The remaining molecules (polymerase, nucleotides, buffer, fluorophore) for qPCR were in the Brilliant III Ultrafast SYBR Green qPCR master mix (MM; Agilent, USA). The cDNA samples were measured in triplicates or duplicates for each gene and cDNA dilution. A water control was added to see potential contaminations of the reagents. A pipetting robot (Qiagen, Germany) was used to pipette the samples (3 µL cDNA, 7.5 µL 2x MM, 3 µL primer mix, 1.5 µL water). After the samples were ready, they were transferred into the qPCR analyzer centrifuge (Qiagen, Germany) which performed 40 cycles of amplification at 95 and 60 °C, whereas the fluorescence was always measured at 60 °C (ex.: 470 nm, em.: 510 nm). After the 40 cycles a melting curve of each sample was measured. The data were analyzed using the Rotor Gene Q software and Excel. The qPCR data were analyzed using the delta CT method.^[^ ^66^ ^]^ Glyceraldehyde 3‐phosphate dehydrogenase (GAPDH) was used as a normalizer gene.

**Table 2:** Overview of the used primer pairs for qPCR.

Primer	Source	Sequence
fwd GAPDH	Prof. Dr. Mühlemann's research group, University of Bern	5'‐GAG TCA ACG GAT TTG GTC G‐3'
rev GAPDH	Prof. Dr. Mühlemann's research group, University of Ber	5′‐GAG GTC AAT GAA GGG GTC AT‐3′
fwd PLIN2	OriGene, HP205000	5′‐GAT GGC AGA GAA CGG TGT GAA G‐3′
rev PLIN2	OriGene, HP205000	5′‐CAG GCA TAG GTA TTG GCA ACT GC‐3′
fwd COL1A1	Thermo, Hs00521314_CE	5′‐GTT CAG TTT GGG TTG CTT GTC T‐3′
rev COL1A1	Thermo, Hs00521314_CE	5′‐CCT GCC CAT CGA TGT G‐3′
fwd SPARC	OriGene, NM_003118	5′‐TGC CTG ATG AGA CAG AGG TGG T‐3′
rev SPARC	OriGene, NM_003118	5′‐CTT CGG TTT CCT CTG CAC CAT C‐3′

### Lipid Extraction

The LX‐2 cells were pelleted and resuspended in 0.8 mL PBS before being transferred to a glass centrifuge tube with a Teflon cap. Ice‐cold 1:2 (v/v) chloroform/methanol (Biosolve, Netherlands) was added (3 mL) and the tube was vigorously vortexed. After a 5 min incubation on ice, 1 mL of chloroform and 1 mL of PBS were added to the tube, mixed well, and centrifuged for 2 min at 1000 g to induce phase separation. The lower phase was then collected with a Pasteur pipette and transferred into a round‐bottom glass flask. The organic solvent was evaporated under an N_2_ stream and the lipid film was dried overnight in a desiccator. The lipid film was rehydrated in FBS‐free DMEM medium and the formed liposomes (cdNV‐L) were sterilized by filtration through a 0.22 µm RC syringe filter. The cells were then treated with cdNV‐Ls as described above. The size distribution and hydrodynamic diameter of the particles were measured using a Litesizer 500 (Anton Paar, Austria).

### Protein Corona Reconstitution

PL with the same protein amount per particle as cdNVs or 10x more (PL_200) were formed by incubating cdNV‐Ls with enriched supernatant collected during cdNV purification in PBS for 1 h at 37 °C. The formed PLs were then diluted in FBS‐free DMEM medium to reach the desired particle concentrations. To determine the size distribution and zeta potential of PLs, NTA (ZetaView 8.05.05 SP2‐; Particle Metrix, Germany; Sensitivity: 70.0; Shutter: 100) was performed.

### Cellular Particle Uptake

In the uptake studies, 120 000 LX‐2 cells were seeded in a 12‐well plate (Faust, Switzerland). DiR‐labeled cdNVs and cdNV‐Ls were prepared by incubating 5.0 × 10^10^ particles with 10 µm DiR (ThermoFisher Scientific, USA) in 2% v/v EtOH for 1 h at 37 °C. For the activated cells, the seeded cells were treated with 10 ng mL^−1^ of TGF‐β_1_ for 24 h before particle administration. Control particles were incubated with 2% v/v EtOH for 1 h at 37 °C. The labeled particles were purified from free drug using a PD MiniTrap Sephadex G‐25 resin desalting column (Cytiva, USA) and normalized to their concentration using nanoparticle tracking analysis (Sensitivity: 70.0; Shutter: 100). PL and PL_200 were generated as described above and LX‐2 cells were treated for 24 h in FBS‐free DMEM medium with 4.2 × 10^4^ particles per cell. The cells were detached with 100 µL Accutase per well for 5 min at 37 °C and cell viability was determined with Pi staining. The fluorescent intensity of the cells was analyzed using FACS LSR II (BD, USA) and the mean of all APC‐Cy7 positive cells was normalized to the fluorescence of the particles that was measured with an Infinite 200 PRO MNano plate reader (Tecan, Switzerland) after the particles were diluted 1:1 in MeOH (excitation 750 nm, emission 780 nm). The cells were washed with FACS buffer (PBS + 2% v/v FBS) before analysis. The results were analyzed with FlowJo software (version 10.8; BD, USA) and at least 20 000 events were recorded per sample using FCS (370 V), SSC (240 V), Pi (410 V), and APC‐Cy7 (360 V) filters. The gating strategy used is described in Figure S1a in the Supporting Information.

### Statistical Analysis

Statistics were made with the software GraphPad Prism v. 7.05. If not stated otherwise, an ordinary one‐way ANOVA test was performed. If a significant difference was observed, a post‐hoc Tukey test comparing all groups was performed. All *p*‐values of post‐hoc Tukey tests can be found in the Supporting Information (* represents *p*‐value < 0.05; ** represents *p*‐value < 0.01; *** represents *p*‐value < 0.001; **** represents *p*‐value < 0.0001).

## Conflict of Interest

No private study sponsors had any involvement in the study design, data collection, or interpretation of data presented in this paper. P.L. declares the following competing interests: she has consulted and received research grants from Lipoid GmbH, Sanofi‐Aventis Deutschland, and DSM Nutritional Products Ltd. A.A.G. and I.S. declare no competing interests.

## Author Contributions

P.L. conceived and supervised the original project. A.A.G. devised and performed all the experiments and analyzed the data. I.S. supported in devising, performing, and analyzing the cellular uptake experiments. S.S. and A.O. performed and analyzed data from the lipidomics experiments. A.A.G. led paper writing. P.L. and I.S. reviewed and edited the paper.

## Supporting information

Supporting Information

## Data Availability

The data that support the findings of this study are available from the corresponding author upon reasonable request.
