# Supplementary material for: Formation and Investigation of Cell‐Derived Nanovesicles as Potential Therapeutics against Chronic Liver Disease
Source: Adv Healthc Mater. 2023 Sep 17;12(30):2300811. doi: 10.1002/adhm.202300811 (PMC11468924; doi:10.1002/adhm.202300811)
Supplement: Supplementary file 1 — Supporting Information [file ADHM-12-2300811-s001.pdf]

ADVANCED HEALTHCARE MATERIALS

Supporting Information

for *Adv. Healthcare Mater.*, DOI 10.1002/adhm.202300811

Formation and Investigation of Cell-Derived Nanovesicles as Potential Therapeutics against Chronic Liver Disease

*Aymar Abel Ganguin, Ivo Skorup, Sebastian Streb, Alaa Othman and Paola Luciani**

Supporting Information

Formation and investigation of cell-derived nanovesicles as potential therapeutics against chronic liver disease

Aymar Abel Ganguin[1], Ivo Skorup[1], Sebastian Streb[2], Alaa Othman[2], Prof. Paola Luciani[1,]*

[1] Department of Chemistry, Biochemistry and Pharmaceutical Sciences, University of Bern, 3012 Bern, Switzerland
[2] Functional Genomics Center Zurich (FGCZ), University of Zurich/ETH Zurich, 8057 Zurich, Switzerland

E-mail: paola.luciani@unibe.ch

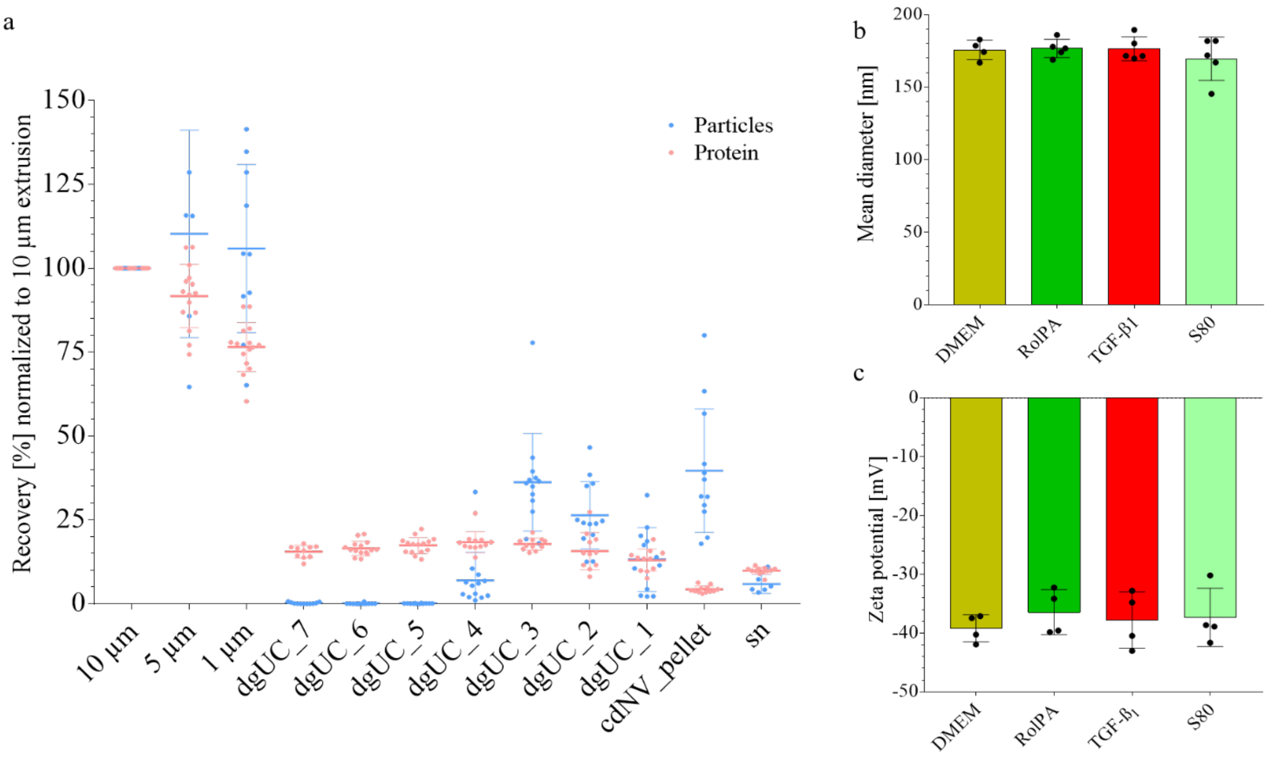

Figure S1. Purification and characterization of cdNVs.
a, Particle and protein recovery during purification of cdNVs normalized to values obtained after 10 µm extrusion (dgUC, density-gradient ultracentrifugation fractions) (mean ± SD, n ≤ 6). **b,** Mean diameter of purified cdNVs derived from differently treated LX-2 cells (mean ± SD, n ≤ 4). **c,** Zeta potential of purified cdNVs derived from differently treated LX-2 cells (mean ± SD, n = 4).

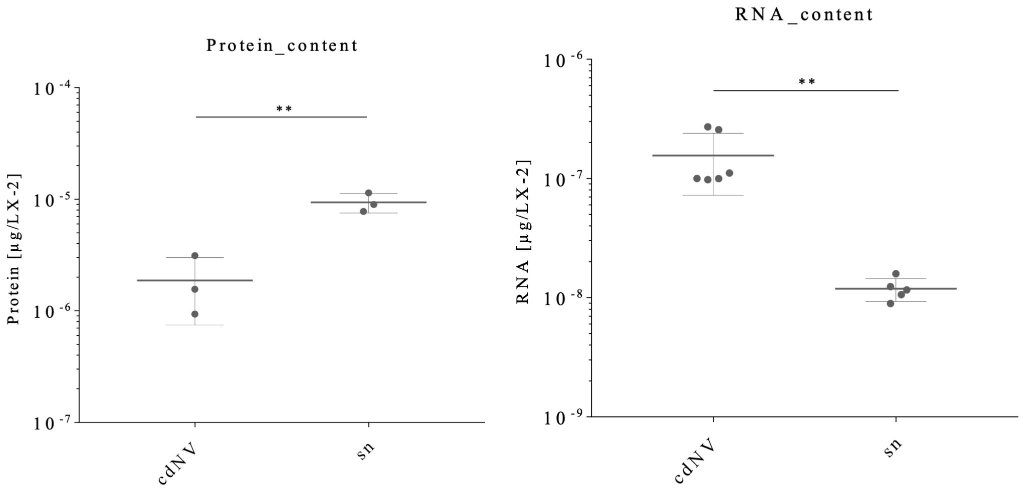

Figure S2. Characterization of protein and RNA content in cdNVs and sn sample.
The protein content, measured by a micro-BCA assay kit, and the RNA content, measured by nanodrop after RNA extraction from the cdNV and sn sample after purification. The starting cell amount was 5.0 x 10^6 LX-2-cells/mL and the used cells were untreated. Overall, there was much more protein in the sample compared to RNA. The sn contained ~5x more protein compared to the cdNV sample, however, ~15x less RNA (Protein: mean±SD, n=3, t-test; RNA: mean±SD, n≥5, t-test).

a

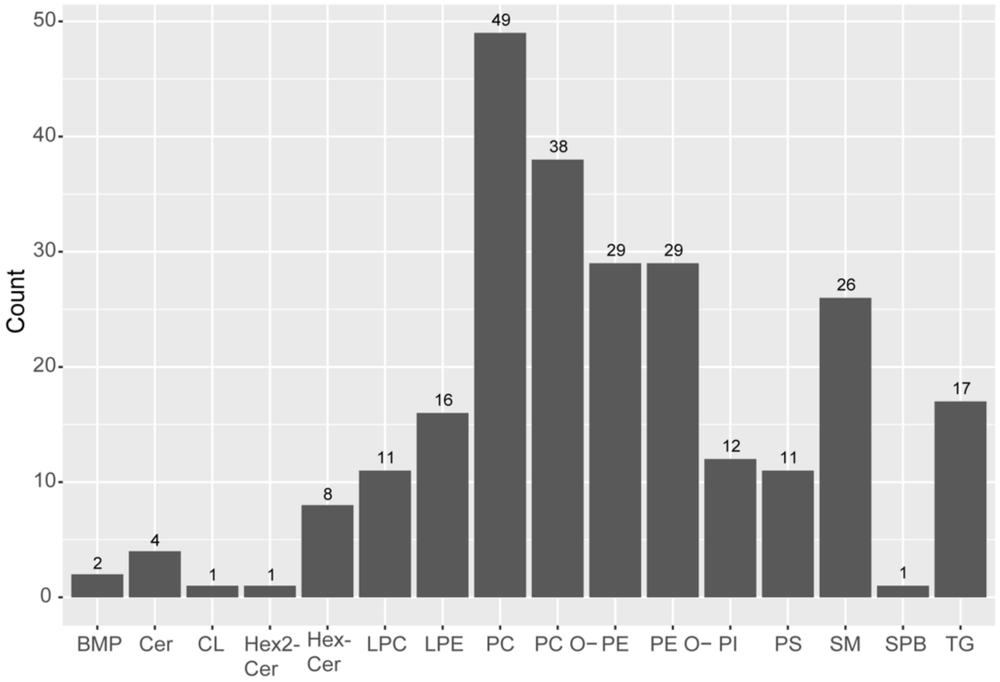
Count
50
40
30
20
10
0
2
4
1
1
8
11
16
49
38
29
29
12
11
26
1
17
BMP
Cer
CL
Hex2-Cer
Hex-Cer
LPC
LPE
PC
PC O−
PE
PE O−
PI
PS
SM
SPB
TG

b

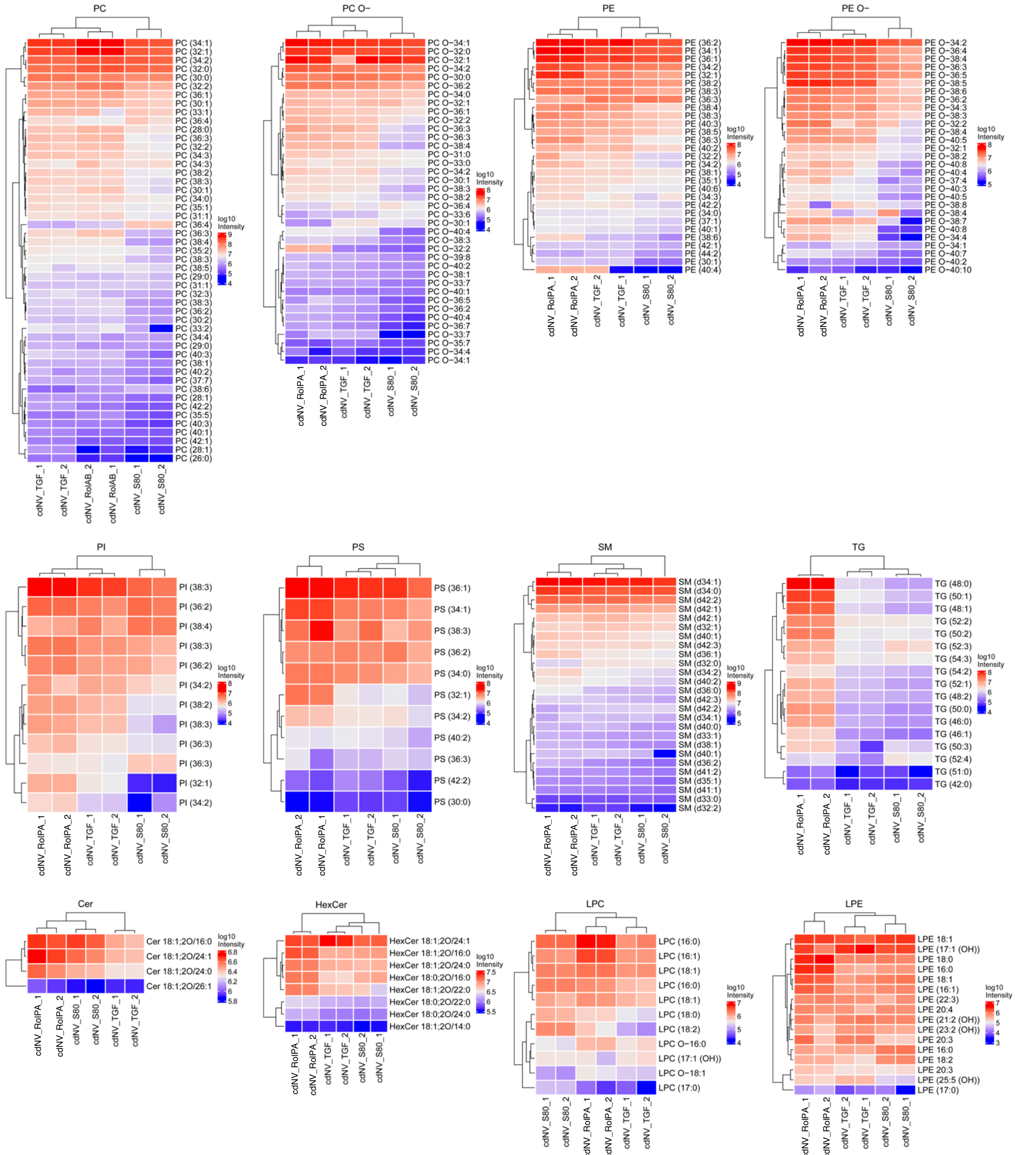
PC
PC O−
PE
PE O−
PI
PS
SM
TG
Cer
HexCer
LPC
LPE
log10 Intensity

Figure S3. Lipid characterization of cdNVs.
a, Lipid class overview and number of different species discovered per class. (Monoacylglycerophosphomonoradylglycerols (BMP), Ceramide (Cer), ceramide-sugar (HexCer), cardiolipin (CL), lysophosphatidylcholine (LPC), lysophosphatidylethanolamine (LPE), phosphatidylcholine (PC), phosphatidylethanolamine (PE), phosphatidylinositole (PI), phosphatidylserine (PS), sphingomyelins (SM), sphinganine (SPB), triglyceride (TG)). **b,** Heatmap of peak intensity in the chromatogram to compare the abundance of different lipid species between different cdNV samples. Ether phosphatidylcholine (PC O); ether phosphatidylethanolamine (PE O).

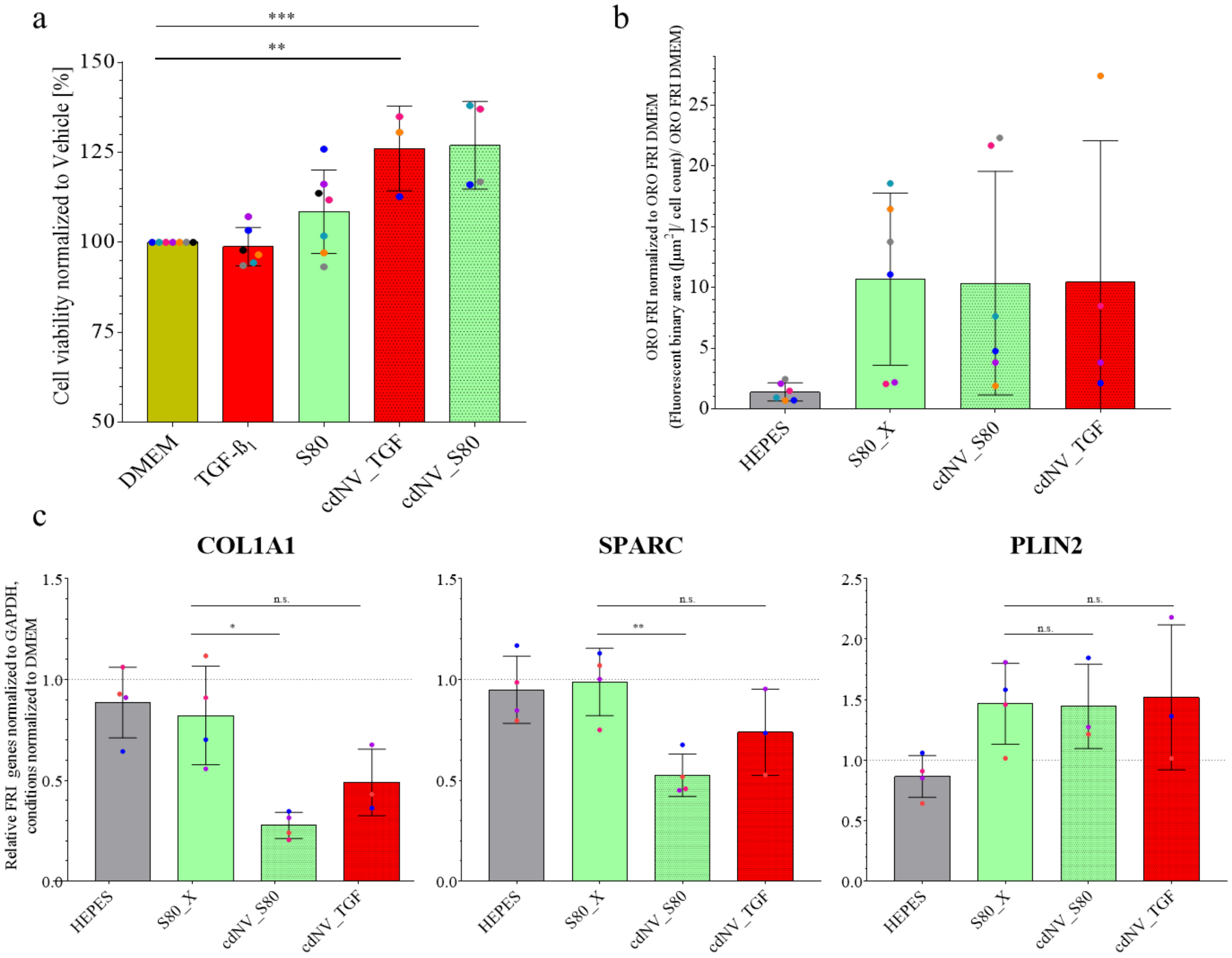

Figure S4. Effect of cdNVs on naïve LX-2 cells.
a, Cell viability of naïve LX-2 cells treated with 4.5 x 10^{10} cdNVs/mL derived from TGF-β1 or S80 treated LX-2 cells (mean ± SD, n ≤ 3) (p-values from ordinary one-way ANOVA with post post-hoc Tukey test in supplementary table SY5) **b,** ORO fluorescence normalized to the cell count in the DAPI field (FRI) and normalized to the ORO FRI of DMEM. Comparison of naïve LX-2 cells treated either with 4.5 x 10^{10} S80 liposomes/mL, or with 4.5 x 10^{10} cdNVs/mL (mean ± SD, n ≤ 4) (p-values from ordinary one-way ANOVA with post post-hoc Tukey test in supplementary table SY) **c,** Relative mRNA transcription in naïve LX-2 cells of three fibrosis markers (COL1A1, SPARC, PLIN2) normalized to GAPDH mRNA transcription and normalized to the DMEM condition after treatment with either 4.5 x 10^{10} cdNVs/mL or 4.5 x 10^{10} S80 liposomes/mL (mean ± SD, n ≤ 3) (p-values from ordinary one-way ANOVA with post post-hoc Tukey test in supplementary tables SY2/3/4)

Table S1. Protein amount of cdNV particles.

	cdNVs/mL	Protein concentration [µg/mL]	SD [µg/mL]	Protein [µg]	SD [µg]
A	1.5E+10	19.5	6.7	9.8	3.4
B	3.0E+09	3.9	1.3	2.0	0.7
C	6.0E+08	0.8	0.3	0.4	0.1

The amount of protein present in each of the three tested cdNV dosages (mean ± SD, n = 13). Total protein amount of cdNVs, obtained with a micro-BCA assay kit, was divided by the total particle amount of cdNVs, obtained through NTA, and multiplied by the particle amount of the respective dosages A, B, and C.

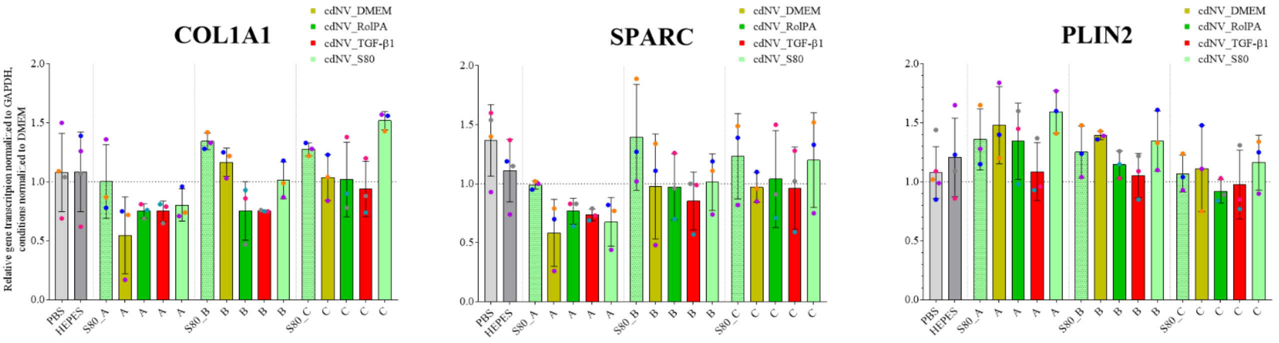

Figure S5. Comparison of cdNVs and S80 liposomes induced response in activated LX-2 cells. Relative mRNA transcription in TGF-β1 activated LX-2 cells of three fibrosis markers (COL1A1, SPARC, PLIN2) normalized to GAPDH mRNA transcription and normalized to the DMEM condition after treatment with three different cdNV and S80 liposome concentrations (A = 1.5 x 10^{10} particles/mL; B = 3.0 x 10^{09} particles/mL; C = 6.0 x 10^{08} particles/mL) (mean ± SD, n ≤ 3) (p-values from ordinary one-way ANOVA with post post-hoc Tukey test in supplementary tables SZ2/3/4) The anti-fibrotic response in activated LX-2 cells is stronger after cdNV treatment compared to S80 liposome treatment.

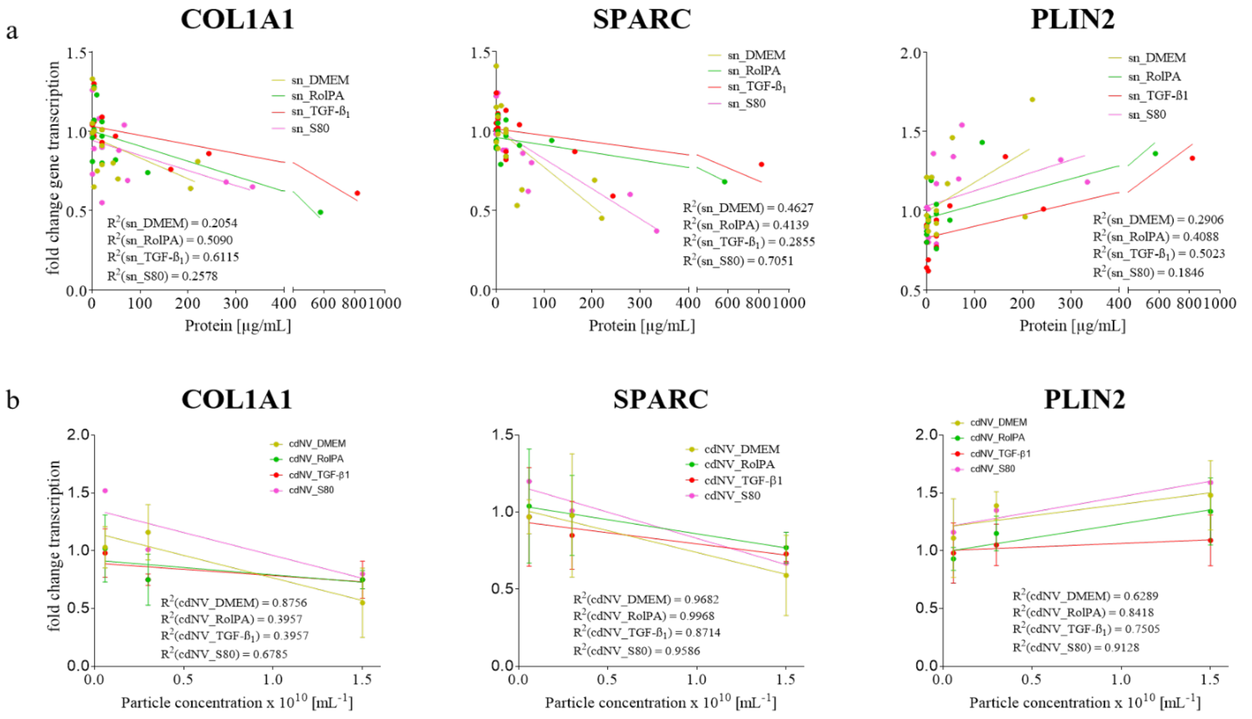

Figure S6. Correlation between mRNA expression and sn respectively cdNV concentration.
a, Linear correlation of relative mRNA transcription in TGF-β1 activated LX-2 cells of three fibrosis markers (COL1A1, SPARC, PLIN2) normalized to GAPDH mRNA transcription and normalized to the DMEM condition after treatment with sn containing increasing protein amounts (mean ± SD, n ≤ 3).
b, Linear correlation of relative mRNA transcription in TGF-β1 activated LX-2 cells of three fibrosis markers (COL1A1, SPARC, PLIN2) normalized to GAPDH mRNA transcription and normalized to the DMEM condition after treatment with three different cdNV concentrations (A = 1.5 x 10^{10} cdNVs/mL; B = 3.0 x 10^{09} cdNVs/mL; C = 6.0 x 10^{08} cdNVs/mL) (mean ± SD, n = 3).
Overall, cdNVs show a higher correlation compared to sn. As the mechanism of activity and active substances are unknown a linear correlation based on protein concentration alone might not be the best fit for the sn.

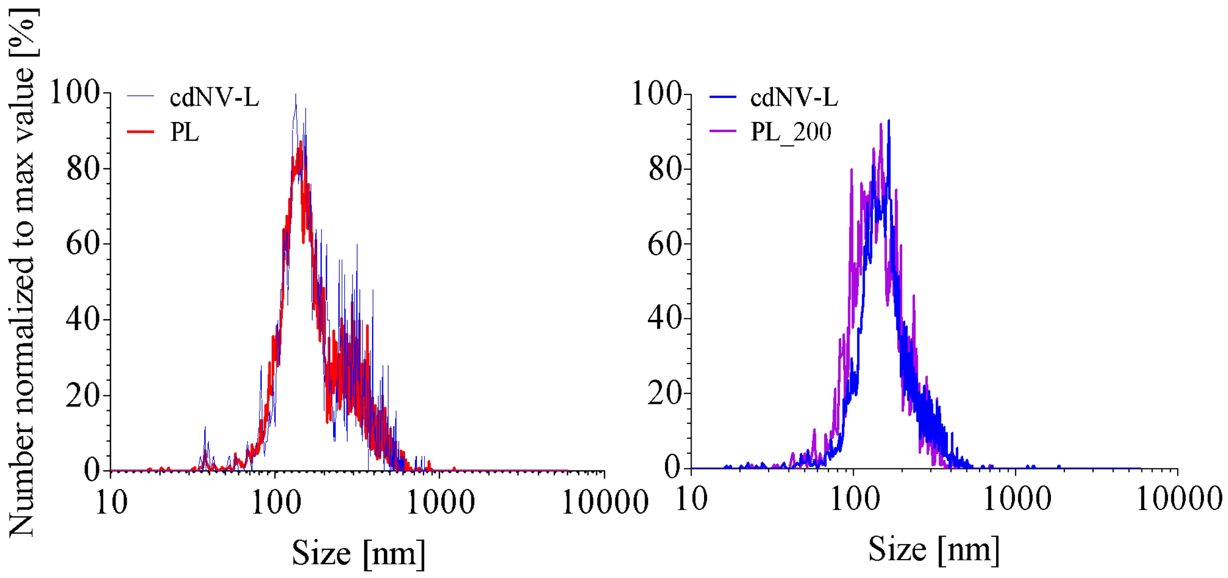

Figure S7. Characterization and activity of proteoliposomes.
Size distribution of cell-derived nanovesicle (cdNV) liposomes (cdNV-L) compared to proteoliposomes with low and high protein corona contents (PL and PL_200) (mean ± SD, n = 3).

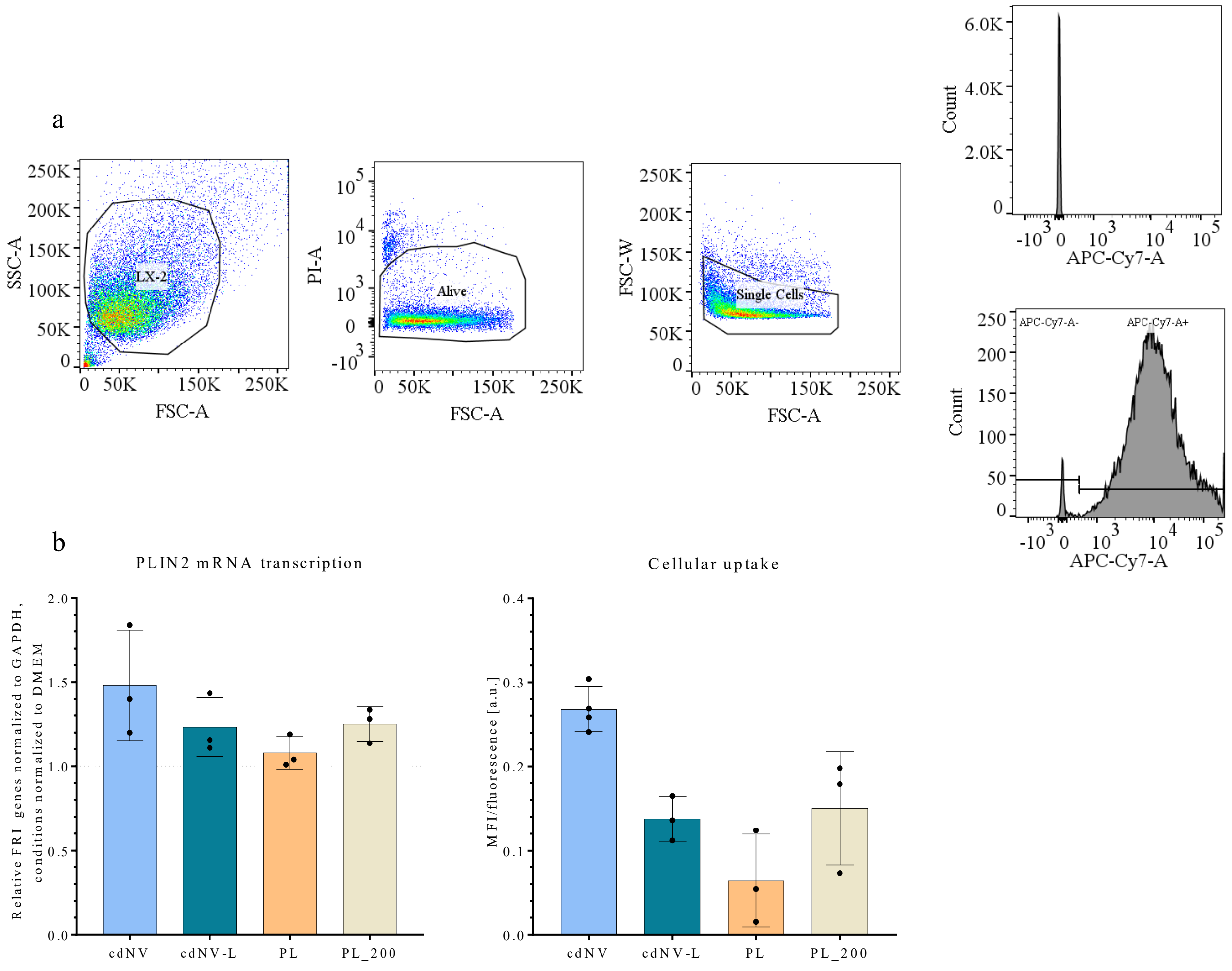

Figure S8. Cellular particle uptake.
a, Gating strategy used to analyse the FACS data by FlowJo. First plotted SSC-A vs FSC-A to select the LX-2 cells (in gate: 75.7% ± 4.9% for naïve cells; 73.5% ± 4.5% for perpetuated cells). Secondly, selected the alive cells by plotting PI-A against FSC-A (in gate: 94.0% ± 10.4% for naïve cells; 96.4% ± 4.0% for perpetuated cells) Thirdly excluded all aggregates by selectin only single cells through plotting FSC-W against FSC-A (in gate: 92.0% ± 1.6% for naïve cells; 94.8% ± 1.5% for perpetuated cells). Last, plotted the DiR positive cells in a histogram against APC-Cy7-A. To select the DiR-positive cells, treated LX-2 cells with non-labelled particles and therefore measured no fluorescence with the APC-Cy7 filter. **b,** Comparison of the PLIN2 mRNA transcription change in perpetuated LX-2 cells after treatment with cdNV, cdNV-L, PL, and PL_200 with the cellular particle uptake differences in perpetuated LX-2 cells. Enhanced particle uptake led to bigger PLIN2 mRNA expression increase (mean ± SD, n ≤ 3).

Statistical Analysis

Table S2. Statistical analysis.
Results of one-way ANOVA test with a post-hoc Tukey test of the ORO quantification in Figure 3b and Figure S4b.

Number of families	1				
Number of comparisons per family	28				
Alpha	0.05				
**Tukey's multiple comparisons test**	**Mean Diff.**	**95.00% CI of diff.**	**Significant?**	**Summary**	**Adjusted P Value**
DMEM vs. RolPA	-31.74	-50.82 to -12.67	Yes	***	0.0001
DMEM vs. TGF	-0.768	-20.77 to 19.24	No	ns	>0.9999
DMEM vs. S80	-60.34	-79.42 to -41.27	Yes	****	<0.0001
DMEM vs. S80_X	-9.725	-28.8 to 9.35	No	ns	0.7257
DMEM vs. HEPES	-0.44	-19.51 to 18.63	No	ns	>0.9999
DMEM vs. cdNV_S80	-9.393	-28.47 to 9.682	No	ns	0.7582
DMEM vs. cdNV_TGF	-9.503	-30.83 to 11.82	No	ns	0.8373
RolPA vs. TGF	30.97	10.97 to 50.98	Yes	***	0.0004
RolPA vs. S80	-28.6	-47.67 to -9.525	Yes	***	0.0006
RolPA vs. S80_X	22.02	2.942 to 41.09	Yes	*	0.0143
RolPA vs. HEPES	31.3	12.23 to 50.38	Yes	***	0.0002
RolPA vs. cdNV_S80	22.35	3.273 to 41.42	Yes	*	0.0123
RolPA vs. cdNV_TGF	22.24	0.9128 to 43.57	Yes	*	0.0358
TGF vs. S80	-59.57	-79.58 to -39.57	Yes	****	<0.0001
TGF vs. S80_X	-8.957	-28.96 to 11.05	No	ns	0.834
TGF vs. HEPES	0.328	-19.68 to 20.33	No	ns	>0.9999
TGF vs. cdNV_S80	-8.625	-28.63 to 11.38	No	ns	0.8586
TGF vs. cdNV_TGF	-8.735	-30.9 to 13.43	No	ns	0.9057
S80 vs. S80_X	50.62	31.54 to 69.69	Yes	****	<0.0001
S80 vs. HEPES	59.9	40.83 to 78.98	Yes	****	<0.0001
S80 vs. cdNV_S80	50.95	31.87 to 70.02	Yes	****	<0.0001
S80 vs. cdNV_TGF	50.84	29.51 to 72.17	Yes	****	<0.0001
S80_X vs. HEPES	9.285	-9.79 to 28.36	No	ns	0.7685
S80_X vs. cdNV_S80	0.3317	-18.74 to 19.41	No	ns	>0.9999
S80_X vs. cdNV_TGF	0.2225	-21.1 to 21.55	No	ns	>0.9999
HEPES vs. cdNV_S80	-8.953	-28.03 to 10.12	No	ns	0.7989
HEPES vs. cdNV_TGF	-9.063	-30.39 to 12.26	No	ns	0.8672
cdNV_S80 vs. cdNV_TGF	-0.1092	-21.44 to 21.22	No	ns	>0.9999

Table S3. Statistical analysis.
Results of one-way ANOVA test with a post-hoc Tukey test of COL1A1 mRNA transcription in Figure 3c and Figure S4c.

Number of families	1				
Number of comparisons per family	28				
Alpha	0.05				
Tukey's multiple comparisons test	Mean Diff.	95.00% CI of diff.	Significant?	Summary	Adjusted P Value
cdNV_S80 vs. cdNV_TGF	-0.2136	-2.174 to 1.747	No	ns	>0.9999
cdNV_S80 vs. DMEM	-0.7225	-2.538 to 1.093	No	ns	0.8807
cdNV_S80 vs. RolPA	-0.273	-2.088 to 1.542	No	ns	0.9995
cdNV_S80 vs. TGF	-5.895	-7.71 to -4.079	Yes	****	<0.0001
cdNV_S80 vs. S80	-0.4831	-2.299 to 1.332	No	ns	0.9846
cdNV_S80 vs. S80_X	-0.5451	-2.36 to 1.27	No	ns	0.9701
cdNV_S80 vs. HEPES	-0.6093	-2.425 to 1.206	No	ns	0.9466
cdNV_TGF vs. DMEM	-0.5089	-2.47 to 1.452	No	ns	0.9867
cdNV_TGF vs. RolPA	-0.05941	-2.02 to 1.901	No	ns	>0.9999
cdNV_TGF vs. TGF	-5.681	-7.642 to -3.72	Yes	****	<0.0001
cdNV_TGF vs. S80	-0.2695	-2.23 to 1.691	No	ns	0.9997
cdNV_TGF vs. S80_X	-0.3315	-2.292 to 1.629	No	ns	0.999
cdNV_TGF vs. HEPES	-0.3957	-2.357 to 1.565	No	ns	0.997
DMEM vs. RolPA	0.4495	-1.366 to 2.265	No	ns	0.9899
DMEM vs. TGF	-5.172	-6.988 to -3.357	Yes	****	<0.0001
DMEM vs. S80	0.2394	-1.576 to 2.055	No	ns	0.9998
DMEM vs. S80_X	0.1774	-1.638 to 1.993	No	ns	>0.9999
DMEM vs. HEPES	0.1132	-1.702 to 1.929	No	ns	>0.9999
RolPA vs. TGF	-5.622	-7.437 to -3.806	Yes	****	<0.0001
RolPA vs. S80	-0.2101	-2.025 to 1.605	No	ns	>0.9999
RolPA vs. S80_X	-0.2721	-2.087 to 1.543	No	ns	0.9996
RolPA vs. HEPES	-0.3363	-2.152 to 1.479	No	ns	0.9983
TGF vs. S80	5.412	3.596 to 7.227	Yes	****	<0.0001
TGF vs. S80_X	5.35	3.534 to 7.165	Yes	****	<0.0001
TGF vs. HEPES	5.285	3.47 to 7.101	Yes	****	<0.0001
S80 vs. S80_X	-0.06196	-1.877 to 1.753	No	ns	>0.9999
S80 vs. HEPES	-0.1262	-1.942 to 1.689	No	ns	>0.9999
S80_X vs. HEPES	-0.06423	-1.88 to 1.751	No	ns	>0.9999

Table S4. Statistical analysis.
Results of one-way ANOVA test with a post-hoc Tukey test of SPARC mRNA transcription in Figure 3c and Figure S4c.

Number of families	1				
Number of comparisons per family	28				
Alpha	0.05				
Tukey's multiple comparisons test	Mean Diff.	95.00% CI of diff.	Significant?	Summary	Adjusted P Value
cdNV_S80 vs. cdNV_TGF	-0.2127	-0.8562 to 0.4309	No	ns	0.9506
cdNV_S80 vs. DMEM	-0.4733	-1.069 to 0.1225	No	ns	0.1917

cdNV_S80 vs. RolPA	-0.03878	-0.6346 to 0.557	No	ns	>0.9999
cdNV_S80 vs. TGF	-1.912	-2.508 to -1.316	Yes	****	<0.0001
cdNV_S80 vs. S80	-0.1996	-0.7954 to 0.3961	No	ns	0.947
cdNV_S80 vs. S80_X	-0.4623	-1.058 to 0.1335	No	ns	0.2134
cdNV_S80 vs. HEPES	-0.4234	-1.019 to 0.1724	No	ns	0.3046
cdNV_TGF vs. DMEM	-0.2607	-0.9042 to 0.3829	No	ns	0.8712
cdNV_TGF vs. RolPA	0.1739	-0.4697 to 0.8174	No	ns	0.9833
cdNV_TGF vs. TGF	-1.699	-2.343 to -1.056	Yes	****	<0.0001
cdNV_TGF vs. S80	0.01301	-0.6305 to 0.6565	No	ns	>0.9999
cdNV_TGF vs. S80_X	-0.2496	-0.8932 to 0.3939	No	ns	0.8935
cdNV_TGF vs. HEPES	-0.2107	-0.8542 to 0.4328	No	ns	0.9529
DMEM vs. RolPA	0.4345	-0.1613 to 1.03	No	ns	0.2761
DMEM vs. TGF	-1.439	-2.034 to -0.8429	Yes	****	<0.0001
DMEM vs. S80	0.2737	-0.3221 to 0.8695	No	ns	0.7856
DMEM vs. S80_X	0.01102	-0.5848 to 0.6068	No	ns	>0.9999
DMEM vs. HEPES	0.04995	-0.5458 to 0.6457	No	ns	>0.9999
RolPA vs. TGF	-1.873	-2.469 to -1.277	Yes	****	<0.0001
RolPA vs. S80	-0.1609	-0.7567 to 0.4349	No	ns	0.9833
RolPA vs. S80_X	-0.4235	-1.019 to 0.1723	No	ns	0.3042
RolPA vs. HEPES	-0.3846	-0.9804 to 0.2112	No	ns	0.4172
TGF vs. S80	1.712	1.117 to 2.308	Yes	****	<0.0001
TGF vs. S80_X	1.45	0.8539 to 2.045	Yes	****	<0.0001
TGF vs. HEPES	1.489	0.8928 to 2.084	Yes	****	<0.0001
S80 vs. S80_X	-0.2627	-0.8584 to 0.3331	No	ns	0.8171
S80 vs. HEPES	-0.2237	-0.8195 to 0.3721	No	ns	0.9082
S80_X vs. HEPES	0.03893	-0.5569 to 0.6347	No	ns	>0.9999

Table S5. Statistical analysis.
Results of one-way ANOVA test with a post-hoc Tukey test of PLIN2 mRNA transcription in Figure 3c and Figure S4c.

**Number of families**	1				
**Number of comparisons per family**	28				
**Alpha**	0.05				
**Tukey's multiple comparisons test**	**Mean Diff.**	**95.00% CI of diff.**	**Significant?**	**Summary**	**Adjusted P Value**
cdNV_S80 vs. cdNV_TGF	-0.07485	-1.03 to 0.8806	No	ns	>0.9999
cdNV_S80 vs. DMEM	0.4461	-0.4477 to 1.34	No	ns	0.7072
cdNV_S80 vs. RolPA	-1.669	-2.562 to -0.7749	Yes	****	<0.0001
cdNV_S80 vs. TGF	0.7537	-0.1401 to 1.647	No	ns	0.1416
cdNV_S80 vs. S80	-1.085	-1.979 to -0.191	Yes	*	0.0104
cdNV_S80 vs. S80_X	-0.02173	-0.9155 to 0.872	No	ns	>0.9999
cdNV_S80 vs. HEPES	0.5786	-0.3152 to 1.472	No	ns	0.4099
cdNV_TGF vs. DMEM	0.521	-0.3728 to 1.415	No	ns	0.5365
cdNV_TGF vs. RolPA	-1.594	-2.488 to -0.7	Yes	***	0.0001
cdNV_TGF vs. TGF	0.8285	-0.06525 to 1.722	No	ns	0.0825
cdNV_TGF vs. S80	-1.01	-1.904 to -0.1161	Yes	*	0.0195
cdNV_TGF vs. S80_X	0.05312	-0.8406 to 0.9469	No	ns	>0.9999

cdNV_TGF vs. HEPES	0.6535	-0.2403 to 1.547	No	ns	0.2709
DMEM vs. RolPA	-2.115	-2.942 to -1.287	Yes	****	<0.0001
DMEM vs. TGF	0.3076	-0.5199 to 1.135	No	ns	0.9104
DMEM vs. S80	-1.531	-2.358 to -0.7034	Yes	****	<0.0001
DMEM vs. S80_X	-0.4678	-1.295 to 0.3596	No	ns	0.5724
DMEM vs. HEPES	0.1325	-0.695 to 0.96	No	ns	0.9993
RolPA vs. TGF	2.422	1.595 to 3.25	Yes	****	<0.0001
RolPA vs. S80	0.5839	-0.2436 to 1.411	No	ns	0.3095
RolPA vs. S80_X	1.647	0.8194 to 2.474	Yes	****	<0.0001
RolPA vs. HEPES	2.247	1.42 to 3.075	Yes	****	<0.0001
TGF vs. S80	-1.838	-2.666 to -1.011	Yes	****	<0.0001
TGF vs. S80_X	-0.7754	-1.603 to 0.05207	No	ns	0.0771
TGF vs. HEPES	-0.175	-1.003 to 0.6524	No	ns	0.9959
S80 vs. S80_X	1.063	0.2356 to 1.89	Yes	**	0.006
S80 vs. HEPES	1.663	0.8359 to 2.491	Yes	****	<0.0001
S80_X vs. HEPES	0.6003	-0.2271 to 1.428	No	ns	0.2791

Table S6. Statistical analysis.
Results of one-way ANOVA test with a post-hoc Tukey test of the cell viability in Figure 4a.

Number of families	1				
Number of comparisons per family	435				
Alpha	0.05				
Tukey's multiple comparisons test	Mean Diff.	95.00% CI of diff.	Significant?	Summary	Adjusted P Value
DMEM vs. RolPA	7.653	-12.25 to 27.55	No	ns	0.9996
DMEM vs. TGF-ß1	5.346	-14.56 to 25.25	No	ns	>0.9999
DMEM vs. S80	19.71	-5.588 to 45	No	ns	0.3873
DMEM vs. PBS	5.777	-14.88 to 26.43	No	ns	>0.9999
DMEM vs. HEPES	0.774	-20.89 to 22.43	No	ns	>0.9999
DMEM vs. cdNV_S80_A	-9.407	-34.7 to 15.89	No	ns	0.9998
DMEM vs. cdNV_RolPA_A	-21.21	-44.3 to 1.881	No	ns	0.1192
DMEM vs. cdNV_TGF_A	-33.11	-56.2 to -10.02	Yes	***	0.0001
DMEM vs. cdNV_DMEM_A	-11.41	-36.7 to 13.88	No	ns	0.9949
DMEM vs. cdNV_S80_B	-0.7167	-26.01 to 24.58	No	ns	>0.9999
DMEM vs. cdNV_RolPA_B	-7.565	-30.66 to 15.53	No	ns	>0.9999
DMEM vs. cdNV_TGF_B	-8.42	-31.51 to 14.67	No	ns	0.9998
DMEM vs. cdNV_DMEM_B	11.56	-13.74 to 36.85	No	ns	0.9938
DMEM vs. cdNV_S80_C	16.39	-8.908 to 41.68	No	ns	0.7577
DMEM vs. cdNV_RolPA_C	-0.15	-23.24 to 22.94	No	ns	>0.9999
DMEM vs. cdNV_TGF_C	-0.9325	-24.02 to 22.16	No	ns	>0.9999
DMEM vs. cdNV_DMEM_C	7.527	-17.77 to 32.82	No	ns	>0.9999
DMEM vs. sn_DMEM_a	-7.873	-33.17 to 17.42	No	ns	>0.9999
DMEM vs. sn_DMEM_b	-8.708	-34 to 16.59	No	ns	>0.9999
DMEM vs. sn_DMEM_c	3.202	-22.09 to 28.5	No	ns	>0.9999
DMEM vs. sn_RolPA_a	-1.075	-26.37 to 24.22	No	ns	>0.9999
DMEM vs. sn_RolPA_b	1.306	-23.99 to 26.6	No	ns	>0.9999
DMEM vs. sn_RolPA_c	1.211	-24.08 to 26.51	No	ns	>0.9999
DMEM vs. sn_TGF_a	10.79	-14.5 to 36.09	No	ns	0.9978
DMEM vs. sn_TGF_b	4.256	-21.04 to 29.55	No	ns	>0.9999
DMEM vs. sn_TGF_c	6.991	-18.3 to 32.29	No	ns	>0.9999
DMEM vs. sn_S80_a	-11.63	-36.93 to 13.66	No	ns	0.9932
DMEM vs. sn_S80_b	-7.008	-32.3 to 18.29	No	ns	>0.9999
DMEM vs. sn_S80_c	-0.4956	-25.79 to 24.8	No	ns	>0.9999
RolPA vs. TGF-ß1	-2.307	-21.43 to 16.81	No	ns	>0.9999
RolPA vs. S80	12.05	-12.63 to 36.74	No	ns	0.9849
RolPA vs. PBS	-1.876	-21.78 to 18.03	No	ns	>0.9999
RolPA vs. HEPES	-6.879	-27.82 to 14.07	No	ns	>0.9999
RolPA vs. cdNV_S80_A	-17.06	-41.74 to 7.625	No	ns	0.639
RolPA vs. cdNV_RolPA_A	-28.86	-51.28 to -6.442	Yes	**	0.0011

RolPA vs. cdNV_TGF_A	-40.76	-63.18 to -18.34	Yes	****	<0.0001
RolPA vs. cdNV_DMEM_A	-19.06	-43.75 to 5.622	No	ns	0.4056
RolPA vs. cdNV_S80_B	-8.37	-33.05 to 16.32	No	ns	>0.9999
RolPA vs. cdNV_RolPA_B	-15.22	-37.64 to 7.203	No	ns	0.6743
RolPA vs. cdNV_TGF_B	-16.07	-38.49 to 6.348	No	ns	0.5637
RolPA vs. cdNV_DMEM_B	3.904	-20.78 to 28.59	No	ns	>0.9999
RolPA vs. cdNV_S80_C	8.734	-15.95 to 33.42	No	ns	>0.9999
RolPA vs. cdNV_RolPA_C	-7.803	-30.22 to 14.62	No	ns	>0.9999
RolPA vs. cdNV_TGF_C	-8.585	-31.01 to 13.84	No	ns	0.9996
RolPA vs. cdNV_DMEM_C	-0.1262	-24.81 to 24.56	No	ns	>0.9999
RolPA vs. sn_DMEM_a	-15.53	-40.21 to 9.159	No	ns	0.8038
RolPA vs. sn_DMEM_b	-16.36	-41.05 to 8.324	No	ns	0.7184
RolPA vs. sn_DMEM_c	-4.451	-29.14 to 20.23	No	ns	>0.9999
RolPA vs. sn_RolPA_a	-8.728	-33.41 to 15.96	No	ns	>0.9999
RolPA vs. sn_RolPA_b	-6.347	-31.03 to 18.34	No	ns	>0.9999
RolPA vs. sn_RolPA_c	-6.442	-31.13 to 18.24	No	ns	>0.9999
RolPA vs. sn_TGF_a	3.14	-21.54 to 27.82	No	ns	>0.9999
RolPA vs. sn_TGF_b	-3.396	-28.08 to 21.29	No	ns	>0.9999
RolPA vs. sn_TGF_c	-0.6617	-25.35 to 24.02	No	ns	>0.9999
RolPA vs. sn_S80_a	-19.29	-43.97 to 5.397	No	ns	0.3814
RolPA vs. sn_S80_b	-14.66	-39.35 to 10.02	No	ns	0.8762
RolPA vs. sn_S80_c	-8.148	-32.83 to 16.54	No	ns	>0.9999
TGF-ß1 vs. S80	14.36	-10.32 to 39.05	No	ns	0.897
TGF-ß1 vs. PBS	0.431	-19.47 to 20.33	No	ns	>0.9999
TGF-ß1 vs. HEPES	-4.572	-25.52 to 16.37	No	ns	>0.9999
TGF-ß1 vs. cdNV_S80_A	-14.75	-39.44 to 9.932	No	ns	0.8695
TGF-ß1 vs. cdNV_RolPA_A	-26.56	-48.98 to -4.135	Yes	**	0.0049
TGF-ß1 vs. cdNV_TGF_A	-38.46	-60.88 to -16.03	Yes	****	<0.0001
TGF-ß1 vs. cdNV_DMEM_A	-16.76	-41.44 to 7.929	No	ns	0.6742
TGF-ß1 vs. cdNV_S80_B	-6.062	-30.75 to 18.62	No	ns	>0.9999
TGF-ß1 vs. cdNV_RolPA_B	-12.91	-35.33 to 9.51	No	ns	0.9063
TGF-ß1 vs. cdNV_TGF_B	-13.77	-36.19 to 8.655	No	ns	0.837
TGF-ß1 vs. cdNV_DMEM_B	6.211	-18.47 to 30.9	No	ns	>0.9999
TGF-ß1 vs. cdNV_S80_C	11.04	-13.64 to 35.73	No	ns	0.9954
TGF-ß1 vs. cdNV_RolPA_C	-5.496	-27.92 to 16.93	No	ns	>0.9999
TGF-ß1 vs. cdNV_TGF_C	-6.278	-28.7 to 16.14	No	ns	>0.9999
TGF-ß1 vs. cdNV_DMEM_C	2.181	-22.5 to 26.87	No	ns	>0.9999
TGF-ß1 vs. sn_DMEM_a	-13.22	-37.9 to 11.47	No	ns	0.9549
TGF-ß1 vs. sn_DMEM_b	-14.05	-38.74 to 10.63	No	ns	0.9158
TGF-ß1 vs. sn_DMEM_c	-2.144	-26.83 to 22.54	No	ns	>0.9999
TGF-ß1 vs. sn_RolPA_a	-6.421	-31.11 to 18.26	No	ns	>0.9999
TGF-ß1 vs. sn_RolPA_b	-4.04	-28.72 to 20.65	No	ns	>0.9999
TGF-ß1 vs. sn_RolPA_c	-4.135	-28.82 to 20.55	No	ns	>0.9999
TGF-ß1 vs. sn_TGF_a	5.447	-19.24 to 30.13	No	ns	>0.9999
TGF-ß1 vs. sn_TGF_b	-1.089	-25.77 to 23.6	No	ns	>0.9999

TGF-ß1 vs. sn_TGF_c	1.645	-23.04 to 26.33	No	ns	>0.9999
TGF-ß1 vs. sn_S80_a	-16.98	-41.67 to 7.704	No	ns	0.6482
TGF-ß1 vs. sn_S80_b	-12.35	-37.04 to 12.33	No	ns	0.9794
TGF-ß1 vs. sn_S80_c	-5.841	-30.53 to 18.84	No	ns	>0.9999
S80 vs. PBS	-13.93	-39.22 to 11.36	No	ns	0.9394
S80 vs. HEPES	-18.93	-45.06 to 7.191	No	ns	0.5407
S80 vs. cdNV_S80_A	-29.11	-58.32 to 0.09418	No	ns	0.0519
S80 vs. cdNV_RolPA_A	-40.92	-68.24 to -13.6	Yes	****	<0.0001
S80 vs. cdNV_TGF_A	-52.82	-80.14 to -25.5	Yes	****	<0.0001
S80 vs. cdNV_DMEM_A	-31.12	-60.32 to -1.909	Yes	*	0.0231
S80 vs. cdNV_S80_B	-20.42	-49.63 to 8.784	No	ns	0.6153
S80 vs. cdNV_RolPA_B	-27.27	-54.59 to 0.04946	No	ns	0.051
S80 vs. cdNV_TGF_B	-28.13	-55.45 to -0.8055	Yes	*	0.0355
S80 vs. cdNV_DMEM_B	-8.15	-37.36 to 21.06	No	ns	>0.9999
S80 vs. cdNV_S80_C	-3.32	-32.53 to 25.89	No	ns	>0.9999
S80 vs. cdNV_RolPA_C	-19.86	-47.18 to 7.464	No	ns	0.5346
S80 vs. cdNV_TGF_C	-20.64	-47.96 to 6.682	No	ns	0.4521
S80 vs. cdNV_DMEM_C	-12.18	-41.39 to 17.03	No	ns	0.9984
S80 vs. sn_DMEM_a	-27.58	-56.79 to 1.628	No	ns	0.0917
S80 vs. sn_DMEM_b	-28.41	-57.62 to 0.7929	No	ns	0.0676
S80 vs. sn_DMEM_c	-16.5	-45.71 to 12.7	No	ns	0.9216
S80 vs. sn_RolPA_a	-20.78	-49.99 to 8.426	No	ns	0.5794
S80 vs. sn_RolPA_b	-18.4	-47.61 to 10.81	No	ns	0.8014
S80 vs. sn_RolPA_c	-18.5	-47.7 to 10.71	No	ns	0.7937
S80 vs. sn_TGF_a	-8.914	-38.12 to 20.29	No	ns	>0.9999
S80 vs. sn_TGF_b	-15.45	-44.66 to 13.76	No	ns	0.9606
S80 vs. sn_TGF_c	-12.72	-41.92 to 16.49	No	ns	0.997
S80 vs. sn_S80_a	-31.34	-60.55 to -2.134	Yes	*	0.021
S80 vs. sn_S80_b	-26.71	-55.92 to 2.493	No	ns	0.1238
S80 vs. sn_S80_c	-20.2	-49.41 to 9.005	No	ns	0.6373
PBS vs. HEPES	-5.003	-26.66 to 16.66	No	ns	>0.9999
PBS vs. cdNV_S80_A	-15.18	-40.48 to 10.11	No	ns	0.8645
PBS vs. cdNV_RolPA_A	-26.99	-50.08 to -3.896	Yes	**	0.006
PBS vs. cdNV_TGF_A	-38.89	-61.98 to -15.8	Yes	****	<0.0001
PBS vs. cdNV_DMEM_A	-17.19	-42.48 to 8.108	No	ns	0.6723
PBS vs. cdNV_S80_B	-6.493	-31.79 to 18.8	No	ns	>0.9999
PBS vs. cdNV_RolPA_B	-13.34	-36.43 to 9.749	No	ns	0.9033
PBS vs. cdNV_TGF_B	-14.2	-37.29 to 8.894	No	ns	0.8352
PBS vs. cdNV_DMEM_B	5.78	-19.51 to 31.07	No	ns	>0.9999
PBS vs. cdNV_S80_C	10.61	-14.68 to 35.9	No	ns	0.9983
PBS vs. cdNV_RolPA_C	-5.927	-29.02 to 17.16	No	ns	>0.9999
PBS vs. cdNV_TGF_C	-6.709	-29.8 to 16.38	No	ns	>0.9999
PBS vs. cdNV_DMEM_C	1.75	-23.54 to 27.04	No	ns	>0.9999
PBS vs. sn_DMEM_a	-13.65	-38.94 to 11.64	No	ns	0.951
PBS vs. sn_DMEM_b	-14.48	-39.78 to 10.81	No	ns	0.911

PBS vs. sn_DMEM_c	-2.575	-27.87 to 22.72	No	ns	>0.9999
PBS vs. sn_RolPA_a	-6.852	-32.15 to 18.44	No	ns	>0.9999
PBS vs. sn_RolPA_b	-4.471	-29.77 to 20.82	No	ns	>0.9999
PBS vs. sn_RolPA_c	-4.566	-29.86 to 20.73	No	ns	>0.9999
PBS vs. sn_TGF_a	5.016	-20.28 to 30.31	No	ns	>0.9999
PBS vs. sn_TGF_b	-1.52	-26.81 to 23.77	No	ns	>0.9999
PBS vs. sn_TGF_c	1.214	-24.08 to 26.51	No	ns	>0.9999
PBS vs. sn_S80_a	-17.41	-42.71 to 7.883	No	ns	0.6469
PBS vs. sn_S80_b	-12.78	-38.08 to 12.51	No	ns	0.9768
PBS vs. sn_S80_c	-6.272	-31.57 to 19.02	No	ns	>0.9999
HEPES vs. cdNV_S80_A	-10.18	-36.3 to 15.94	No	ns	0.9995
HEPES vs. cdNV_RolPA_A	-21.98	-45.98 to 2.012	No	ns	0.122
HEPES vs. cdNV_TGF_A	-33.88	-57.88 to -9.888	Yes	***	0.0002
HEPES vs. cdNV_DMEM_A	-12.18	-38.31 to 13.94	No	ns	0.9918
HEPES vs. cdNV_S80_B	-1.491	-27.61 to 24.63	No	ns	>0.9999
HEPES vs. cdNV_RolPA_B	-8.339	-32.34 to 15.66	No	ns	>0.9999
HEPES vs. cdNV_TGF_B	-9.194	-33.19 to 14.8	No	ns	0.9996
HEPES vs. cdNV_DMEM_B	10.78	-15.34 to 36.91	No	ns	0.9987
HEPES vs. cdNV_S80_C	15.61	-10.51 to 41.74	No	ns	0.8695
HEPES vs. cdNV_RolPA_C	-0.924	-24.92 to 23.07	No	ns	>0.9999
HEPES vs. cdNV_TGF_C	-1.707	-25.7 to 22.29	No	ns	>0.9999
HEPES vs. cdNV_DMEM_C	6.753	-19.37 to 32.88	No	ns	>0.9999
HEPES vs. sn_DMEM_a	-8.647	-34.77 to 17.48	No	ns	>0.9999
HEPES vs. sn_DMEM_b	-9.482	-35.61 to 16.64	No	ns	0.9999
HEPES vs. sn_DMEM_c	2.428	-23.7 to 28.55	No	ns	>0.9999
HEPES vs. sn_RolPA_a	-1.849	-27.97 to 24.27	No	ns	>0.9999
HEPES vs. sn_RolPA_b	0.5319	-25.59 to 26.66	No	ns	>0.9999
HEPES vs. sn_RolPA_c	0.4372	-25.69 to 26.56	No	ns	>0.9999
HEPES vs. sn_TGF_a	10.02	-16.11 to 36.14	No	ns	0.9996
HEPES vs. sn_TGF_b	3.482	-22.64 to 29.61	No	ns	>0.9999
HEPES vs. sn_TGF_c	6.217	-19.91 to 32.34	No	ns	>0.9999
HEPES vs. sn_S80_a	-12.41	-38.53 to 13.72	No	ns	0.9895
HEPES vs. sn_S80_b	-7.782	-33.91 to 18.34	No	ns	>0.9999
HEPES vs. sn_S80_c	-1.27	-27.39 to 24.85	No	ns	>0.9999
cdNV_S80_A vs. cdNV_RolPA_A	-11.8	-39.12 to 15.52	No	ns	0.9973
cdNV_S80_A vs. cdNV_TGF_A	-23.7	-51.02 to 3.618	No	ns	0.1926
cdNV_S80_A vs. cdNV_DMEM_A	-2.003	-31.21 to 27.2	No	ns	>0.9999
cdNV_S80_A vs. cdNV_S80_B	8.69	-20.52 to 37.9	No	ns	>0.9999
cdNV_S80_A vs. cdNV_RolPA_B	1.842	-25.48 to 29.16	No	ns	>0.9999
cdNV_S80_A vs. cdNV_TGF_B	0.9867	-26.33 to 28.31	No	ns	>0.9999
cdNV_S80_A vs. cdNV_DMEM_B	20.96	-8.244 to 50.17	No	ns	0.5612
cdNV_S80_A vs. cdNV_S80_C	25.79	-3.414 to 55	No	ns	0.1673
cdNV_S80_A vs. cdNV_RolPA_C	9.257	-18.06 to 36.58	No	ns	>0.9999
cdNV_S80_A vs. cdNV_TGF_C	8.474	-18.85 to 35.8	No	ns	>0.9999
cdNV_S80_A vs. cdNV_DMEM_C	16.93	-12.27 to 46.14	No	ns	0.9002

cdNV_S80_A vs. sn_DMEM_a	1.534	-27.67 to 30.74	No	ns	>0.9999
cdNV_S80_A vs. sn_DMEM_b	0.6988	-28.51 to 29.91	No	ns	>0.9999
cdNV_S80_A vs. sn_DMEM_c	12.61	-16.6 to 41.82	No	ns	0.9973
cdNV_S80_A vs. sn_RolPA_a	8.331	-20.88 to 37.54	No	ns	>0.9999
cdNV_S80_A vs. sn_RolPA_b	10.71	-18.49 to 39.92	No	ns	0.9998
cdNV_S80_A vs. sn_RolPA_c	10.62	-18.59 to 39.83	No	ns	0.9999
cdNV_S80_A vs. sn_TGF_a	20.2	-9.008 to 49.41	No	ns	0.6376
cdNV_S80_A vs. sn_TGF_b	13.66	-15.54 to 42.87	No	ns	0.9914
cdNV_S80_A vs. sn_TGF_c	16.4	-12.81 to 45.61	No	ns	0.9265
cdNV_S80_A vs. sn_S80_a	-2.228	-31.44 to 26.98	No	ns	>0.9999
cdNV_S80_A vs. sn_S80_b	2.398	-26.81 to 31.61	No	ns	>0.9999
cdNV_S80_A vs. sn_S80_c	8.911	-20.3 to 38.12	No	ns	>0.9999
cdNV_RolPA_A vs. cdNV_TGF_A	-11.9	-37.19 to 13.39	No	ns	0.9907
cdNV_RolPA_A vs. cdNV_DMEM_A	9.8	-17.52 to 37.12	No	ns	0.9999
cdNV_RolPA_A vs. cdNV_S80_B	20.49	-6.828 to 47.81	No	ns	0.4672
cdNV_RolPA_A vs. cdNV_RolPA_B	13.65	-11.65 to 38.94	No	ns	0.9512
cdNV_RolPA_A vs. cdNV_TGF_B	12.79	-12.5 to 38.08	No	ns	0.9767
cdNV_RolPA_A vs. cdNV_DMEM_B	32.77	5.446 to 60.09	Yes	**	0.004
cdNV_RolPA_A vs. cdNV_S80_C	37.6	10.28 to 64.92	Yes	***	0.0003
cdNV_RolPA_A vs. cdNV_RolPA_C	21.06	-4.234 to 46.35	No	ns	0.2595
cdNV_RolPA_A vs. cdNV_TGF_C	20.28	-5.017 to 45.57	No	ns	0.3299
cdNV_RolPA_A vs. cdNV_DMEM_C	28.74	1.416 to 56.06	Yes	*	0.0272
cdNV_RolPA_A vs. sn_DMEM_a	13.34	-13.98 to 40.66	No	ns	0.9849
cdNV_RolPA_A vs. sn_DMEM_b	12.5	-14.82 to 39.82	No	ns	0.9937
cdNV_RolPA_A vs. sn_DMEM_c	24.41	-2.909 to 51.73	No	ns	0.1519
cdNV_RolPA_A vs. sn_RolPA_a	20.13	-7.186 to 47.46	No	ns	0.5049
cdNV_RolPA_A vs. sn_RolPA_b	22.52	-4.805 to 49.84	No	ns	0.2777
cdNV_RolPA_A vs. sn_RolPA_c	22.42	-4.9 to 49.74	No	ns	0.2854
cdNV_RolPA_A vs. sn_TGF_a	32	4.682 to 59.32	Yes	**	0.0058
cdNV_RolPA_A vs. sn_TGF_b	25.47	-1.855 to 52.79	No	ns	0.104
cdNV_RolPA_A vs. sn_TGF_c	28.2	0.88 to 55.52	Yes	*	0.0344
cdNV_RolPA_A vs. sn_S80_a	9.575	-17.75 to 36.9	No	ns	>0.9999
cdNV_RolPA_A vs. sn_S80_b	14.2	-13.12 to 41.52	No	ns	0.9677
cdNV_RolPA_A vs. sn_S80_c	20.71	-6.607 to 48.04	No	ns	0.4443
cdNV_TGF_A vs. cdNV_DMEM_A	21.7	-5.621 to 49.02	No	ns	0.3482
cdNV_TGF_A vs. cdNV_S80_B	32.39	5.072 to 59.71	Yes	**	0.0048
cdNV_TGF_A vs. cdNV_RolPA_B	25.55	0.2506 to 50.84	Yes	*	0.0446
cdNV_TGF_A vs. cdNV_TGF_B	24.69	-0.6044 to 49.98	No	ns	0.0653
cdNV_TGF_A vs. cdNV_DMEM_B	44.67	17.35 to 71.99	Yes	****	<0.0001
cdNV_TGF_A vs. cdNV_S80_C	49.5	22.18 to 76.82	Yes	****	<0.0001
cdNV_TGF_A vs. cdNV_RolPA_C	32.96	7.666 to 58.25	Yes	***	0.0009
cdNV_TGF_A vs. cdNV_TGF_C	32.18	6.883 to 57.47	Yes	**	0.0014
cdNV_TGF_A vs. cdNV_DMEM_C	40.64	13.32 to 67.96	Yes	****	<0.0001
cdNV_TGF_A vs. sn_DMEM_a	25.24	-2.084 to 52.56	No	ns	0.1132
cdNV_TGF_A vs. sn_DMEM_b	24.4	-2.919 to 51.72	No	ns	0.1524

cdNV_TGF_A vs. sn_DMEM_c	36.31	8.991 to 63.63	Yes	***	0.0006
cdNV_TGF_A vs. sn_RolPA_a	32.03	4.714 to 59.36	Yes	**	0.0057
cdNV_TGF_A vs. sn_RolPA_b	34.42	7.095 to 61.74	Yes	**	0.0017
cdNV_TGF_A vs. sn_RolPA_c	34.32	7 to 61.64	Yes	**	0.0018
cdNV_TGF_A vs. sn_TGF_a	43.9	16.58 to 71.22	Yes	****	<0.0001
cdNV_TGF_A vs. sn_TGF_b	37.37	10.05 to 64.69	Yes	***	0.0003
cdNV_TGF_A vs. sn_TGF_c	40.1	12.78 to 67.42	Yes	****	<0.0001
cdNV_TGF_A vs. sn_S80_a	21.48	-5.846 to 48.8	No	ns	0.3692
cdNV_TGF_A vs. sn_S80_b	26.1	-1.219 to 53.42	No	ns	0.0816
cdNV_TGF_A vs. sn_S80_c	32.61	5.293 to 59.94	Yes	**	0.0043
cdNV_DMEM_A vs. cdNV_S80_B	10.69	-18.51 to 39.9	No	ns	0.9998
cdNV_DMEM_A vs. cdNV_RolPA_B	3.845	-23.48 to 31.17	No	ns	>0.9999
cdNV_DMEM_A vs. cdNV_TGF_B	2.99	-24.33 to 30.31	No	ns	>0.9999
cdNV_DMEM_A vs. cdNV_DMEM_B	22.97	-6.241 to 52.17	No	ns	0.3684
cdNV_DMEM_A vs. cdNV_S80_C	27.8	-1.411 to 57	No	ns	0.0849
cdNV_DMEM_A vs. cdNV_RolPA_C	11.26	-16.06 to 38.58	No	ns	0.9987
cdNV_DMEM_A vs. cdNV_TGF_C	10.48	-16.84 to 37.8	No	ns	0.9996
cdNV_DMEM_A vs. cdNV_DMEM_C	18.94	-10.27 to 48.14	No	ns	0.7564
cdNV_DMEM_A vs. sn_DMEM_a	3.537	-25.67 to 32.74	No	ns	>0.9999
cdNV_DMEM_A vs. sn_DMEM_b	2.702	-26.51 to 31.91	No	ns	>0.9999
cdNV_DMEM_A vs. sn_DMEM_c	14.61	-14.6 to 43.82	No	ns	0.9795
cdNV_DMEM_A vs. sn_RolPA_a	10.33	-18.87 to 39.54	No	ns	>0.9999
cdNV_DMEM_A vs. sn_RolPA_b	12.72	-16.49 to 41.92	No	ns	0.997
cdNV_DMEM_A vs. sn_RolPA_c	12.62	-16.59 to 41.83	No	ns	0.9973
cdNV_DMEM_A vs. sn_TGF_a	22.2	-7.005 to 51.41	No	ns	0.4388
cdNV_DMEM_A vs. sn_TGF_b	15.67	-13.54 to 44.87	No	ns	0.9541
cdNV_DMEM_A vs. sn_TGF_c	18.4	-10.81 to 47.61	No	ns	0.8013
cdNV_DMEM_A vs. sn_S80_a	-0.2247	-29.43 to 28.98	No	ns	>0.9999
cdNV_DMEM_A vs. sn_S80_b	4.402	-24.81 to 33.61	No	ns	>0.9999
cdNV_DMEM_A vs. sn_S80_c	10.91	-18.29 to 40.12	No	ns	0.9998
cdNV_S80_B vs. cdNV_RolPA_B	-6.848	-34.17 to 20.47	No	ns	>0.9999
cdNV_S80_B vs. cdNV_TGF_B	-7.703	-35.02 to 19.62	No	ns	>0.9999
cdNV_S80_B vs. cdNV_DMEM_B	12.27	-16.93 to 41.48	No	ns	0.9982
cdNV_S80_B vs. cdNV_S80_C	17.1	-12.1 to 46.31	No	ns	0.8907
cdNV_S80_B vs. cdNV_RolPA_C	0.5667	-26.75 to 27.89	No	ns	>0.9999
cdNV_S80_B vs. cdNV_TGF_C	-0.2158	-27.54 to 27.11	No	ns	>0.9999
cdNV_S80_B vs. cdNV_DMEM_C	8.243	-20.96 to 37.45	No	ns	>0.9999
cdNV_S80_B vs. sn_DMEM_a	-7.156	-36.36 to 22.05	No	ns	>0.9999
cdNV_S80_B vs. sn_DMEM_b	-7.991	-37.2 to 21.22	No	ns	>0.9999
cdNV_S80_B vs. sn_DMEM_c	3.918	-25.29 to 33.13	No	ns	>0.9999
cdNV_S80_B vs. sn_RolPA_a	-0.3586	-29.57 to 28.85	No	ns	>0.9999
cdNV_S80_B vs. sn_RolPA_b	2.023	-27.18 to 31.23	No	ns	>0.9999
cdNV_S80_B vs. sn_RolPA_c	1.928	-27.28 to 31.14	No	ns	>0.9999
cdNV_S80_B vs. sn_TGF_a	11.51	-17.7 to 40.72	No	ns	0.9994
cdNV_S80_B vs. sn_TGF_b	4.973	-24.23 to 34.18	No	ns	>0.9999

cdNV_S80_B vs. sn_TGF_c	7.708	-21.5 to 36.92	No	ns	>0.9999
cdNV_S80_B vs. sn_S80_a	-10.92	-40.13 to 18.29	No	ns	0.9998
cdNV_S80_B vs. sn_S80_b	-6.292	-35.5 to 22.92	No	ns	>0.9999
cdNV_S80_B vs. sn_S80_c	0.2211	-28.99 to 29.43	No	ns	>0.9999
cdNV_RolPA_B vs. cdNV_TGF_B	-0.855	-26.15 to 24.44	No	ns	>0.9999
cdNV_RolPA_B vs. cdNV_DMEM_B	19.12	-8.199 to 46.44	No	ns	0.6135
cdNV_RolPA_B vs. cdNV_S80_C	23.95	-3.369 to 51.27	No	ns	0.1775
cdNV_RolPA_B vs. cdNV_RolPA_C	7.415	-17.88 to 32.71	No	ns	>0.9999
cdNV_RolPA_B vs. cdNV_TGF_C	6.632	-18.66 to 31.93	No	ns	>0.9999
cdNV_RolPA_B vs. cdNV_DMEM_C	15.09	-12.23 to 42.41	No	ns	0.9375
cdNV_RolPA_B vs. sn_DMEM_a	-0.308	-27.63 to 27.01	No	ns	>0.9999
cdNV_RolPA_B vs. sn_DMEM_b	-1.143	-28.46 to 26.18	No	ns	>0.9999
cdNV_RolPA_B vs. sn_DMEM_c	10.77	-16.55 to 38.09	No	ns	0.9994
cdNV_RolPA_B vs. sn_RolPA_a	6.49	-20.83 to 33.81	No	ns	>0.9999
cdNV_RolPA_B vs. sn_RolPA_b	8.871	-18.45 to 36.19	No	ns	>0.9999
cdNV_RolPA_B vs. sn_RolPA_c	8.776	-18.54 to 36.1	No	ns	>0.9999
cdNV_RolPA_B vs. sn_TGF_a	18.36	-8.963 to 45.68	No	ns	0.6934
cdNV_RolPA_B vs. sn_TGF_b	11.82	-15.5 to 39.14	No	ns	0.9972
cdNV_RolPA_B vs. sn_TGF_c	14.56	-12.76 to 41.88	No	ns	0.9574
cdNV_RolPA_B vs. sn_S80_a	-4.07	-31.39 to 23.25	No	ns	>0.9999
cdNV_RolPA_B vs. sn_S80_b	0.5568	-26.76 to 27.88	No	ns	>0.9999
cdNV_RolPA_B vs. sn_S80_c	7.069	-20.25 to 34.39	No	ns	>0.9999
cdNV_TGF_B vs. cdNV_DMEM_B	19.98	-7.344 to 47.3	No	ns	0.5218
cdNV_TGF_B vs. cdNV_S80_C	24.81	-2.514 to 52.13	No	ns	0.1322
cdNV_TGF_B vs. cdNV_RolPA_C	8.27	-17.02 to 33.56	No	ns	>0.9999
cdNV_TGF_B vs. cdNV_TGF_C	7.488	-17.81 to 32.78	No	ns	>0.9999
cdNV_TGF_B vs. cdNV_DMEM_C	15.95	-11.37 to 43.27	No	ns	0.8939
cdNV_TGF_B vs. sn_DMEM_a	0.547	-26.77 to 27.87	No	ns	>0.9999
cdNV_TGF_B vs. sn_DMEM_b	-0.2879	-27.61 to 27.03	No	ns	>0.9999
cdNV_TGF_B vs. sn_DMEM_c	11.62	-15.7 to 38.94	No	ns	0.9979
cdNV_TGF_B vs. sn_RolPA_a	7.345	-19.98 to 34.67	No	ns	>0.9999
cdNV_TGF_B vs. sn_RolPA_b	9.726	-17.6 to 37.05	No	ns	0.9999
cdNV_TGF_B vs. sn_RolPA_c	9.631	-17.69 to 36.95	No	ns	>0.9999
cdNV_TGF_B vs. sn_TGF_a	19.21	-8.108 to 46.53	No	ns	0.6037
cdNV_TGF_B vs. sn_TGF_b	12.68	-14.64 to 40	No	ns	0.9923
cdNV_TGF_B vs. sn_TGF_c	15.41	-11.91 to 42.73	No	ns	0.923
cdNV_TGF_B vs. sn_S80_a	-3.215	-30.54 to 24.11	No	ns	>0.9999
cdNV_TGF_B vs. sn_S80_b	1.412	-25.91 to 28.73	No	ns	>0.9999
cdNV_TGF_B vs. sn_S80_c	7.924	-19.4 to 35.25	No	ns	>0.9999
cdNV_DMEM_B vs. cdNV_S80_C	4.83	-24.38 to 34.04	No	ns	>0.9999
cdNV_DMEM_B vs. cdNV_RolPA_C	-11.71	-39.03 to 15.61	No	ns	0.9976
cdNV_DMEM_B vs. cdNV_TGF_C	-12.49	-39.81 to 14.83	No	ns	0.9938
cdNV_DMEM_B vs. cdNV_DMEM_C	-4.03	-33.24 to 25.18	No	ns	>0.9999
cdNV_DMEM_B vs. sn_DMEM_a	-19.43	-48.64 to 9.778	No	ns	0.7118
cdNV_DMEM_B vs. sn_DMEM_b	-20.26	-49.47 to 8.943	No	ns	0.6311

cdNV_DMEM_B vs. sn_DMEM_c	-8.355	-37.56 to 20.85	No	ns	>0.9999
cdNV_DMEM_B vs. sn_RolPA_a	-12.63	-41.84 to 16.58	No	ns	0.9972
cdNV_DMEM_B vs. sn_RolPA_b	-10.25	-39.46 to 18.96	No	ns	>0.9999
cdNV_DMEM_B vs. sn_RolPA_c	-10.35	-39.55 to 18.86	No	ns	>0.9999
cdNV_DMEM_B vs. sn_TGF_a	-0.7638	-29.97 to 28.44	No	ns	>0.9999
cdNV_DMEM_B vs. sn_TGF_b	-7.3	-36.51 to 21.91	No	ns	>0.9999
cdNV_DMEM_B vs. sn_TGF_c	-4.566	-33.77 to 24.64	No	ns	>0.9999
cdNV_DMEM_B vs. sn_S80_a	-23.19	-52.4 to 6.016	No	ns	0.3488
cdNV_DMEM_B vs. sn_S80_b	-18.56	-47.77 to 10.64	No	ns	0.7881
cdNV_DMEM_B vs. sn_S80_c	-12.05	-41.26 to 17.16	No	ns	0.9987
cdNV_S80_C vs. cdNV_RolPA_C	-16.54	-43.86 to 10.78	No	ns	0.8548
cdNV_S80_C vs. cdNV_TGF_C	-17.32	-44.64 to 10	No	ns	0.7921
cdNV_S80_C vs. cdNV_DMEM_C	-8.86	-38.07 to 20.35	No	ns	>0.9999
cdNV_S80_C vs. sn_DMEM_a	-24.26	-53.47 to 4.948	No	ns	0.2637
cdNV_S80_C vs. sn_DMEM_b	-25.09	-54.3 to 4.113	No	ns	0.2074
cdNV_S80_C vs. sn_DMEM_c	-13.18	-42.39 to 16.02	No	ns	0.9948
cdNV_S80_C vs. sn_RolPA_a	-17.46	-46.67 to 11.75	No	ns	0.8691
cdNV_S80_C vs. sn_RolPA_b	-15.08	-44.29 to 14.13	No	ns	0.9701
cdNV_S80_C vs. sn_RolPA_c	-15.18	-44.38 to 14.03	No	ns	0.9678
cdNV_S80_C vs. sn_TGF_a	-5.594	-34.8 to 23.61	No	ns	>0.9999
cdNV_S80_C vs. sn_TGF_b	-12.13	-41.34 to 17.08	No	ns	0.9985
cdNV_S80_C vs. sn_TGF_c	-9.396	-38.6 to 19.81	No	ns	>0.9999
cdNV_S80_C vs. sn_S80_a	-28.02	-57.23 to 1.186	No	ns	0.0782
cdNV_S80_C vs. sn_S80_b	-23.39	-52.6 to 5.813	No	ns	0.3315
cdNV_S80_C vs. sn_S80_c	-16.88	-46.09 to 12.33	No	ns	0.9029
cdNV_RolPA_C vs. cdNV_TGF_C	-0.7825	-26.08 to 24.51	No	ns	>0.9999
cdNV_RolPA_C vs. cdNV_DMEM_C	7.677	-19.64 to 35	No	ns	>0.9999
cdNV_RolPA_C vs. sn_DMEM_a	-7.723	-35.04 to 19.6	No	ns	>0.9999
cdNV_RolPA_C vs. sn_DMEM_b	-8.558	-35.88 to 18.76	No	ns	>0.9999
cdNV_RolPA_C vs. sn_DMEM_c	3.352	-23.97 to 30.67	No	ns	>0.9999
cdNV_RolPA_C vs. sn_RolPA_a	-0.9253	-28.25 to 26.4	No	ns	>0.9999
cdNV_RolPA_C vs. sn_RolPA_b	1.456	-25.87 to 28.78	No	ns	>0.9999
cdNV_RolPA_C vs. sn_RolPA_c	1.361	-25.96 to 28.68	No	ns	>0.9999
cdNV_RolPA_C vs. sn_TGF_a	10.94	-16.38 to 38.26	No	ns	0.9992
cdNV_RolPA_C vs. sn_TGF_b	4.406	-22.91 to 31.73	No	ns	>0.9999
cdNV_RolPA_C vs. sn_TGF_c	7.141	-20.18 to 34.46	No	ns	>0.9999
cdNV_RolPA_C vs. sn_S80_a	-11.48	-38.81 to 15.84	No	ns	0.9982
cdNV_RolPA_C vs. sn_S80_b	-6.858	-34.18 to 20.46	No	ns	>0.9999
cdNV_RolPA_C vs. sn_S80_c	-0.3456	-27.67 to 26.98	No	ns	>0.9999
cdNV_TGF_C vs. cdNV_DMEM_C	8.459	-18.86 to 35.78	No	ns	>0.9999
cdNV_TGF_C vs. sn_DMEM_a	-6.941	-34.26 to 20.38	No	ns	>0.9999
cdNV_TGF_C vs. sn_DMEM_b	-7.775	-35.1 to 19.55	No	ns	>0.9999
cdNV_TGF_C vs. sn_DMEM_c	4.134	-23.19 to 31.46	No	ns	>0.9999
cdNV_TGF_C vs. sn_RolPA_a	-0.1428	-27.46 to 27.18	No	ns	>0.9999
cdNV_TGF_C vs. sn_RolPA_b	2.238	-25.08 to 29.56	No	ns	>0.9999

cdNV_TGF_C vs. sn_RolPA_c	2.144	-25.18 to 29.46	No	ns	>0.9999
cdNV_TGF_C vs. sn_TGF_a	11.73	-15.6 to 39.05	No	ns	0.9975
cdNV_TGF_C vs. sn_TGF_b	5.189	-22.13 to 32.51	No	ns	>0.9999
cdNV_TGF_C vs. sn_TGF_c	7.924	-19.4 to 35.24	No	ns	>0.9999
cdNV_TGF_C vs. sn_S80_a	-10.7	-38.02 to 16.62	No	ns	0.9994
cdNV_TGF_C vs. sn_S80_b	-6.076	-33.4 to 21.25	No	ns	>0.9999
cdNV_TGF_C vs. sn_S80_c	0.4369	-26.88 to 27.76	No	ns	>0.9999
cdNV_DMEM_C vs. sn_DMEM_a	-15.4	-44.61 to 13.81	No	ns	0.962
cdNV_DMEM_C vs. sn_DMEM_b	-16.23	-45.44 to 12.97	No	ns	0.9334
cdNV_DMEM_C vs. sn_DMEM_c	-4.325	-33.53 to 24.88	No	ns	>0.9999
cdNV_DMEM_C vs. sn_RolPA_a	-8.602	-37.81 to 20.61	No	ns	>0.9999
cdNV_DMEM_C vs. sn_RolPA_b	-6.221	-35.43 to 22.99	No	ns	>0.9999
cdNV_DMEM_C vs. sn_RolPA_c	-6.316	-35.52 to 22.89	No	ns	>0.9999
cdNV_DMEM_C vs. sn_TGF_a	3.266	-25.94 to 32.47	No	ns	>0.9999
cdNV_DMEM_C vs. sn_TGF_b	-3.27	-32.48 to 25.94	No	ns	>0.9999
cdNV_DMEM_C vs. sn_TGF_c	-0.5355	-29.74 to 28.67	No	ns	>0.9999
cdNV_DMEM_C vs. sn_S80_a	-19.16	-48.37 to 10.05	No	ns	0.7364
cdNV_DMEM_C vs. sn_S80_b	-14.53	-43.74 to 14.67	No	ns	0.9808
cdNV_DMEM_C vs. sn_S80_c	-8.022	-37.23 to 21.19	No	ns	>0.9999
sn_DMEM_a vs. sn_DMEM_b	-0.8349	-30.04 to 28.37	No	ns	>0.9999
sn_DMEM_a vs. sn_DMEM_c	11.07	-18.13 to 40.28	No	ns	0.9997
sn_DMEM_a vs. sn_RolPA_a	6.798	-22.41 to 36.01	No	ns	>0.9999
sn_DMEM_a vs. sn_RolPA_b	9.179	-20.03 to 38.39	No	ns	>0.9999
sn_DMEM_a vs. sn_RolPA_c	9.084	-20.12 to 38.29	No	ns	>0.9999
sn_DMEM_a vs. sn_TGF_a	18.67	-10.54 to 47.87	No	ns	0.7797
sn_DMEM_a vs. sn_TGF_b	12.13	-17.08 to 41.34	No	ns	0.9985
sn_DMEM_a vs. sn_TGF_c	14.86	-14.34 to 44.07	No	ns	0.9748
sn_DMEM_a vs. sn_S80_a	-3.762	-32.97 to 25.45	No	ns	>0.9999
sn_DMEM_a vs. sn_S80_b	0.8648	-28.34 to 30.07	No	ns	>0.9999
sn_DMEM_a vs. sn_S80_c	7.377	-21.83 to 36.58	No	ns	>0.9999
sn_DMEM_b vs. sn_DMEM_c	11.91	-17.3 to 41.12	No	ns	0.9989
sn_DMEM_b vs. sn_RolPA_a	7.633	-21.57 to 36.84	No	ns	>0.9999
sn_DMEM_b vs. sn_RolPA_b	10.01	-19.19 to 39.22	No	ns	>0.9999
sn_DMEM_b vs. sn_RolPA_c	9.919	-19.29 to 39.13	No	ns	>0.9999
sn_DMEM_b vs. sn_TGF_a	19.5	-9.707 to 48.71	No	ns	0.7051
sn_DMEM_b vs. sn_TGF_b	12.96	-16.24 to 42.17	No	ns	0.9959
sn_DMEM_b vs. sn_TGF_c	15.7	-13.51 to 44.91	No	ns	0.9531
sn_DMEM_b vs. sn_S80_a	-2.927	-32.13 to 26.28	No	ns	>0.9999
sn_DMEM_b vs. sn_S80_b	1.7	-27.51 to 30.91	No	ns	>0.9999
sn_DMEM_b vs. sn_S80_c	8.212	-21 to 37.42	No	ns	>0.9999
sn_DMEM_c vs. sn_RolPA_a	-4.277	-33.48 to 24.93	No	ns	>0.9999
sn_DMEM_c vs. sn_RolPA_b	-1.896	-31.1 to 27.31	No	ns	>0.9999
sn_DMEM_c vs. sn_RolPA_c	-1.991	-31.2 to 27.22	No	ns	>0.9999
sn_DMEM_c vs. sn_TGF_a	7.591	-21.62 to 36.8	No	ns	>0.9999
sn_DMEM_c vs. sn_TGF_b	1.055	-28.15 to 30.26	No	ns	>0.9999

sn_DMEM_c vs. sn_TGF_c	3.789	-25.42 to 33	No	ns	>0.9999
sn_DMEM_c vs. sn_S80_a	-14.84	-44.04 to 14.37	No	ns	0.9753
sn_DMEM_c vs. sn_S80_b	-10.21	-39.42 to 19	No	ns	>0.9999
sn_DMEM_c vs. sn_S80_c	-3.697	-32.9 to 25.51	No	ns	>0.9999
sn_RolPA_a vs. sn_RolPA_b	2.381	-26.83 to 31.59	No	ns	>0.9999
sn_RolPA_a vs. sn_RolPA_c	2.286	-26.92 to 31.49	No	ns	>0.9999
sn_RolPA_a vs. sn_TGF_a	11.87	-17.34 to 41.08	No	ns	0.999
sn_RolPA_a vs. sn_TGF_b	5.332	-23.88 to 34.54	No	ns	>0.9999
sn_RolPA_a vs. sn_TGF_c	8.066	-21.14 to 37.27	No	ns	>0.9999
sn_RolPA_a vs. sn_S80_a	-10.56	-39.77 to 18.65	No	ns	0.9999
sn_RolPA_a vs. sn_S80_b	-5.933	-35.14 to 23.27	No	ns	>0.9999
sn_RolPA_a vs. sn_S80_c	0.5797	-28.63 to 29.79	No	ns	>0.9999
sn_RolPA_b vs. sn_RolPA_c	-0.09473	-29.3 to 29.11	No	ns	>0.9999
sn_RolPA_b vs. sn_TGF_a	9.487	-19.72 to 38.69	No	ns	>0.9999
sn_RolPA_b vs. sn_TGF_b	2.951	-26.26 to 32.16	No	ns	>0.9999
sn_RolPA_b vs. sn_TGF_c	5.685	-23.52 to 34.89	No	ns	>0.9999
sn_RolPA_b vs. sn_S80_a	-12.94	-42.15 to 16.27	No	ns	0.996
sn_RolPA_b vs. sn_S80_b	-8.314	-37.52 to 20.89	No	ns	>0.9999
sn_RolPA_b vs. sn_S80_c	-1.801	-31.01 to 27.41	No	ns	>0.9999
sn_RolPA_c vs. sn_TGF_a	9.582	-19.63 to 38.79	No	ns	>0.9999
sn_RolPA_c vs. sn_TGF_b	3.045	-26.16 to 32.25	No	ns	>0.9999
sn_RolPA_c vs. sn_TGF_c	5.78	-23.43 to 34.99	No	ns	>0.9999
sn_RolPA_c vs. sn_S80_a	-12.85	-42.05 to 16.36	No	ns	0.9964
sn_RolPA_c vs. sn_S80_b	-8.219	-37.43 to 20.99	No	ns	>0.9999
sn_RolPA_c vs. sn_S80_c	-1.707	-30.91 to 27.5	No	ns	>0.9999
sn_TGF_a vs. sn_TGF_b	-6.536	-35.74 to 22.67	No	ns	>0.9999
sn_TGF_a vs. sn_TGF_c	-3.802	-33.01 to 25.41	No	ns	>0.9999
sn_TGF_a vs. sn_S80_a	-22.43	-51.64 to 6.78	No	ns	0.4175
sn_TGF_a vs. sn_S80_b	-17.8	-47.01 to 11.41	No	ns	0.8464
sn_TGF_a vs. sn_S80_c	-11.29	-40.5 to 17.92	No	ns	0.9996
sn_TGF_b vs. sn_TGF_c	2.735	-26.47 to 31.94	No	ns	>0.9999
sn_TGF_b vs. sn_S80_a	-15.89	-45.1 to 13.32	No	ns	0.9466
sn_TGF_b vs. sn_S80_b	-11.26	-40.47 to 17.94	No	ns	0.9996
sn_TGF_b vs. sn_S80_c	-4.752	-33.96 to 24.46	No	ns	>0.9999
sn_TGF_c vs. sn_S80_a	-18.63	-47.83 to 10.58	No	ns	0.783
sn_TGF_c vs. sn_S80_b	-14	-43.21 to 15.21	No	ns	0.9881
sn_TGF_c vs. sn_S80_c	-7.487	-36.69 to 21.72	No	ns	>0.9999
sn_S80_a vs. sn_S80_b	4.626	-24.58 to 33.83	No	ns	>0.9999
sn_S80_a vs. sn_S80_c	11.14	-18.07 to 40.35	No	ns	0.9996
sn_S80_b vs. sn_S80_c	6.513	-22.69 to 35.72	No	ns	>0.9999

Table S7. Statistical analysis.
Results of one-way ANOVA test with a post-hoc Tukey test of COL1A1 mRNA transcription in Figure 4b and Figure S5.

Number of families	1				
Number of comparisons per family	528				
Alpha	0.05				
**Tukey's multiple comparisons test**	**Mean Diff.**	**95.00% CI of diff.**	**Significant ?**	**Summar y**	**Adjusted P Value**
DMEM vs. RolPA	0.515	-0.009175 to 1.039	No	ns	0.0609
DMEM vs. TGF-ß1	-0.243	-0.7347 to 0.2487	No	ns	0.9851
DMEM vs. S80	0.06833	-0.5059 to 0.6425	No	ns	>0.9999
DMEM vs. PBS	-0.075	-0.5992 to 0.4492	No	ns	>0.9999
DMEM vs. HEPES	-0.08	-0.6042 to 0.4442	No	ns	>0.9999
DMEM vs. cdNV_S80_A	0.2017	-0.3725 to 0.7759	No	ns	>0.9999
DMEM vs. cdNV_RolPA_A	0.2517	-0.3225 to 0.8259	No	ns	0.9974
DMEM vs. cdNV_TGF_A	0.255	-0.3192 to 0.8292	No	ns	0.9968
DMEM vs. cdNV_DMEM_A	0.4583	-0.1159 to 1.033	No	ns	0.3425
DMEM vs. cdNV_S80_B	-0.008333	-0.5825 to 0.5659	No	ns	>0.9999
DMEM vs. cdNV_RolPA_B	0.2517	-0.3225 to 0.8259	No	ns	0.9974
DMEM vs. cdNV_TGF_B	0.2517	-0.3225 to 0.8259	No	ns	0.9974
DMEM vs. cdNV_DMEM_B	-0.1617	-0.7359 to 0.4125	No	ns	>0.9999
DMEM vs. cdNV_S80_C	-0.515	-1.089 to 0.0592	No	ns	0.1481
DMEM vs. cdNV_RolPA_C	-0.015	-0.5892 to 0.5592	No	ns	>0.9999
DMEM vs. cdNV_TGF_C	0.065	-0.5092 to 0.6392	No	ns	>0.9999
DMEM vs. cdNV_DMEM_C	-0.03167	-0.6059 to 0.5425	No	ns	>0.9999
DMEM vs. sn_S80_A	0.2217	-0.3525 to 0.7959	No	ns	0.9997
DMEM vs. sn_S80_B	0.02167	-0.5525 to 0.5959	No	ns	>0.9999
DMEM vs. sn_S80_C	0.015	-0.5592 to 0.5892	No	ns	>0.9999
DMEM vs. sn_RolPA_A	0.06167	-0.5125 to 0.6359	No	ns	>0.9999
DMEM vs. sn_RolPA_B	-0.115	-0.6892 to 0.4592	No	ns	>0.9999
DMEM vs. sn_RolPA_C	0.085	-0.4892 to 0.6592	No	ns	>0.9999
DMEM vs. sn_TGF_A	-0.005	-0.5792 to 0.5692	No	ns	>0.9999
DMEM vs. sn_TGF_B	-0.105	-0.6792 to 0.4692	No	ns	>0.9999
DMEM vs. sn_TGF_C	-0.02167	-0.5959 to 0.5525	No	ns	>0.9999
DMEM vs. sn_DMEM_A	0.1017	-0.4725 to 0.6759	No	ns	>0.9999
DMEM vs. sn_DMEM_B	0.03167	-0.5425 to 0.6059	No	ns	>0.9999
DMEM vs. sn_DMEM_C	-0.1183	-0.6925 to 0.4559	No	ns	>0.9999
DMEM vs. S80_A	0.001667	-0.5725 to 0.5759	No	ns	>0.9999
DMEM vs. S80_B	-0.3383	-0.9125 to 0.2359	No	ns	0.8923
DMEM vs. S80_C	-0.2717	-0.8459 to 0.3025	No	ns	0.9918
RolPA vs. TGF-ß1	-0.758	-1.303 to -0.2133	Yes	***	0.0002
RolPA vs. S80	-0.4467	-1.067 to 0.1735	No	ns	0.5617
RolPA vs. PBS	-0.59	-1.164 to -0.0158	Yes	*	0.0363
RolPA vs. HEPES	-0.595	-1.169 to -0.0208	Yes	*	0.0327
RolPA vs. cdNV_S80_A	-0.3133	-0.9335 to 0.3069	No	ns	0.9801
RolPA vs. cdNV_RolPA_A	-0.2633	-0.8835 to 0.3569	No	ns	0.9984
RolPA vs. cdNV_TGF_A	-0.26	-0.8802 to 0.3602	No	ns	0.9987
RolPA vs. cdNV_DMEM_A	-0.05667	-0.6769 to 0.5635	No	ns	>0.9999
RolPA vs. cdNV_S80_B	-0.5233	-1.144 to 0.09688	No	ns	0.2389
RolPA vs. cdNV_RolPA_B	-0.2633	-0.8835 to 0.3569	No	ns	0.9984
RolPA vs. cdNV_TGF_B	-0.2633	-0.8835 to 0.3569	No	ns	0.9984

RolPA vs. cdNV_DMEM_B	-0.6767	-1.297 to -0.05645	Yes	*	0.0167
RolPA vs. cdNV_S80_C	-1.03	-1.65 to -0.4098	Yes	****	<0.0001
RolPA vs. cdNV_RolPA_C	-0.53	-1.15 to 0.09021	No	ns	0.2179
RolPA vs. cdNV_TGF_C	-0.45	-1.07 to 0.1702	No	ns	0.5457
RolPA vs. cdNV_DMEM_C	-0.5467	-1.167 to 0.07355	No	ns	0.1713
RolPA vs. sn_S80_A	-0.2933	-0.9135 to 0.3269	No	ns	0.9918
RolPA vs. sn_S80_B	-0.4933	-1.114 to 0.1269	No	ns	0.3495
RolPA vs. sn_S80_C	-0.5	-1.12 to 0.1202	No	ns	0.3228
RolPA vs. sn_RolPA_A	-0.4533	-1.074 to 0.1669	No	ns	0.5297
RolPA vs. sn_RolPA_B	-0.63	-1.25 to -0.009788	Yes	*	0.0417
RolPA vs. sn_RolPA_C	-0.43	-1.05 to 0.1902	No	ns	0.6412
RolPA vs. sn_TGF_A	-0.52	-1.14 to 0.1002	No	ns	0.2499
RolPA vs. sn_TGF_B	-0.62	-1.24 to 0.0002118	No	ns	0.0502
RolPA vs. sn_TGF_C	-0.5367	-1.157 to 0.08355	No	ns	0.1983
RolPA vs. sn_DMEM_A	-0.4133	-1.034 to 0.2069	No	ns	0.7177
RolPA vs. sn_DMEM_B	-0.4833	-1.104 to 0.1369	No	ns	0.3918
RolPA vs. sn_DMEM_C	-0.6333	-1.254 to -0.01312	Yes	*	0.0391
RolPA vs. S80_A	-0.5133	-1.134 to 0.1069	No	ns	0.2729
RolPA vs. S80_B	-0.8533	-1.474 to -0.2331	Yes	***	0.0003
RolPA vs. S80_C	-0.7867	-1.407 to -0.1665	Yes	**	0.0015
TGF-ß1 vs. S80	0.3113	-0.2817 to 0.9044	No	ns	0.9681
TGF-ß1 vs. PBS	0.168	-0.3767 to 0.7127	No	ns	>0.9999
TGF-ß1 vs. HEPES	0.163	-0.3817 to 0.7077	No	ns	>0.9999
TGF-ß1 vs. cdNV_S80_A	0.4447	-0.1484 to 1.038	No	ns	0.4744
TGF-ß1 vs. cdNV_RolPA_A	0.4947	-0.09837 to 1.088	No	ns	0.259
TGF-ß1 vs. cdNV_TGF_A	0.498	-0.09504 to 1.091	No	ns	0.2472
TGF-ß1 vs. cdNV_DMEM_A	0.7013	0.1083 to 1.294	Yes	**	0.005
TGF-ß1 vs. cdNV_S80_B	0.2347	-0.3584 to 0.8277	No	ns	0.9995
TGF-ß1 vs. cdNV_RolPA_B	0.4947	-0.09837 to 1.088	No	ns	0.259
TGF-ß1 vs. cdNV_TGF_B	0.4947	-0.09837 to 1.088	No	ns	0.259
TGF-ß1 vs. cdNV_DMEM_B	0.08133	-0.5117 to 0.6744	No	ns	>0.9999
TGF-ß1 vs. cdNV_S80_C	-0.272	-0.865 to 0.321	No	ns	0.9948
TGF-ß1 vs. cdNV_RolPA_C	0.228	-0.365 to 0.821	No	ns	0.9997
TGF-ß1 vs. cdNV_TGF_C	0.308	-0.285 to 0.901	No	ns	0.9719
TGF-ß1 vs. cdNV_DMEM_C	0.2113	-0.3817 to 0.8044	No	ns	>0.9999
TGF-ß1 vs. sn_S80_A	0.4647	-0.1284 to 1.058	No	ns	0.3804
TGF-ß1 vs. sn_S80_B	0.2647	-0.3284 to 0.8577	No	ns	0.9965
TGF-ß1 vs. sn_S80_C	0.258	-0.335 to 0.851	No	ns	0.9977
TGF-ß1 vs. sn_RolPA_A	0.3047	-0.2884 to 0.8977	No	ns	0.9754
TGF-ß1 vs. sn_RolPA_B	0.128	-0.465 to 0.721	No	ns	>0.9999
TGF-ß1 vs. sn_RolPA_C	0.328	-0.265 to 0.921	No	ns	0.9426
TGF-ß1 vs. sn_TGF_A	0.238	-0.355 to 0.831	No	ns	0.9994
TGF-ß1 vs. sn_TGF_B	0.138	-0.455 to 0.731	No	ns	>0.9999
TGF-ß1 vs. sn_TGF_C	0.2213	-0.3717 to 0.8144	No	ns	0.9998
TGF-ß1 vs. sn_DMEM_A	0.3447	-0.2484 to 0.9377	No	ns	0.9053
TGF-ß1 vs. sn_DMEM_B	0.2747	-0.3184 to 0.8677	No	ns	0.994
TGF-ß1 vs. sn_DMEM_C	0.1247	-0.4684 to 0.7177	No	ns	>0.9999
TGF-ß1 vs. S80_A	0.2447	-0.3484 to 0.8377	No	ns	0.999
TGF-ß1 vs. S80_B	-0.09533	-0.6884 to 0.4977	No	ns	>0.9999
TGF-ß1 vs. S80_C	-0.02867	-0.6217 to 0.5644	No	ns	>0.9999
S80 vs. PBS	-0.1433	-0.7635 to 0.4769	No	ns	>0.9999
S80 vs. HEPES	-0.1483	-0.7685 to 0.4719	No	ns	>0.9999
S80 vs. cdNV_S80_A	0.1333	-0.5297 to 0.7964	No	ns	>0.9999
S80 vs. cdNV_RolPA_A	0.1833	-0.4797 to 0.8464	No	ns	>0.9999

S80 vs. cdNV_TGF_A	0.1867	-0.4764 to 0.8497	No	ns	>0.9999
S80 vs. cdNV_DMEM_A	0.39	-0.273 to 1.053	No	ns	0.894
S80 vs. cdNV_S80_B	-0.07667	-0.7397 to 0.5864	No	ns	>0.9999
S80 vs. cdNV_RolPA_B	0.1833	-0.4797 to 0.8464	No	ns	>0.9999
S80 vs. cdNV_TGF_B	0.1833	-0.4797 to 0.8464	No	ns	>0.9999
S80 vs. cdNV_DMEM_B	-0.23	-0.893 to 0.433	No	ns	>0.9999
S80 vs. cdNV_S80_C	-0.5833	-1.246 to 0.0797	No	ns	0.1739
S80 vs. cdNV_RolPA_C	-0.08333	-0.7464 to 0.5797	No	ns	>0.9999
S80 vs. cdNV_TGF_C	-0.003333	-0.6664 to 0.6597	No	ns	>0.9999
S80 vs. cdNV_DMEM_C	-0.1	-0.763 to 0.563	No	ns	>0.9999
S80 vs. sn_S80_A	0.1533	-0.5097 to 0.8164	No	ns	>0.9999
S80 vs. sn_S80_B	-0.04667	-0.7097 to 0.6164	No	ns	>0.9999
S80 vs. sn_S80_C	-0.05333	-0.7164 to 0.6097	No	ns	>0.9999
S80 vs. sn_RolPA_A	-0.006667	-0.6697 to 0.6564	No	ns	>0.9999
S80 vs. sn_RolPA_B	-0.1833	-0.8464 to 0.4797	No	ns	>0.9999
S80 vs. sn_RolPA_C	0.01667	-0.6464 to 0.6797	No	ns	>0.9999
S80 vs. sn_TGF_A	-0.07333	-0.7364 to 0.5897	No	ns	>0.9999
S80 vs. sn_TGF_B	-0.1733	-0.8364 to 0.4897	No	ns	>0.9999
S80 vs. sn_TGF_C	-0.09	-0.753 to 0.573	No	ns	>0.9999
S80 vs. sn_DMEM_A	0.03333	-0.6297 to 0.6964	No	ns	>0.9999
S80 vs. sn_DMEM_B	-0.03667	-0.6997 to 0.6264	No	ns	>0.9999
S80 vs. sn_DMEM_C	-0.1867	-0.8497 to 0.4764	No	ns	>0.9999
S80 vs. S80_A	-0.06667	-0.7297 to 0.5964	No	ns	>0.9999
S80 vs. S80_B	-0.4067	-1.07 to 0.2564	No	ns	0.847
S80 vs. S80_C	-0.34	-1.003 to 0.323	No	ns	0.976
PBS vs. HEPES	-0.005	-0.5792 to 0.5692	No	ns	>0.9999
PBS vs. cdNV_S80_A	0.2767	-0.3435 to 0.8969	No	ns	0.9966
PBS vs. cdNV_RolPA_A	0.3267	-0.2935 to 0.9469	No	ns	0.9668
PBS vs. cdNV_TGF_A	0.33	-0.2902 to 0.9502	No	ns	0.9627
PBS vs. cdNV_DMEM_A	0.5333	-0.08688 to 1.154	No	ns	0.208
PBS vs. cdNV_S80_B	0.06667	-0.5535 to 0.6869	No	ns	>0.9999
PBS vs. cdNV_RolPA_B	0.3267	-0.2935 to 0.9469	No	ns	0.9668
PBS vs. cdNV_TGF_B	0.3267	-0.2935 to 0.9469	No	ns	0.9668
PBS vs. cdNV_DMEM_B	-0.08667	-0.7069 to 0.5335	No	ns	>0.9999
PBS vs. cdNV_S80_C	-0.44	-1.06 to 0.1802	No	ns	0.5936
PBS vs. cdNV_RolPA_C	0.06	-0.5602 to 0.6802	No	ns	>0.9999
PBS vs. cdNV_TGF_C	0.14	-0.4802 to 0.7602	No	ns	>0.9999
PBS vs. cdNV_DMEM_C	0.04333	-0.5769 to 0.6635	No	ns	>0.9999
PBS vs. sn_S80_A	0.2967	-0.3235 to 0.9169	No	ns	0.9904
PBS vs. sn_S80_B	0.09667	-0.5235 to 0.7169	No	ns	>0.9999
PBS vs. sn_S80_C	0.09	-0.5302 to 0.7102	No	ns	>0.9999
PBS vs. sn_RolPA_A	0.1367	-0.4835 to 0.7569	No	ns	>0.9999
PBS vs. sn_RolPA_B	-0.04	-0.6602 to 0.5802	No	ns	>0.9999
PBS vs. sn_RolPA_C	0.16	-0.4602 to 0.7802	No	ns	>0.9999
PBS vs. sn_TGF_A	0.07	-0.5502 to 0.6902	No	ns	>0.9999
PBS vs. sn_TGF_B	-0.03	-0.6502 to 0.5902	No	ns	>0.9999
PBS vs. sn_TGF_C	0.05333	-0.5669 to 0.6735	No	ns	>0.9999
PBS vs. sn_DMEM_A	0.1767	-0.4435 to 0.7969	No	ns	>0.9999
PBS vs. sn_DMEM_B	0.1067	-0.5135 to 0.7269	No	ns	>0.9999
PBS vs. sn_DMEM_C	-0.04333	-0.6635 to 0.5769	No	ns	>0.9999
PBS vs. S80_A	0.07667	-0.5435 to 0.6969	No	ns	>0.9999
PBS vs. S80_B	-0.2633	-0.8835 to 0.3569	No	ns	0.9984
PBS vs. S80_C	-0.1967	-0.8169 to 0.4235	No	ns	>0.9999
HEPES vs. cdNV_S80_A	0.2817	-0.3385 to 0.9019	No	ns	0.9955
HEPES vs. cdNV_RolPA_A	0.3317	-0.2885 to 0.9519	No	ns	0.9604
HEPES vs. cdNV_TGF_A	0.335	-0.2852 to 0.9552	No	ns	0.9557

HEPES vs. cdNV_DMEM_A	0.5383	-0.08188 to 1.159	No	ns	0.1936
HEPES vs. cdNV_S80_B	0.07167	-0.5485 to 0.6919	No	ns	>0.9999
HEPES vs. cdNV_RolPA_B	0.3317	-0.2885 to 0.9519	No	ns	0.9604
HEPES vs. cdNV_TGF_B	0.3317	-0.2885 to 0.9519	No	ns	0.9604
HEPES vs. cdNV_DMEM_B	-0.08167	-0.7019 to 0.5385	No	ns	>0.9999
HEPES vs. cdNV_S80_C	-0.435	-1.055 to 0.1852	No	ns	0.6175
HEPES vs. cdNV_RolPA_C	0.065	-0.5552 to 0.6852	No	ns	>0.9999
HEPES vs. cdNV_TGF_C	0.145	-0.4752 to 0.7652	No	ns	>0.9999
HEPES vs. cdNV_DMEM_C	0.04833	-0.5719 to 0.6685	No	ns	>0.9999
HEPES vs. sn_S80_A	0.3017	-0.3185 to 0.9219	No	ns	0.9879
HEPES vs. sn_S80_B	0.1017	-0.5185 to 0.7219	No	ns	>0.9999
HEPES vs. sn_S80_C	0.095	-0.5252 to 0.7152	No	ns	>0.9999
HEPES vs. sn_RolPA_A	0.1417	-0.4785 to 0.7619	No	ns	>0.9999
HEPES vs. sn_RolPA_B	-0.035	-0.6552 to 0.5852	No	ns	>0.9999
HEPES vs. sn_RolPA_C	0.165	-0.4552 to 0.7852	No	ns	>0.9999
HEPES vs. sn_TGF_A	0.075	-0.5452 to 0.6952	No	ns	>0.9999
HEPES vs. sn_TGF_B	-0.025	-0.6452 to 0.5952	No	ns	>0.9999
HEPES vs. sn_TGF_C	0.05833	-0.5619 to 0.6785	No	ns	>0.9999
HEPES vs. sn_DMEM_A	0.1817	-0.4385 to 0.8019	No	ns	>0.9999
HEPES vs. sn_DMEM_B	0.1117	-0.5085 to 0.7319	No	ns	>0.9999
HEPES vs. sn_DMEM_C	-0.03833	-0.6585 to 0.5819	No	ns	>0.9999
HEPES vs. S80_A	0.08167	-0.5385 to 0.7019	No	ns	>0.9999
HEPES vs. S80_B	-0.2583	-0.8785 to 0.3619	No	ns	0.9989
HEPES vs. S80_C	-0.1917	-0.8119 to 0.4285	No	ns	>0.9999
cdNV_S80_A vs. cdNV_RolPA_A	0.05	-0.613 to 0.713	No	ns	>0.9999
cdNV_S80_A vs. cdNV_TGF_A	0.05333	-0.6097 to 0.7164	No	ns	>0.9999
cdNV_S80_A vs. cdNV_DMEM_A	0.2567	-0.4064 to 0.9197	No	ns	0.9997
cdNV_S80_A vs. cdNV_S80_B	-0.21	-0.873 to 0.453	No	ns	>0.9999
cdNV_S80_A vs. cdNV_RolPA_B	0.05	-0.613 to 0.713	No	ns	>0.9999
cdNV_S80_A vs. cdNV_TGF_B	0.05	-0.613 to 0.713	No	ns	>0.9999
cdNV_S80_A vs. cdNV_DMEM_B	-0.3633	-1.026 to 0.2997	No	ns	0.948
cdNV_S80_A vs. cdNV_S80_C	-0.7167	-1.38 to -0.05363	Yes	*	0.0189
cdNV_S80_A vs. cdNV_RolPA_C	-0.2167	-0.8797 to 0.4464	No	ns	>0.9999
cdNV_S80_A vs. cdNV_TGF_C	-0.1367	-0.7997 to 0.5264	No	ns	>0.9999
cdNV_S80_A vs. cdNV_DMEM_C	-0.2333	-0.8964 to 0.4297	No	ns	>0.9999
cdNV_S80_A vs. sn_S80_A	0.02	-0.643 to 0.683	No	ns	>0.9999
cdNV_S80_A vs. sn_S80_B	-0.18	-0.843 to 0.483	No	ns	>0.9999
cdNV_S80_A vs. sn_S80_C	-0.1867	-0.8497 to 0.4764	No	ns	>0.9999
cdNV_S80_A vs. sn_RolPA_A	-0.14	-0.803 to 0.523	No	ns	>0.9999
cdNV_S80_A vs. sn_RolPA_B	-0.3167	-0.9797 to 0.3464	No	ns	0.9906
cdNV_S80_A vs. sn_RolPA_C	-0.1167	-0.7797 to 0.5464	No	ns	>0.9999
cdNV_S80_A vs. sn_TGF_A	-0.2067	-0.8697 to 0.4564	No	ns	>0.9999
cdNV_S80_A vs. sn_TGF_B	-0.3067	-0.9697 to 0.3564	No	ns	0.9941
cdNV_S80_A vs. sn_TGF_C	-0.2233	-0.8864 to 0.4397	No	ns	>0.9999
cdNV_S80_A vs. sn_DMEM_A	-0.1	-0.763 to 0.563	No	ns	>0.9999
cdNV_S80_A vs. sn_DMEM_B	-0.17	-0.833 to 0.493	No	ns	>0.9999
cdNV_S80_A vs. sn_DMEM_C	-0.32	-0.983 to 0.343	No	ns	0.9891
cdNV_S80_A vs. S80_A	-0.2	-0.863 to 0.463	No	ns	>0.9999
cdNV_S80_A vs. S80_B	-0.54	-1.203 to 0.123	No	ns	0.303
cdNV_S80_A vs. S80_C	-0.4733	-1.136 to 0.1897	No	ns	0.5804
cdNV_RolPA_A vs. cdNV_TGF_A	0.003333	-0.6597 to 0.6664	No	ns	>0.9999
cdNV_RolPA_A vs. cdNV_DMEM_A	0.2067	-0.4564 to 0.8697	No	ns	>0.9999
cdNV_RolPA_A vs. cdNV_S80_B	-0.26	-0.923 to 0.403	No	ns	0.9996
cdNV_RolPA_A vs. cdNV_RolPA_B	0	-0.663 to 0.663	No	ns	>0.9999
cdNV_RolPA_A vs. cdNV_TGF_B	0	-0.663 to 0.663	No	ns	>0.9999
cdNV_RolPA_A vs. cdNV_DMEM_B	-0.4133	-1.076 to 0.2497	No	ns	0.8254

cdNV_RolPA_A vs. cdNV_S80_C	-0.7667	-1.43 to -0.1036	Yes	**	0.0071
cdNV_RolPA_A vs. cdNV_RolPA_C	-0.2667	-0.9297 to 0.3964	No	ns	0.9994
cdNV_RolPA_A vs. cdNV_TGF_C	-0.1867	-0.8497 to 0.4764	No	ns	>0.9999
cdNV_RolPA_A vs. cdNV_DMEM_C	-0.2833	-0.9464 to 0.3797	No	ns	0.9983
cdNV_RolPA_A vs. sn_S80_A	-0.03	-0.693 to 0.633	No	ns	>0.9999
cdNV_RolPA_A vs. sn_S80_B	-0.23	-0.893 to 0.433	No	ns	>0.9999
cdNV_RolPA_A vs. sn_S80_C	-0.2367	-0.8997 to 0.4264	No	ns	>0.9999
cdNV_RolPA_A vs. sn_RolPA_A	-0.19	-0.853 to 0.473	No	ns	>0.9999
cdNV_RolPA_A vs. sn_RolPA_B	-0.3667	-1.03 to 0.2964	No	ns	0.9427
cdNV_RolPA_A vs. sn_RolPA_C	-0.1667	-0.8297 to 0.4964	No	ns	>0.9999
cdNV_RolPA_A vs. sn_TGF_A	-0.2567	-0.9197 to 0.4064	No	ns	0.9997
cdNV_RolPA_A vs. sn_TGF_B	-0.3567	-1.02 to 0.3064	No	ns	0.9577
cdNV_RolPA_A vs. sn_TGF_C	-0.2733	-0.9364 to 0.3897	No	ns	0.9991
cdNV_RolPA_A vs. sn_DMEM_A	-0.15	-0.813 to 0.513	No	ns	>0.9999
cdNV_RolPA_A vs. sn_DMEM_B	-0.22	-0.883 to 0.443	No	ns	>0.9999
cdNV_RolPA_A vs. sn_DMEM_C	-0.37	-1.033 to 0.293	No	ns	0.9369
cdNV_RolPA_A vs. S80_A	-0.25	-0.913 to 0.413	No	ns	0.9998
cdNV_RolPA_A vs. S80_B	-0.59	-1.253 to 0.07303	No	ns	0.1583
cdNV_RolPA_A vs. S80_C	-0.5233	-1.186 to 0.1397	No	ns	0.3653
cdNV_TGF_A vs. cdNV_DMEM_A	0.2033	-0.4597 to 0.8664	No	ns	>0.9999
cdNV_TGF_A vs. cdNV_S80_B	-0.2633	-0.9264 to 0.3997	No	ns	0.9995
cdNV_TGF_A vs. cdNV_RolPA_B	-0.003333	-0.6664 to 0.6597	No	ns	>0.9999
cdNV_TGF_A vs. cdNV_TGF_B	-0.003333	-0.6664 to 0.6597	No	ns	>0.9999
cdNV_TGF_A vs. cdNV_DMEM_B	-0.4167	-1.08 to 0.2464	No	ns	0.8141
cdNV_TGF_A vs. cdNV_S80_C	-0.77	-1.433 to -0.107	Yes	**	0.0066
cdNV_TGF_A vs. cdNV_RolPA_C	-0.27	-0.933 to 0.393	No	ns	0.9992
cdNV_TGF_A vs. cdNV_TGF_C	-0.19	-0.853 to 0.473	No	ns	>0.9999
cdNV_TGF_A vs. cdNV_DMEM_C	-0.2867	-0.9497 to 0.3764	No	ns	0.9979
cdNV_TGF_A vs. sn_S80_A	-0.03333	-0.6964 to 0.6297	No	ns	>0.9999
cdNV_TGF_A vs. sn_S80_B	-0.2333	-0.8964 to 0.4297	No	ns	>0.9999
cdNV_TGF_A vs. sn_S80_C	-0.24	-0.903 to 0.423	No	ns	>0.9999
cdNV_TGF_A vs. sn_RolPA_A	-0.1933	-0.8564 to 0.4697	No	ns	>0.9999
cdNV_TGF_A vs. sn_RolPA_B	-0.37	-1.033 to 0.293	No	ns	0.9369
cdNV_TGF_A vs. sn_RolPA_C	-0.17	-0.833 to 0.493	No	ns	>0.9999
cdNV_TGF_A vs. sn_TGF_A	-0.26	-0.923 to 0.403	No	ns	0.9996
cdNV_TGF_A vs. sn_TGF_B	-0.36	-1.023 to 0.303	No	ns	0.953
cdNV_TGF_A vs. sn_TGF_C	-0.2767	-0.9397 to 0.3864	No	ns	0.9988
cdNV_TGF_A vs. sn_DMEM_A	-0.1533	-0.8164 to 0.5097	No	ns	>0.9999
cdNV_TGF_A vs. sn_DMEM_B	-0.2233	-0.8864 to 0.4397	No	ns	>0.9999
cdNV_TGF_A vs. sn_DMEM_C	-0.3733	-1.036 to 0.2897	No	ns	0.9308
cdNV_TGF_A vs. S80_A	-0.2533	-0.9164 to 0.4097	No	ns	0.9998
cdNV_TGF_A vs. S80_B	-0.5933	-1.256 to 0.0697	No	ns	0.1509
cdNV_TGF_A vs. S80_C	-0.5267	-1.19 to 0.1364	No	ns	0.3523
cdNV_DMEM_A vs. cdNV_S80_B	-0.4667	-1.13 to 0.1964	No	ns	0.6102
cdNV_DMEM_A vs. cdNV_RolPA_B	-0.2067	-0.8697 to 0.4564	No	ns	>0.9999
cdNV_DMEM_A vs. cdNV_TGF_B	-0.2067	-0.8697 to 0.4564	No	ns	>0.9999
cdNV_DMEM_A vs. cdNV_DMEM_B	-0.62	-1.283 to 0.04303	No	ns	0.1013
cdNV_DMEM_A vs. cdNV_S80_C	-0.9733	-1.636 to -0.3103	Yes	****	<0.0001
cdNV_DMEM_A vs. cdNV_RolPA_C	-0.4733	-1.136 to 0.1897	No	ns	0.5804
cdNV_DMEM_A vs. cdNV_TGF_C	-0.3933	-1.056 to 0.2697	No	ns	0.8855
cdNV_DMEM_A vs. cdNV_DMEM_C	-0.49	-1.153 to 0.173	No	ns	0.5058
cdNV_DMEM_A vs. sn_S80_A	-0.2367	-0.8997 to 0.4264	No	ns	>0.9999
cdNV_DMEM_A vs. sn_S80_B	-0.4367	-1.1 to 0.2264	No	ns	0.7389
cdNV_DMEM_A vs. sn_S80_C	-0.4433	-1.106 to 0.2197	No	ns	0.7116
cdNV_DMEM_A vs. sn_RolPA_A	-0.3967	-1.06 to 0.2664	No	ns	0.8765
cdNV_DMEM_A vs. sn_RolPA_B	-0.5733	-1.236 to 0.0897	No	ns	0.1993

cdNV_DMEM_A vs. sn_RolPA_C	-0.3733	-1.036 to 0.2897	No	ns	0.9308
cdNV_DMEM_A vs. sn_TGF_A	-0.4633	-1.126 to 0.1997	No	ns	0.6251
cdNV_DMEM_A vs. sn_TGF_B	-0.5633	-1.226 to 0.0997	No	ns	0.2274
cdNV_DMEM_A vs. sn_TGF_C	-0.48	-1.143 to 0.183	No	ns	0.5505
cdNV_DMEM_A vs. sn_DMEM_A	-0.3567	-1.02 to 0.3064	No	ns	0.9577
cdNV_DMEM_A vs. sn_DMEM_B	-0.4267	-1.09 to 0.2364	No	ns	0.7779
cdNV_DMEM_A vs. sn_DMEM_C	-0.5767	-1.24 to 0.08637	No	ns	0.1906
cdNV_DMEM_A vs. S80_A	-0.4567	-1.12 to 0.2064	No	ns	0.6545
cdNV_DMEM_A vs. S80_B	-0.7967	-1.46 to -0.1336	Yes	**	0.0038
cdNV_DMEM_A vs. S80_C	-0.73	-1.393 to -0.06697	Yes	*	0.0147
cdNV_S80_B vs. cdNV_RolPA_B	0.26	-0.403 to 0.923	No	ns	0.9996
cdNV_S80_B vs. cdNV_TGF_B	0.26	-0.403 to 0.923	No	ns	0.9996
cdNV_S80_B vs. cdNV_DMEM_B	-0.1533	-0.8164 to 0.5097	No	ns	>0.9999
cdNV_S80_B vs. cdNV_S80_C	-0.5067	-1.17 to 0.1564	No	ns	0.4334
cdNV_S80_B vs. cdNV_RolPA_C	-0.006667	-0.6697 to 0.6564	No	ns	>0.9999
cdNV_S80_B vs. cdNV_TGF_C	0.07333	-0.5897 to 0.7364	No	ns	>0.9999
cdNV_S80_B vs. cdNV_DMEM_C	-0.02333	-0.6864 to 0.6397	No	ns	>0.9999
cdNV_S80_B vs. sn_S80_A	0.23	-0.433 to 0.893	No	ns	>0.9999
cdNV_S80_B vs. sn_S80_B	0.03	-0.633 to 0.693	No	ns	>0.9999
cdNV_S80_B vs. sn_S80_C	0.02333	-0.6397 to 0.6864	No	ns	>0.9999
cdNV_S80_B vs. sn_RolPA_A	0.07	-0.593 to 0.733	No	ns	>0.9999
cdNV_S80_B vs. sn_RolPA_B	-0.1067	-0.7697 to 0.5564	No	ns	>0.9999
cdNV_S80_B vs. sn_RolPA_C	0.09333	-0.5697 to 0.7564	No	ns	>0.9999
cdNV_S80_B vs. sn_TGF_A	0.003333	-0.6597 to 0.6664	No	ns	>0.9999
cdNV_S80_B vs. sn_TGF_B	-0.09667	-0.7597 to 0.5664	No	ns	>0.9999
cdNV_S80_B vs. sn_TGF_C	-0.01333	-0.6764 to 0.6497	No	ns	>0.9999
cdNV_S80_B vs. sn_DMEM_A	0.11	-0.553 to 0.773	No	ns	>0.9999
cdNV_S80_B vs. sn_DMEM_B	0.04	-0.623 to 0.703	No	ns	>0.9999
cdNV_S80_B vs. sn_DMEM_C	-0.11	-0.773 to 0.553	No	ns	>0.9999
cdNV_S80_B vs. S80_A	0.01	-0.653 to 0.673	No	ns	>0.9999
cdNV_S80_B vs. S80_B	-0.33	-0.993 to 0.333	No	ns	0.9836
cdNV_S80_B vs. S80_C	-0.2633	-0.9264 to 0.3997	No	ns	0.9995
cdNV_RolPA_B vs. cdNV_TGF_B	0	-0.663 to 0.663	No	ns	>0.9999
cdNV_RolPA_B vs. cdNV_DMEM_B	-0.4133	-1.076 to 0.2497	No	ns	0.8254
cdNV_RolPA_B vs. cdNV_S80_C	-0.7667	-1.43 to -0.1036	Yes	**	0.0071
cdNV_RolPA_B vs. cdNV_RolPA_C	-0.2667	-0.9297 to 0.3964	No	ns	0.9994
cdNV_RolPA_B vs. cdNV_TGF_C	-0.1867	-0.8497 to 0.4764	No	ns	>0.9999
cdNV_RolPA_B vs. cdNV_DMEM_C	-0.2833	-0.9464 to 0.3797	No	ns	0.9983
cdNV_RolPA_B vs. sn_S80_A	-0.03	-0.693 to 0.633	No	ns	>0.9999
cdNV_RolPA_B vs. sn_S80_B	-0.23	-0.893 to 0.433	No	ns	>0.9999
cdNV_RolPA_B vs. sn_S80_C	-0.2367	-0.8997 to 0.4264	No	ns	>0.9999
cdNV_RolPA_B vs. sn_RolPA_A	-0.19	-0.853 to 0.473	No	ns	>0.9999
cdNV_RolPA_B vs. sn_RolPA_B	-0.3667	-1.03 to 0.2964	No	ns	0.9427
cdNV_RolPA_B vs. sn_RolPA_C	-0.1667	-0.8297 to 0.4964	No	ns	>0.9999
cdNV_RolPA_B vs. sn_TGF_A	-0.2567	-0.9197 to 0.4064	No	ns	0.9997
cdNV_RolPA_B vs. sn_TGF_B	-0.3567	-1.02 to 0.3064	No	ns	0.9577
cdNV_RolPA_B vs. sn_TGF_C	-0.2733	-0.9364 to 0.3897	No	ns	0.9991
cdNV_RolPA_B vs. sn_DMEM_A	-0.15	-0.813 to 0.513	No	ns	>0.9999
cdNV_RolPA_B vs. sn_DMEM_B	-0.22	-0.883 to 0.443	No	ns	>0.9999
cdNV_RolPA_B vs. sn_DMEM_C	-0.37	-1.033 to 0.293	No	ns	0.9369
cdNV_RolPA_B vs. S80_A	-0.25	-0.913 to 0.413	No	ns	0.9998
cdNV_RolPA_B vs. S80_B	-0.59	-1.253 to 0.07303	No	ns	0.1583
cdNV_RolPA_B vs. S80_C	-0.5233	-1.186 to 0.1397	No	ns	0.3653
cdNV_TGF_B vs. cdNV_DMEM_B	-0.4133	-1.076 to 0.2497	No	ns	0.8254
cdNV_TGF_B vs. cdNV_S80_C	-0.7667	-1.43 to -0.1036	Yes	**	0.0071
cdNV_TGF_B vs. cdNV_RolPA_C	-0.2667	-0.9297 to 0.3964	No	ns	0.9994

cdNV_TGF_B vs. cdNV_TGF_C	-0.1867	-0.8497 to 0.4764	No	ns	>0.9999
cdNV_TGF_B vs. cdNV_DMEM_C	-0.2833	-0.9464 to 0.3797	No	ns	0.9983
cdNV_TGF_B vs. sn_S80_A	-0.03	-0.693 to 0.633	No	ns	>0.9999
cdNV_TGF_B vs. sn_S80_B	-0.23	-0.893 to 0.433	No	ns	>0.9999
cdNV_TGF_B vs. sn_S80_C	-0.2367	-0.8997 to 0.4264	No	ns	>0.9999
cdNV_TGF_B vs. sn_RolPA_A	-0.19	-0.853 to 0.473	No	ns	>0.9999
cdNV_TGF_B vs. sn_RolPA_B	-0.3667	-1.03 to 0.2964	No	ns	0.9427
cdNV_TGF_B vs. sn_RolPA_C	-0.1667	-0.8297 to 0.4964	No	ns	>0.9999
cdNV_TGF_B vs. sn_TGF_A	-0.2567	-0.9197 to 0.4064	No	ns	0.9997
cdNV_TGF_B vs. sn_TGF_B	-0.3567	-1.02 to 0.3064	No	ns	0.9577
cdNV_TGF_B vs. sn_TGF_C	-0.2733	-0.9364 to 0.3897	No	ns	0.9991
cdNV_TGF_B vs. sn_DMEM_A	-0.15	-0.813 to 0.513	No	ns	>0.9999
cdNV_TGF_B vs. sn_DMEM_B	-0.22	-0.883 to 0.443	No	ns	>0.9999
cdNV_TGF_B vs. sn_DMEM_C	-0.37	-1.033 to 0.293	No	ns	0.9369
cdNV_TGF_B vs. S80_A	-0.25	-0.913 to 0.413	No	ns	0.9998
cdNV_TGF_B vs. S80_B	-0.59	-1.253 to 0.07303	No	ns	0.1583
cdNV_TGF_B vs. S80_C	-0.5233	-1.186 to 0.1397	No	ns	0.3653
cdNV_DMEM_B vs. cdNV_S80_C	-0.3533	-1.016 to 0.3097	No	ns	0.962
cdNV_DMEM_B vs. cdNV_RolPA_C	0.1467	-0.5164 to 0.8097	No	ns	>0.9999
cdNV_DMEM_B vs. cdNV_TGF_C	0.2267	-0.4364 to 0.8897	No	ns	>0.9999
cdNV_DMEM_B vs. cdNV_DMEM_C	0.13	-0.533 to 0.793	No	ns	>0.9999
cdNV_DMEM_B vs. sn_S80_A	0.3833	-0.2797 to 1.046	No	ns	0.91
cdNV_DMEM_B vs. sn_S80_B	0.1833	-0.4797 to 0.8464	No	ns	>0.9999
cdNV_DMEM_B vs. sn_S80_C	0.1767	-0.4864 to 0.8397	No	ns	>0.9999
cdNV_DMEM_B vs. sn_RolPA_A	0.2233	-0.4397 to 0.8864	No	ns	>0.9999
cdNV_DMEM_B vs. sn_RolPA_B	0.04667	-0.6164 to 0.7097	No	ns	>0.9999
cdNV_DMEM_B vs. sn_RolPA_C	0.2467	-0.4164 to 0.9097	No	ns	0.9999
cdNV_DMEM_B vs. sn_TGF_A	0.1567	-0.5064 to 0.8197	No	ns	>0.9999
cdNV_DMEM_B vs. sn_TGF_B	0.05667	-0.6064 to 0.7197	No	ns	>0.9999
cdNV_DMEM_B vs. sn_TGF_C	0.14	-0.523 to 0.803	No	ns	>0.9999
cdNV_DMEM_B vs. sn_DMEM_A	0.2633	-0.3997 to 0.9264	No	ns	0.9995
cdNV_DMEM_B vs. sn_DMEM_B	0.1933	-0.4697 to 0.8564	No	ns	>0.9999
cdNV_DMEM_B vs. sn_DMEM_C	0.04333	-0.6197 to 0.7064	No	ns	>0.9999
cdNV_DMEM_B vs. S80_A	0.1633	-0.4997 to 0.8264	No	ns	>0.9999
cdNV_DMEM_B vs. S80_B	-0.1767	-0.8397 to 0.4864	No	ns	>0.9999
cdNV_DMEM_B vs. S80_C	-0.11	-0.773 to 0.553	No	ns	>0.9999
cdNV_S80_C vs. cdNV_RolPA_C	0.5	-0.163 to 1.163	No	ns	0.462
cdNV_S80_C vs. cdNV_TGF_C	0.58	-0.08303 to 1.243	No	ns	0.1821
cdNV_S80_C vs. cdNV_DMEM_C	0.4833	-0.1797 to 1.146	No	ns	0.5355
cdNV_S80_C vs. sn_S80_A	0.7367	0.07363 to 1.4	Yes	*	0.0129
cdNV_S80_C vs. sn_S80_B	0.5367	-0.1264 to 1.2	No	ns	0.3149
cdNV_S80_C vs. sn_S80_C	0.53	-0.133 to 1.193	No	ns	0.3396
cdNV_S80_C vs. sn_RolPA_A	0.5767	-0.08637 to 1.24	No	ns	0.1906
cdNV_S80_C vs. sn_RolPA_B	0.4	-0.263 to 1.063	No	ns	0.8671
cdNV_S80_C vs. sn_RolPA_C	0.6	-0.06303 to 1.263	No	ns	0.137
cdNV_S80_C vs. sn_TGF_A	0.51	-0.153 to 1.173	No	ns	0.4194
cdNV_S80_C vs. sn_TGF_B	0.41	-0.253 to 1.073	No	ns	0.8364
cdNV_S80_C vs. sn_TGF_C	0.4933	-0.1697 to 1.156	No	ns	0.4911
cdNV_S80_C vs. sn_DMEM_A	0.6167	-0.04637 to 1.28	No	ns	0.1066
cdNV_S80_C vs. sn_DMEM_B	0.5467	-0.1164 to 1.21	No	ns	0.28
cdNV_S80_C vs. sn_DMEM_C	0.3967	-0.2664 to 1.06	No	ns	0.8765
cdNV_S80_C vs. S80_A	0.5167	-0.1464 to 1.18	No	ns	0.3919
cdNV_S80_C vs. S80_B	0.1767	-0.4864 to 0.8397	No	ns	>0.9999
cdNV_S80_C vs. S80_C	0.2433	-0.4197 to 0.9064	No	ns	0.9999
cdNV_RolPA_C vs. cdNV_TGF_C	0.08	-0.583 to 0.743	No	ns	>0.9999
cdNV_RolPA_C vs. cdNV_DMEM_C	-0.01667	-0.6797 to 0.6464	No	ns	>0.9999

cdNV_RolPA_C vs. sn_S80_A	0.2367	-0.4264 to 0.8997	No	ns	>0.9999
cdNV_RolPA_C vs. sn_S80_B	0.03667	-0.6264 to 0.6997	No	ns	>0.9999
cdNV_RolPA_C vs. sn_S80_C	0.03	-0.633 to 0.693	No	ns	>0.9999
cdNV_RolPA_C vs. sn_RolPA_A	0.07667	-0.5864 to 0.7397	No	ns	>0.9999
cdNV_RolPA_C vs. sn_RolPA_B	-0.1	-0.763 to 0.563	No	ns	>0.9999
cdNV_RolPA_C vs. sn_RolPA_C	0.1	-0.563 to 0.763	No	ns	>0.9999
cdNV_RolPA_C vs. sn_TGF_A	0.01	-0.653 to 0.673	No	ns	>0.9999
cdNV_RolPA_C vs. sn_TGF_B	-0.09	-0.753 to 0.573	No	ns	>0.9999
cdNV_RolPA_C vs. sn_TGF_C	-0.006667	-0.6697 to 0.6564	No	ns	>0.9999
cdNV_RolPA_C vs. sn_DMEM_A	0.1167	-0.5464 to 0.7797	No	ns	>0.9999
cdNV_RolPA_C vs. sn_DMEM_B	0.04667	-0.6164 to 0.7097	No	ns	>0.9999
cdNV_RolPA_C vs. sn_DMEM_C	-0.1033	-0.7664 to 0.5597	No	ns	>0.9999
cdNV_RolPA_C vs. S80_A	0.01667	-0.6464 to 0.6797	No	ns	>0.9999
cdNV_RolPA_C vs. S80_B	-0.3233	-0.9864 to 0.3397	No	ns	0.9875
cdNV_RolPA_C vs. S80_C	-0.2567	-0.9197 to 0.4064	No	ns	0.9997
cdNV_TGF_C vs. cdNV_DMEM_C	-0.09667	-0.7597 to 0.5664	No	ns	>0.9999
cdNV_TGF_C vs. sn_S80_A	0.1567	-0.5064 to 0.8197	No	ns	>0.9999
cdNV_TGF_C vs. sn_S80_B	-0.04333	-0.7064 to 0.6197	No	ns	>0.9999
cdNV_TGF_C vs. sn_S80_C	-0.05	-0.713 to 0.613	No	ns	>0.9999
cdNV_TGF_C vs. sn_RolPA_A	-0.003333	-0.6664 to 0.6597	No	ns	>0.9999
cdNV_TGF_C vs. sn_RolPA_B	-0.18	-0.843 to 0.483	No	ns	>0.9999
cdNV_TGF_C vs. sn_RolPA_C	0.02	-0.643 to 0.683	No	ns	>0.9999
cdNV_TGF_C vs. sn_TGF_A	-0.07	-0.733 to 0.593	No	ns	>0.9999
cdNV_TGF_C vs. sn_TGF_B	-0.17	-0.833 to 0.493	No	ns	>0.9999
cdNV_TGF_C vs. sn_TGF_C	-0.08667	-0.7497 to 0.5764	No	ns	>0.9999
cdNV_TGF_C vs. sn_DMEM_A	0.03667	-0.6264 to 0.6997	No	ns	>0.9999
cdNV_TGF_C vs. sn_DMEM_B	-0.03333	-0.6964 to 0.6297	No	ns	>0.9999
cdNV_TGF_C vs. sn_DMEM_C	-0.1833	-0.8464 to 0.4797	No	ns	>0.9999
cdNV_TGF_C vs. S80_A	-0.06333	-0.7264 to 0.5997	No	ns	>0.9999
cdNV_TGF_C vs. S80_B	-0.4033	-1.066 to 0.2597	No	ns	0.8572
cdNV_TGF_C vs. S80_C	-0.3367	-0.9997 to 0.3264	No	ns	0.9788
cdNV_DMEM_C vs. sn_S80_A	0.2533	-0.4097 to 0.9164	No	ns	0.9998
cdNV_DMEM_C vs. sn_S80_B	0.05333	-0.6097 to 0.7164	No	ns	>0.9999
cdNV_DMEM_C vs. sn_S80_C	0.04667	-0.6164 to 0.7097	No	ns	>0.9999
cdNV_DMEM_C vs. sn_RolPA_A	0.09333	-0.5697 to 0.7564	No	ns	>0.9999
cdNV_DMEM_C vs. sn_RolPA_B	-0.08333	-0.7464 to 0.5797	No	ns	>0.9999
cdNV_DMEM_C vs. sn_RolPA_C	0.1167	-0.5464 to 0.7797	No	ns	>0.9999
cdNV_DMEM_C vs. sn_TGF_A	0.02667	-0.6364 to 0.6897	No	ns	>0.9999
cdNV_DMEM_C vs. sn_TGF_B	-0.07333	-0.7364 to 0.5897	No	ns	>0.9999
cdNV_DMEM_C vs. sn_TGF_C	0.01	-0.653 to 0.673	No	ns	>0.9999
cdNV_DMEM_C vs. sn_DMEM_A	0.1333	-0.5297 to 0.7964	No	ns	>0.9999
cdNV_DMEM_C vs. sn_DMEM_B	0.06333	-0.5997 to 0.7264	No	ns	>0.9999
cdNV_DMEM_C vs. sn_DMEM_C	-0.08667	-0.7497 to 0.5764	No	ns	>0.9999
cdNV_DMEM_C vs. S80_A	0.03333	-0.6297 to 0.6964	No	ns	>0.9999
cdNV_DMEM_C vs. S80_B	-0.3067	-0.9697 to 0.3564	No	ns	0.9941
cdNV_DMEM_C vs. S80_C	-0.24	-0.903 to 0.423	No	ns	>0.9999
sn_S80_A vs. sn_S80_B	-0.2	-0.863 to 0.463	No	ns	>0.9999
sn_S80_A vs. sn_S80_C	-0.2067	-0.8697 to 0.4564	No	ns	>0.9999
sn_S80_A vs. sn_RolPA_A	-0.16	-0.823 to 0.503	No	ns	>0.9999
sn_S80_A vs. sn_RolPA_B	-0.3367	-0.9997 to 0.3264	No	ns	0.9788
sn_S80_A vs. sn_RolPA_C	-0.1367	-0.7997 to 0.5264	No	ns	>0.9999
sn_S80_A vs. sn_TGF_A	-0.2267	-0.8897 to 0.4364	No	ns	>0.9999
sn_S80_A vs. sn_TGF_B	-0.3267	-0.9897 to 0.3364	No	ns	0.9856
sn_S80_A vs. sn_TGF_C	-0.2433	-0.9064 to 0.4197	No	ns	0.9999
sn_S80_A vs. sn_DMEM_A	-0.12	-0.783 to 0.543	No	ns	>0.9999
sn_S80_A vs. sn_DMEM_B	-0.19	-0.853 to 0.473	No	ns	>0.9999

sn_S80_A vs. sn_DMEM_C	-0.34	-1.003 to 0.323	No	ns	0.976
sn_S80_A vs. S80_A	-0.22	-0.883 to 0.443	No	ns	>0.9999
sn_S80_A vs. S80_B	-0.56	-1.223 to 0.103	No	ns	0.2373
sn_S80_A vs. S80_C	-0.4933	-1.156 to 0.1697	No	ns	0.4911
sn_S80_B vs. sn_S80_C	-0.006667	-0.6697 to 0.6564	No	ns	>0.9999
sn_S80_B vs. sn_RolPA_A	0.04	-0.623 to 0.703	No	ns	>0.9999
sn_S80_B vs. sn_RolPA_B	-0.1367	-0.7997 to 0.5264	No	ns	>0.9999
sn_S80_B vs. sn_RolPA_C	0.06333	-0.5997 to 0.7264	No	ns	>0.9999
sn_S80_B vs. sn_TGF_A	-0.02667	-0.6897 to 0.6364	No	ns	>0.9999
sn_S80_B vs. sn_TGF_B	-0.1267	-0.7897 to 0.5364	No	ns	>0.9999
sn_S80_B vs. sn_TGF_C	-0.04333	-0.7064 to 0.6197	No	ns	>0.9999
sn_S80_B vs. sn_DMEM_A	0.08	-0.583 to 0.743	No	ns	>0.9999
sn_S80_B vs. sn_DMEM_B	0.01	-0.653 to 0.673	No	ns	>0.9999
sn_S80_B vs. sn_DMEM_C	-0.14	-0.803 to 0.523	No	ns	>0.9999
sn_S80_B vs. S80_A	-0.02	-0.683 to 0.643	No	ns	>0.9999
sn_S80_B vs. S80_B	-0.36	-1.023 to 0.303	No	ns	0.953
sn_S80_B vs. S80_C	-0.2933	-0.9564 to 0.3697	No	ns	0.997
sn_S80_C vs. sn_RolPA_A	0.04667	-0.6164 to 0.7097	No	ns	>0.9999
sn_S80_C vs. sn_RolPA_B	-0.13	-0.793 to 0.533	No	ns	>0.9999
sn_S80_C vs. sn_RolPA_C	0.07	-0.593 to 0.733	No	ns	>0.9999
sn_S80_C vs. sn_TGF_A	-0.02	-0.683 to 0.643	No	ns	>0.9999
sn_S80_C vs. sn_TGF_B	-0.12	-0.783 to 0.543	No	ns	>0.9999
sn_S80_C vs. sn_TGF_C	-0.03667	-0.6997 to 0.6264	No	ns	>0.9999
sn_S80_C vs. sn_DMEM_A	0.08667	-0.5764 to 0.7497	No	ns	>0.9999
sn_S80_C vs. sn_DMEM_B	0.01667	-0.6464 to 0.6797	No	ns	>0.9999
sn_S80_C vs. sn_DMEM_C	-0.1333	-0.7964 to 0.5297	No	ns	>0.9999
sn_S80_C vs. S80_A	-0.01333	-0.6764 to 0.6497	No	ns	>0.9999
sn_S80_C vs. S80_B	-0.3533	-1.016 to 0.3097	No	ns	0.962
sn_S80_C vs. S80_C	-0.2867	-0.9497 to 0.3764	No	ns	0.9979
sn_RolPA_A vs. sn_RolPA_B	-0.1767	-0.8397 to 0.4864	No	ns	>0.9999
sn_RolPA_A vs. sn_RolPA_C	0.02333	-0.6397 to 0.6864	No	ns	>0.9999
sn_RolPA_A vs. sn_TGF_A	-0.06667	-0.7297 to 0.5964	No	ns	>0.9999
sn_RolPA_A vs. sn_TGF_B	-0.1667	-0.8297 to 0.4964	No	ns	>0.9999
sn_RolPA_A vs. sn_TGF_C	-0.08333	-0.7464 to 0.5797	No	ns	>0.9999
sn_RolPA_A vs. sn_DMEM_A	0.04	-0.623 to 0.703	No	ns	>0.9999
sn_RolPA_A vs. sn_DMEM_B	-0.03	-0.693 to 0.633	No	ns	>0.9999
sn_RolPA_A vs. sn_DMEM_C	-0.18	-0.843 to 0.483	No	ns	>0.9999
sn_RolPA_A vs. S80_A	-0.06	-0.723 to 0.603	No	ns	>0.9999
sn_RolPA_A vs. S80_B	-0.4	-1.063 to 0.263	No	ns	0.8671
sn_RolPA_A vs. S80_C	-0.3333	-0.9964 to 0.3297	No	ns	0.9813
sn_RolPA_B vs. sn_RolPA_C	0.2	-0.463 to 0.863	No	ns	>0.9999
sn_RolPA_B vs. sn_TGF_A	0.11	-0.553 to 0.773	No	ns	>0.9999
sn_RolPA_B vs. sn_TGF_B	0.01	-0.653 to 0.673	No	ns	>0.9999
sn_RolPA_B vs. sn_TGF_C	0.09333	-0.5697 to 0.7564	No	ns	>0.9999
sn_RolPA_B vs. sn_DMEM_A	0.2167	-0.4464 to 0.8797	No	ns	>0.9999
sn_RolPA_B vs. sn_DMEM_B	0.1467	-0.5164 to 0.8097	No	ns	>0.9999
sn_RolPA_B vs. sn_DMEM_C	-0.003333	-0.6664 to 0.6597	No	ns	>0.9999
sn_RolPA_B vs. S80_A	0.1167	-0.5464 to 0.7797	No	ns	>0.9999
sn_RolPA_B vs. S80_B	-0.2233	-0.8864 to 0.4397	No	ns	>0.9999
sn_RolPA_B vs. S80_C	-0.1567	-0.8197 to 0.5064	No	ns	>0.9999
sn_RolPA_C vs. sn_TGF_A	-0.09	-0.753 to 0.573	No	ns	>0.9999
sn_RolPA_C vs. sn_TGF_B	-0.19	-0.853 to 0.473	No	ns	>0.9999
sn_RolPA_C vs. sn_TGF_C	-0.1067	-0.7697 to 0.5564	No	ns	>0.9999
sn_RolPA_C vs. sn_DMEM_A	0.01667	-0.6464 to 0.6797	No	ns	>0.9999
sn_RolPA_C vs. sn_DMEM_B	-0.05333	-0.7164 to 0.6097	No	ns	>0.9999
sn_RolPA_C vs. sn_DMEM_C	-0.2033	-0.8664 to 0.4597	No	ns	>0.9999

sn_RolPA_C vs. S80_A	-0.08333	-0.7464 to 0.5797	No	ns	>0.9999
sn_RolPA_C vs. S80_B	-0.4233	-1.086 to 0.2397	No	ns	0.7903
sn_RolPA_C vs. S80_C	-0.3567	-1.02 to 0.3064	No	ns	0.9577
sn_TGF_A vs. sn_TGF_B	-0.1	-0.763 to 0.563	No	ns	>0.9999
sn_TGF_A vs. sn_TGF_C	-0.01667	-0.6797 to 0.6464	No	ns	>0.9999
sn_TGF_A vs. sn_DMEM_A	0.1067	-0.5564 to 0.7697	No	ns	>0.9999
sn_TGF_A vs. sn_DMEM_B	0.03667	-0.6264 to 0.6997	No	ns	>0.9999
sn_TGF_A vs. sn_DMEM_C	-0.1133	-0.7764 to 0.5497	No	ns	>0.9999
sn_TGF_A vs. S80_A	0.006667	-0.6564 to 0.6697	No	ns	>0.9999
sn_TGF_A vs. S80_B	-0.3333	-0.9964 to 0.3297	No	ns	0.9813
sn_TGF_A vs. S80_C	-0.2667	-0.9297 to 0.3964	No	ns	0.9994
sn_TGF_B vs. sn_TGF_C	0.08333	-0.5797 to 0.7464	No	ns	>0.9999
sn_TGF_B vs. sn_DMEM_A	0.2067	-0.4564 to 0.8697	No	ns	>0.9999
sn_TGF_B vs. sn_DMEM_B	0.1367	-0.5264 to 0.7997	No	ns	>0.9999
sn_TGF_B vs. sn_DMEM_C	-0.01333	-0.6764 to 0.6497	No	ns	>0.9999
sn_TGF_B vs. S80_A	0.1067	-0.5564 to 0.7697	No	ns	>0.9999
sn_TGF_B vs. S80_B	-0.2333	-0.8964 to 0.4297	No	ns	>0.9999
sn_TGF_B vs. S80_C	-0.1667	-0.8297 to 0.4964	No	ns	>0.9999
sn_TGF_C vs. sn_DMEM_A	0.1233	-0.5397 to 0.7864	No	ns	>0.9999
sn_TGF_C vs. sn_DMEM_B	0.05333	-0.6097 to 0.7164	No	ns	>0.9999
sn_TGF_C vs. sn_DMEM_C	-0.09667	-0.7597 to 0.5664	No	ns	>0.9999
sn_TGF_C vs. S80_A	0.02333	-0.6397 to 0.6864	No	ns	>0.9999
sn_TGF_C vs. S80_B	-0.3167	-0.9797 to 0.3464	No	ns	0.9906
sn_TGF_C vs. S80_C	-0.25	-0.913 to 0.413	No	ns	0.9998
sn_DMEM_A vs. sn_DMEM_B	-0.07	-0.733 to 0.593	No	ns	>0.9999
sn_DMEM_A vs. sn_DMEM_C	-0.22	-0.883 to 0.443	No	ns	>0.9999
sn_DMEM_A vs. S80_A	-0.1	-0.763 to 0.563	No	ns	>0.9999
sn_DMEM_A vs. S80_B	-0.44	-1.103 to 0.223	No	ns	0.7254
sn_DMEM_A vs. S80_C	-0.3733	-1.036 to 0.2897	No	ns	0.9308
sn_DMEM_B vs. sn_DMEM_C	-0.15	-0.813 to 0.513	No	ns	>0.9999
sn_DMEM_B vs. S80_A	-0.03	-0.693 to 0.633	No	ns	>0.9999
sn_DMEM_B vs. S80_B	-0.37	-1.033 to 0.293	No	ns	0.9369
sn_DMEM_B vs. S80_C	-0.3033	-0.9664 to 0.3597	No	ns	0.995
sn_DMEM_C vs. S80_A	0.12	-0.543 to 0.783	No	ns	>0.9999
sn_DMEM_C vs. S80_B	-0.22	-0.883 to 0.443	No	ns	>0.9999
sn_DMEM_C vs. S80_C	-0.1533	-0.8164 to 0.5097	No	ns	>0.9999
S80_A vs. S80_B	-0.34	-1.003 to 0.323	No	ns	0.976
S80_A vs. S80_C	-0.2733	-0.9364 to 0.3897	No	ns	0.9991
S80_B vs. S80_C	0.06667	-0.5964 to 0.7297	No	ns	>0.9999

Table S8. Statistical analysis.
Results of one-way ANOVA test with a post-hoc Tukey test of SPARC mRNA transcription in Figure 4c and Figure S5.

**Number of families**	1				
**Number of comparisons per family**	528				
**Alpha**	0.05				
**Tukey's multiple comparisons test**	**Mean Diff.**	**95.00% CI of diff.**	**Significant ?**	**Summar y**	**Adjusted P Value**
DMEM vs. RolPA	0.5175	-0.07065 to 1.106	No	ns	0.1735
DMEM vs. TGF-ß1	-0.358	-0.9097 to 0.1937	No	ns	0.7633

DMEM vs. S80	0.1425	-0.4457 to 0.7307	No	ns	>0.9999
DMEM vs. PBS	-0.3675	-0.9557 to 0.2207	No	ns	0.8213
DMEM vs. HEPES	-0.1125	-0.7007 to 0.4757	No	ns	>0.9999
DMEM vs. cdNV_S80_A	0.3233	-0.321 to 0.9676	No	ns	0.9815
DMEM vs. cdNV_RolPA_A	0.2333	-0.411 to 0.8776	No	ns	>0.9999
DMEM vs. cdNV_TGF_A	0.2633	-0.381 to 0.9076	No	ns	0.9992
DMEM vs. cdNV_DMEM_A	0.4167	-0.2276 to 1.061	No	ns	0.7688
DMEM vs. cdNV_S80_B	-0.01333	-0.6576 to 0.631	No	ns	>0.9999
DMEM vs. cdNV_RolPA_B	0.02667	-0.6176 to 0.671	No	ns	>0.9999
DMEM vs. cdNV_TGF_B	0.1467	-0.4976 to 0.791	No	ns	>0.9999
DMEM vs. cdNV_DMEM_B	0.02333	-0.621 to 0.6676	No	ns	>0.9999
DMEM vs. cdNV_S80_C	-0.2	-0.8443 to 0.4443	No	ns	>0.9999
DMEM vs. cdNV_RolPA_C	-0.04	-0.6843 to 0.6043	No	ns	>0.9999
DMEM vs. cdNV_TGF_C	0.03667	-0.6076 to 0.681	No	ns	>0.9999
DMEM vs. cdNV_DMEM_C	0.03	-0.6143 to 0.6743	No	ns	>0.9999
DMEM vs. sn_S80_A	0.055	-0.689 to 0.799	No	ns	>0.9999
DMEM vs. sn_S80_B	-0.155	-0.899 to 0.589	No	ns	>0.9999
DMEM vs. sn_S80_C	-0.06667	-0.711 to 0.5776	No	ns	>0.9999
DMEM vs. sn_RolPA_A	0.04	-0.6043 to 0.6843	No	ns	>0.9999
DMEM vs. sn_RolPA_B	-0.05	-0.6943 to 0.5943	No	ns	>0.9999
DMEM vs. sn_RolPA_C	0.09	-0.5543 to 0.7343	No	ns	>0.9999
DMEM vs. sn_TGF_A	0.06	-0.5843 to 0.7043	No	ns	>0.9999
DMEM vs. sn_TGF_B	-0.07	-0.7143 to 0.5743	No	ns	>0.9999
DMEM vs. sn_TGF_C	-0.1	-0.7443 to 0.5443	No	ns	>0.9999
DMEM vs. sn_DMEM_A	0.05333	-0.591 to 0.6976	No	ns	>0.9999
DMEM vs. sn_DMEM_B	0.01333	-0.631 to 0.6576	No	ns	>0.9999
DMEM vs. sn_DMEM_C	-0.1633	-0.8076 to 0.481	No	ns	>0.9999
DMEM vs. S80_A	0.01	-0.6343 to 0.6543	No	ns	>0.9999
DMEM vs. S80_B	-0.3933	-1.038 to 0.251	No	ns	0.852
DMEM vs. S80_C	-0.2333	-0.8776 to 0.411	No	ns	>0.9999
RolPA vs. TGF-ß1	-0.8755	-1.487 to -0.2643	Yes	***	0.0001
RolPA vs. S80	-0.375	-1.019 to 0.2693	No	ns	0.9033
RolPA vs. PBS	-0.885	-1.529 to -0.2407	Yes	***	0.0003
RolPA vs. HEPES	-0.63	-1.274 to 0.01429	No	ns	0.0641
RolPA vs. cdNV_S80_A	-0.1942	-0.8901 to 0.5017	No	ns	>0.9999
RolPA vs. cdNV_RolPA_A	-0.2842	-0.9801 to 0.4117	No	ns	0.9992
RolPA vs. cdNV_TGF_A	-0.2542	-0.9501 to 0.4417	No	ns	0.9999
RolPA vs. cdNV_DMEM_A	-0.1008	-0.7967 to 0.5951	No	ns	>0.9999
RolPA vs. cdNV_S80_B	-0.5308	-1.227 to 0.1651	No	ns	0.4364
RolPA vs. cdNV_RolPA_B	-0.4908	-1.187 to 0.2051	No	ns	0.6048
RolPA vs. cdNV_TGF_B	-0.3708	-1.067 to 0.3251	No	ns	0.9616
RolPA vs. cdNV_DMEM_B	-0.4942	-1.19 to 0.2017	No	ns	0.5906
RolPA vs. cdNV_S80_C	-0.7175	-1.413 to -0.02159	Yes	*	0.0349
RolPA vs. cdNV_RolPA_C	-0.5575	-1.253 to 0.1384	No	ns	0.3345
RolPA vs. cdNV_TGF_C	-0.4808	-1.177 to 0.2151	No	ns	0.647
RolPA vs. cdNV_DMEM_C	-0.4875	-1.183 to 0.2084	No	ns	0.6189

RolPA vs. sn_S80_A	-0.4625	-1.252 to 0.3266	No	ns	0.8967
RolPA vs. sn_S80_B	-0.6725	-1.462 to 0.1166	No	ns	0.2219
RolPA vs. sn_S80_C	-0.5842	-1.28 to 0.1117	No	ns	0.2474
RolPA vs. sn_RolPA_A	-0.4775	-1.173 to 0.2184	No	ns	0.6609
RolPA vs. sn_RolPA_B	-0.5675	-1.263 to 0.1284	No	ns	0.2999
RolPA vs. sn_RolPA_C	-0.4275	-1.123 to 0.2684	No	ns	0.8441
RolPA vs. sn_TGF_A	-0.4575	-1.153 to 0.2384	No	ns	0.741
RolPA vs. sn_TGF_B	-0.5875	-1.283 to 0.1084	No	ns	0.2376
RolPA vs. sn_TGF_C	-0.6175	-1.313 to 0.07841	No	ns	0.1619
RolPA vs. sn_DMEM_A	-0.4642	-1.16 to 0.2317	No	ns	0.715
RolPA vs. sn_DMEM_B	-0.5042	-1.2 to 0.1917	No	ns	0.5478
RolPA vs. sn_DMEM_C	-0.6808	-1.377 to 0.01508	No	ns	0.0638
RolPA vs. S80_A	-0.5075	-1.203 to 0.1884	No	ns	0.5336
RolPA vs. S80_B	-0.9108	-1.607 to -0.2149	Yes	***	0.0008
RolPA vs. S80_C	-0.7508	-1.447 to -0.05492	Yes	*	0.0194
TGF-ß1 vs. S80	0.5005	-0.1107 to 1.112	No	ns	0.2922
TGF-ß1 vs. PBS	-0.0095	-0.6207 to 0.6017	No	ns	>0.9999
TGF-ß1 vs. HEPES	0.2455	-0.3657 to 0.8567	No	ns	0.9994
TGF-ß1 vs. cdNV_S80_A	0.6813	0.01591 to 1.347	Yes	*	0.0379
TGF-ß1 vs. cdNV_RolPA_A	0.5913	-0.07409 to 1.257	No	ns	0.1598
TGF-ß1 vs. cdNV_TGF_A	0.6213	-0.04409 to 1.287	No	ns	0.1026
TGF-ß1 vs. cdNV_DMEM_A	0.7747	0.1092 to 1.44	Yes	**	0.0064
TGF-ß1 vs. cdNV_S80_B	0.3447	-0.3208 to 1.01	No	ns	0.9725
TGF-ß1 vs. cdNV_RolPA_B	0.3847	-0.2808 to 1.05	No	ns	0.9094
TGF-ß1 vs. cdNV_TGF_B	0.5047	-0.1608 to 1.17	No	ns	0.4487
TGF-ß1 vs. cdNV_DMEM_B	0.3813	-0.2841 to 1.047	No	ns	0.9167
TGF-ß1 vs. cdNV_S80_C	0.158	-0.5074 to 0.8234	No	ns	>0.9999
TGF-ß1 vs. cdNV_RolPA_C	0.318	-0.3474 to 0.9834	No	ns	0.9904
TGF-ß1 vs. cdNV_TGF_C	0.3947	-0.2708 to 1.06	No	ns	0.8849
TGF-ß1 vs. cdNV_DMEM_C	0.388	-0.2774 to 1.053	No	ns	0.9016
TGF-ß1 vs. sn_S80_A	0.413	-0.3493 to 1.175	No	ns	0.9537
TGF-ß1 vs. sn_S80_B	0.203	-0.5593 to 0.9653	No	ns	>0.9999
TGF-ß1 vs. sn_S80_C	0.2913	-0.3741 to 0.9568	No	ns	0.9974
TGF-ß1 vs. sn_RolPA_A	0.398	-0.2674 to 1.063	No	ns	0.8759
TGF-ß1 vs. sn_RolPA_B	0.308	-0.3574 to 0.9734	No	ns	0.9939
TGF-ß1 vs. sn_RolPA_C	0.448	-0.2174 to 1.113	No	ns	0.6976
TGF-ß1 vs. sn_TGF_A	0.418	-0.2474 to 1.083	No	ns	0.8137
TGF-ß1 vs. sn_TGF_B	0.288	-0.3774 to 0.9534	No	ns	0.9978
TGF-ß1 vs. sn_TGF_C	0.258	-0.4074 to 0.9234	No	ns	0.9997
TGF-ß1 vs. sn_DMEM_A	0.4113	-0.2541 to 1.077	No	ns	0.836
TGF-ß1 vs. sn_DMEM_B	0.3713	-0.2941 to 1.037	No	ns	0.9364
TGF-ß1 vs. sn_DMEM_C	0.1947	-0.4708 to 0.8601	No	ns	>0.9999
TGF-ß1 vs. S80_A	0.368	-0.2974 to 1.033	No	ns	0.9421
TGF-ß1 vs. S80_B	-0.03533	-0.7008 to 0.6301	No	ns	>0.9999
TGF-ß1 vs. S80_C	0.1247	-0.5408 to 0.7901	No	ns	>0.9999
S80 vs. PBS	-0.51	-1.154 to 0.1343	No	ns	0.3586
S80 vs. HEPES	-0.255	-0.8993 to 0.3893	No	ns	0.9995

S80 vs. cdNV_S80_A	0.1808	-0.5151 to 0.8767	No	ns	>0.9999
S80 vs. cdNV_RolPA_A	0.09083	-0.6051 to 0.7867	No	ns	>0.9999
S80 vs. cdNV_TGF_A	0.1208	-0.5751 to 0.8167	No	ns	>0.9999
S80 vs. cdNV_DMEM_A	0.2742	-0.4217 to 0.9701	No	ns	0.9996
S80 vs. cdNV_S80_B	-0.1558	-0.8517 to 0.5401	No	ns	>0.9999
S80 vs. cdNV_RolPA_B	-0.1158	-0.8117 to 0.5801	No	ns	>0.9999
S80 vs. cdNV_TGF_B	0.004167	-0.6917 to 0.7001	No	ns	>0.9999
S80 vs. cdNV_DMEM_B	-0.1192	-0.8151 to 0.5767	No	ns	>0.9999
S80 vs. cdNV_S80_C	-0.3425	-1.038 to 0.3534	No	ns	0.9857
S80 vs. cdNV_RolPA_C	-0.1825	-0.8784 to 0.5134	No	ns	>0.9999
S80 vs. cdNV_TGF_C	-0.1058	-0.8017 to 0.5901	No	ns	>0.9999
S80 vs. cdNV_DMEM_C	-0.1125	-0.8084 to 0.5834	No	ns	>0.9999
S80 vs. sn_S80_A	-0.0875	-0.8766 to 0.7016	No	ns	>0.9999
S80 vs. sn_S80_B	-0.2975	-1.087 to 0.4916	No	ns	0.9998
S80 vs. sn_S80_C	-0.2092	-0.9051 to 0.4867	No	ns	>0.9999
S80 vs. sn_RolPA_A	-0.1025	-0.7984 to 0.5934	No	ns	>0.9999
S80 vs. sn_RolPA_B	-0.1925	-0.8884 to 0.5034	No	ns	>0.9999
S80 vs. sn_RolPA_C	-0.0525	-0.7484 to 0.6434	No	ns	>0.9999
S80 vs. sn_TGF_A	-0.0825	-0.7784 to 0.6134	No	ns	>0.9999
S80 vs. sn_TGF_B	-0.2125	-0.9084 to 0.4834	No	ns	>0.9999
S80 vs. sn_TGF_C	-0.2425	-0.9384 to 0.4534	No	ns	>0.9999
S80 vs. sn_DMEM_A	-0.08917	-0.7851 to 0.6067	No	ns	>0.9999
S80 vs. sn_DMEM_B	-0.1292	-0.8251 to 0.5667	No	ns	>0.9999
S80 vs. sn_DMEM_C	-0.3058	-1.002 to 0.3901	No	ns	0.9972
S80 vs. S80_A	-0.1325	-0.8284 to 0.5634	No	ns	>0.9999
S80 vs. S80_B	-0.5358	-1.232 to 0.1601	No	ns	0.4164
S80 vs. S80_C	-0.3758	-1.072 to 0.3201	No	ns	0.9553
PBS vs. HEPES	0.255	-0.3893 to 0.8993	No	ns	0.9995
PBS vs. cdNV_S80_A	0.6908	-0.00508 to 1.387	No	ns	0.0543
PBS vs. cdNV_RolPA_A	0.6008	-0.09508 to 1.297	No	ns	0.2013
PBS vs. cdNV_TGF_A	0.6308	-0.06508 to 1.327	No	ns	0.1348
PBS vs. cdNV_DMEM_A	0.7842	0.08825 to 1.48	Yes	*	0.0105
PBS vs. cdNV_S80_B	0.3542	-0.3417 to 1.05	No	ns	0.9779
PBS vs. cdNV_RolPA_B	0.3942	-0.3017 to 1.09	No	ns	0.9259
PBS vs. cdNV_TGF_B	0.5142	-0.1817 to 1.21	No	ns	0.5054
PBS vs. cdNV_DMEM_B	0.3908	-0.3051 to 1.087	No	ns	0.932
PBS vs. cdNV_S80_C	0.1675	-0.5284 to 0.8634	No	ns	>0.9999
PBS vs. cdNV_RolPA_C	0.3275	-0.3684 to 1.023	No	ns	0.9923
PBS vs. cdNV_TGF_C	0.4042	-0.2917 to 1.1	No	ns	0.9053
PBS vs. cdNV_DMEM_C	0.3975	-0.2984 to 1.093	No	ns	0.9194
PBS vs. sn_S80_A	0.4225	-0.3666 to 1.212	No	ns	0.9595
PBS vs. sn_S80_B	0.2125	-0.5766 to 1.002	No	ns	>0.9999

PBS vs. sn_S80_C	0.3008	-0.3951 to 0.9967	No	ns	0.9979
PBS vs. sn_RolPA_A	0.4075	-0.2884 to 1.103	No	ns	0.8977
PBS vs. sn_RolPA_B	0.3175	-0.3784 to 1.013	No	ns	0.9951
PBS vs. sn_RolPA_C	0.4575	-0.2384 to 1.153	No	ns	0.741
PBS vs. sn_TGF_A	0.4275	-0.2684 to 1.123	No	ns	0.8441
PBS vs. sn_TGF_B	0.2975	-0.3984 to 0.9934	No	ns	0.9982
PBS vs. sn_TGF_C	0.2675	-0.4284 to 0.9634	No	ns	0.9997
PBS vs. sn_DMEM_A	0.4208	-0.2751 to 1.117	No	ns	0.8634
PBS vs. sn_DMEM_B	0.3808	-0.3151 to 1.077	No	ns	0.9483
PBS vs. sn_DMEM_C	0.2042	-0.4917 to 0.9001	No	ns	>0.9999
PBS vs. S80_A	0.3775	-0.3184 to 1.073	No	ns	0.9531
PBS vs. S80_B	-0.02583	-0.7217 to 0.6701	No	ns	>0.9999
PBS vs. S80_C	0.1342	-0.5617 to 0.8301	No	ns	>0.9999
HEPES vs. cdNV_S80_A	0.4358	-0.2601 to 1.132	No	ns	0.818
HEPES vs. cdNV_RolPA_A	0.3458	-0.3501 to 1.042	No	ns	0.9837
HEPES vs. cdNV_TGF_A	0.3758	-0.3201 to 1.072	No	ns	0.9553
HEPES vs. cdNV_DMEM_A	0.5292	-0.1667 to 1.225	No	ns	0.4431
HEPES vs. cdNV_S80_B	0.09917	-0.5967 to 0.7951	No	ns	>0.9999
HEPES vs. cdNV_RolPA_B	0.1392	-0.5567 to 0.8351	No	ns	>0.9999
HEPES vs. cdNV_TGF_B	0.2592	-0.4367 to 0.9551	No	ns	0.9998
HEPES vs. cdNV_DMEM_B	0.1358	-0.5601 to 0.8317	No	ns	>0.9999
HEPES vs. cdNV_S80_C	-0.0875	-0.7834 to 0.6084	No	ns	>0.9999
HEPES vs. cdNV_RolPA_C	0.0725	-0.6234 to 0.7684	No	ns	>0.9999
HEPES vs. cdNV_TGF_C	0.1492	-0.5467 to 0.8451	No	ns	>0.9999
HEPES vs. cdNV_DMEM_C	0.1425	-0.5534 to 0.8384	No	ns	>0.9999
HEPES vs. sn_S80_A	0.1675	-0.6216 to 0.9566	No	ns	>0.9999
HEPES vs. sn_S80_B	-0.0425	-0.8316 to 0.7466	No	ns	>0.9999
HEPES vs. sn_S80_C	0.04583	-0.6501 to 0.7417	No	ns	>0.9999
HEPES vs. sn_RolPA_A	0.1525	-0.5434 to 0.8484	No	ns	>0.9999
HEPES vs. sn_RolPA_B	0.0625	-0.6334 to 0.7584	No	ns	>0.9999
HEPES vs. sn_RolPA_C	0.2025	-0.4934 to 0.8984	No	ns	>0.9999
HEPES vs. sn_TGF_A	0.1725	-0.5234 to 0.8684	No	ns	>0.9999
HEPES vs. sn_TGF_B	0.0425	-0.6534 to 0.7384	No	ns	>0.9999
HEPES vs. sn_TGF_C	0.0125	-0.6834 to 0.7084	No	ns	>0.9999
HEPES vs. sn_DMEM_A	0.1658	-0.5301 to 0.8617	No	ns	>0.9999
HEPES vs. sn_DMEM_B	0.1258	-0.5701 to 0.8217	No	ns	>0.9999
HEPES vs. sn_DMEM_C	-0.05083	-0.7467 to 0.6451	No	ns	>0.9999
HEPES vs. S80_A	0.1225	-0.5734 to 0.8184	No	ns	>0.9999
HEPES vs. S80_B	-0.2808	-0.9767 to 0.4151	No	ns	0.9993
HEPES vs. S80_C	-0.1208	-0.8167 to 0.5751	No	ns	>0.9999
cdNV_S80_A vs. cdNV_RolPA_A	-0.09	-0.834 to 0.654	No	ns	>0.9999

cdNV_S80_A vs. cdNV_TGF_A	-0.06	-0.804 to 0.684	No	ns	>0.9999
cdNV_S80_A vs. cdNV_DMEM_A	0.09333	-0.6506 to 0.8373	No	ns	>0.9999
cdNV_S80_A vs. cdNV_S80_B	-0.3367	-1.081 to 0.4073	No	ns	0.9957
cdNV_S80_A vs. cdNV_RolPA_B	-0.2967	-1.041 to 0.4473	No	ns	0.9995
cdNV_S80_A vs. cdNV_TGF_B	-0.1767	-0.9206 to 0.5673	No	ns	>0.9999
cdNV_S80_A vs. cdNV_DMEM_B	-0.3	-1.044 to 0.444	No	ns	0.9993
cdNV_S80_A vs. cdNV_S80_C	-0.5233	-1.267 to 0.2206	No	ns	0.6103
cdNV_S80_A vs. cdNV_RolPA_C	-0.3633	-1.107 to 0.3806	No	ns	0.9871
cdNV_S80_A vs. cdNV_TGF_C	-0.2867	-1.031 to 0.4573	No	ns	0.9997
cdNV_S80_A vs. cdNV_DMEM_C	-0.2933	-1.037 to 0.4506	No	ns	0.9996
cdNV_S80_A vs. sn_S80_A	-0.2683	-1.1 to 0.5634	No	ns	>0.9999
cdNV_S80_A vs. sn_S80_B	-0.4783	-1.31 to 0.3534	No	ns	0.9138
cdNV_S80_A vs. sn_S80_C	-0.39	-1.134 to 0.354	No	ns	0.9683
cdNV_S80_A vs. sn_RolPA_A	-0.2833	-1.027 to 0.4606	No	ns	0.9998
cdNV_S80_A vs. sn_RolPA_B	-0.3733	-1.117 to 0.3706	No	ns	0.9815
cdNV_S80_A vs. sn_RolPA_C	-0.2333	-0.9773 to 0.5106	No	ns	>0.9999
cdNV_S80_A vs. sn_TGF_A	-0.2633	-1.007 to 0.4806	No	ns	>0.9999
cdNV_S80_A vs. sn_TGF_B	-0.3933	-1.137 to 0.3506	No	ns	0.965
cdNV_S80_A vs. sn_TGF_C	-0.4233	-1.167 to 0.3206	No	ns	0.9224
cdNV_S80_A vs. sn_DMEM_A	-0.27	-1.014 to 0.474	No	ns	>0.9999
cdNV_S80_A vs. sn_DMEM_B	-0.31	-1.054 to 0.434	No	ns	0.9988
cdNV_S80_A vs. sn_DMEM_C	-0.4867	-1.231 to 0.2573	No	ns	0.7496
cdNV_S80_A vs. S80_A	-0.3133	-1.057 to 0.4306	No	ns	0.9986
cdNV_S80_A vs. S80_B	-0.7167	-1.461 to 0.0273	No	ns	0.0751
cdNV_S80_A vs. S80_C	-0.5567	-1.301 to 0.1873	No	ns	0.4779
cdNV_RolPA_A vs. cdNV_TGF_A	0.03	-0.714 to 0.774	No	ns	>0.9999
cdNV_RolPA_A vs. cdNV_DMEM_A	0.1833	-0.5606 to 0.9273	No	ns	>0.9999
cdNV_RolPA_A vs. cdNV_S80_B	-0.2467	-0.9906 to 0.4973	No	ns	>0.9999
cdNV_RolPA_A vs. cdNV_RolPA_B	-0.2067	-0.9506 to 0.5373	No	ns	>0.9999
cdNV_RolPA_A vs. cdNV_TGF_B	-0.08667	-0.8306 to 0.6573	No	ns	>0.9999
cdNV_RolPA_A vs. cdNV_DMEM_B	-0.21	-0.954 to 0.534	No	ns	>0.9999
cdNV_RolPA_A vs. cdNV_S80_C	-0.4333	-1.177 to 0.3106	No	ns	0.9026
cdNV_RolPA_A vs. cdNV_RolPA_C	-0.2733	-1.017 to 0.4706	No	ns	0.9999
cdNV_RolPA_A vs. cdNV_TGF_C	-0.1967	-0.9406 to 0.5473	No	ns	>0.9999
cdNV_RolPA_A vs. cdNV_DMEM_C	-0.2033	-0.9473 to 0.5406	No	ns	>0.9999
cdNV_RolPA_A vs. sn_S80_A	-0.1783	-1.01 to 0.6534	No	ns	>0.9999
cdNV_RolPA_A vs. sn_S80_B	-0.3883	-1.22 to 0.4434	No	ns	0.9931
cdNV_RolPA_A vs. sn_S80_C	-0.3	-1.044 to 0.444	No	ns	0.9993
cdNV_RolPA_A vs. sn_RolPA_A	-0.1933	-0.9373 to 0.5506	No	ns	>0.9999
cdNV_RolPA_A vs. sn_RolPA_B	-0.2833	-1.027 to 0.4606	No	ns	0.9998
cdNV_RolPA_A vs. sn_RolPA_C	-0.1433	-0.8873 to 0.6006	No	ns	>0.9999
cdNV_RolPA_A vs. sn_TGF_A	-0.1733	-0.9173 to 0.5706	No	ns	>0.9999
cdNV_RolPA_A vs. sn_TGF_B	-0.3033	-1.047 to 0.4406	No	ns	0.9992
cdNV_RolPA_A vs. sn_TGF_C	-0.3333	-1.077 to 0.4106	No	ns	0.9963
cdNV_RolPA_A vs. sn_DMEM_A	-0.18	-0.924 to 0.564	No	ns	>0.9999
cdNV_RolPA_A vs. sn_DMEM_B	-0.22	-0.964 to 0.524	No	ns	>0.9999
cdNV_RolPA_A vs. sn_DMEM_C	-0.3967	-1.141 to 0.3473	No	ns	0.9614
cdNV_RolPA_A vs. S80_A	-0.2233	-0.9673 to 0.5206	No	ns	>0.9999
cdNV_RolPA_A vs. S80_B	-0.6267	-1.371 to 0.1173	No	ns	0.2414

cdNV_RolPA_A vs. S80_C	-0.4667	-1.211 to 0.2773	No	ns	0.8157
cdNV_TGF_A vs. cdNV_DMEM_A	0.1533	-0.5906 to 0.8973	No	ns	>0.9999
cdNV_TGF_A vs. cdNV_S80_B	-0.2767	-1.021 to 0.4673	No	ns	0.9999
cdNV_TGF_A vs. cdNV_RolPA_B	-0.2367	-0.9806 to 0.5073	No	ns	>0.9999
cdNV_TGF_A vs. cdNV_TGF_B	-0.1167	-0.8606 to 0.6273	No	ns	>0.9999
cdNV_TGF_A vs. cdNV_DMEM_B	-0.24	-0.984 to 0.504	No	ns	>0.9999
cdNV_TGF_A vs. cdNV_S80_C	-0.4633	-1.207 to 0.2806	No	ns	0.8258
cdNV_TGF_A vs. cdNV_RolPA_C	-0.3033	-1.047 to 0.4406	No	ns	0.9992
cdNV_TGF_A vs. cdNV_TGF_C	-0.2267	-0.9706 to 0.5173	No	ns	>0.9999
cdNV_TGF_A vs. cdNV_DMEM_C	-0.2333	-0.9773 to 0.5106	No	ns	>0.9999
cdNV_TGF_A vs. sn_S80_A	-0.2083	-1.04 to 0.6234	No	ns	>0.9999
cdNV_TGF_A vs. sn_S80_B	-0.4183	-1.25 to 0.4134	No	ns	0.981
cdNV_TGF_A vs. sn_S80_C	-0.33	-1.074 to 0.414	No	ns	0.9968
cdNV_TGF_A vs. sn_RolPA_A	-0.2233	-0.9673 to 0.5206	No	ns	>0.9999
cdNV_TGF_A vs. sn_RolPA_B	-0.3133	-1.057 to 0.4306	No	ns	0.9986
cdNV_TGF_A vs. sn_RolPA_C	-0.1733	-0.9173 to 0.5706	No	ns	>0.9999
cdNV_TGF_A vs. sn_TGF_A	-0.2033	-0.9473 to 0.5406	No	ns	>0.9999
cdNV_TGF_A vs. sn_TGF_B	-0.3333	-1.077 to 0.4106	No	ns	0.9963
cdNV_TGF_A vs. sn_TGF_C	-0.3633	-1.107 to 0.3806	No	ns	0.9871
cdNV_TGF_A vs. sn_DMEM_A	-0.21	-0.954 to 0.534	No	ns	>0.9999
cdNV_TGF_A vs. sn_DMEM_B	-0.25	-0.994 to 0.494	No	ns	>0.9999
cdNV_TGF_A vs. sn_DMEM_C	-0.4267	-1.171 to 0.3173	No	ns	0.9161
cdNV_TGF_A vs. S80_A	-0.2533	-0.9973 to 0.4906	No	ns	>0.9999
cdNV_TGF_A vs. S80_B	-0.6567	-1.401 to 0.0873	No	ns	0.169
cdNV_TGF_A vs. S80_C	-0.4967	-1.241 to 0.2473	No	ns	0.7134
cdNV_DMEM_A vs. cdNV_S80_B	-0.43	-1.174 to 0.314	No	ns	0.9095
cdNV_DMEM_A vs. cdNV_RolPA_B	-0.39	-1.134 to 0.354	No	ns	0.9683
cdNV_DMEM_A vs. cdNV_TGF_B	-0.27	-1.014 to 0.474	No	ns	>0.9999
cdNV_DMEM_A vs. cdNV_DMEM_B	-0.3933	-1.137 to 0.3506	No	ns	0.965
cdNV_DMEM_A vs. cdNV_S80_C	-0.6167	-1.361 to 0.1273	No	ns	0.2697
cdNV_DMEM_A vs. cdNV_RolPA_C	-0.4567	-1.201 to 0.2873	No	ns	0.8451
cdNV_DMEM_A vs. cdNV_TGF_C	-0.38	-1.124 to 0.364	No	ns	0.9769
cdNV_DMEM_A vs. cdNV_DMEM_C	-0.3867	-1.131 to 0.3573	No	ns	0.9714
cdNV_DMEM_A vs. sn_S80_A	-0.3617	-1.193 to 0.4701	No	ns	0.9977
cdNV_DMEM_A vs. sn_S80_B	-0.5717	-1.403 to 0.2601	No	ns	0.6576
cdNV_DMEM_A vs. sn_S80_C	-0.4833	-1.227 to 0.2606	No	ns	0.7612
cdNV_DMEM_A vs. sn_RolPA_A	-0.3767	-1.121 to 0.3673	No	ns	0.9793
cdNV_DMEM_A vs. sn_RolPA_B	-0.4667	-1.211 to 0.2773	No	ns	0.8157
cdNV_DMEM_A vs. sn_RolPA_C	-0.3267	-1.071 to 0.4173	No	ns	0.9973
cdNV_DMEM_A vs. sn_TGF_A	-0.3567	-1.101 to 0.3873	No	ns	0.99
cdNV_DMEM_A vs. sn_TGF_B	-0.4867	-1.231 to 0.2573	No	ns	0.7496
cdNV_DMEM_A vs. sn_TGF_C	-0.5167	-1.261 to 0.2273	No	ns	0.6367
cdNV_DMEM_A vs. sn_DMEM_A	-0.3633	-1.107 to 0.3806	No	ns	0.9871
cdNV_DMEM_A vs. sn_DMEM_B	-0.4033	-1.147 to 0.3406	No	ns	0.9534
cdNV_DMEM_A vs. sn_DMEM_C	-0.58	-1.324 to 0.164	No	ns	0.3901
cdNV_DMEM_A vs. S80_A	-0.4067	-1.151 to 0.3373	No	ns	0.949
cdNV_DMEM_A vs. S80_B	-0.81	-1.554 to -0.06604	Yes	*	0.0172
cdNV_DMEM_A vs. S80_C	-0.65	-1.394 to 0.09396	No	ns	0.1835
cdNV_S80_B vs. cdNV_RolPA_B	0.04	-0.704 to 0.784	No	ns	>0.9999
cdNV_S80_B vs. cdNV_TGF_B	0.16	-0.584 to 0.904	No	ns	>0.9999

cdNV_S80_B vs. cdNV_DMEM_B	0.03667	-0.7073 to 0.7806	No	ns	>0.9999
cdNV_S80_B vs. cdNV_S80_C	-0.1867	-0.9306 to 0.5573	No	ns	>0.9999
cdNV_S80_B vs. cdNV_RolPA_C	-0.02667	-0.7706 to 0.7173	No	ns	>0.9999
cdNV_S80_B vs. cdNV_TGF_C	0.05	-0.694 to 0.794	No	ns	>0.9999
cdNV_S80_B vs. cdNV_DMEM_C	0.04333	-0.7006 to 0.7873	No	ns	>0.9999
cdNV_S80_B vs. sn_S80_A	0.06833	-0.7634 to 0.9001	No	ns	>0.9999
cdNV_S80_B vs. sn_S80_B	-0.1417	-0.9734 to 0.6901	No	ns	>0.9999
cdNV_S80_B vs. sn_S80_C	-0.05333	-0.7973 to 0.6906	No	ns	>0.9999
cdNV_S80_B vs. sn_RolPA_A	0.05333	-0.6906 to 0.7973	No	ns	>0.9999
cdNV_S80_B vs. sn_RolPA_B	-0.03667	-0.7806 to 0.7073	No	ns	>0.9999
cdNV_S80_B vs. sn_RolPA_C	0.1033	-0.6406 to 0.8473	No	ns	>0.9999
cdNV_S80_B vs. sn_TGF_A	0.07333	-0.6706 to 0.8173	No	ns	>0.9999
cdNV_S80_B vs. sn_TGF_B	-0.05667	-0.8006 to 0.6873	No	ns	>0.9999
cdNV_S80_B vs. sn_TGF_C	-0.08667	-0.8306 to 0.6573	No	ns	>0.9999
cdNV_S80_B vs. sn_DMEM_A	0.06667	-0.6773 to 0.8106	No	ns	>0.9999
cdNV_S80_B vs. sn_DMEM_B	0.02667	-0.7173 to 0.7706	No	ns	>0.9999
cdNV_S80_B vs. sn_DMEM_C	-0.15	-0.894 to 0.594	No	ns	>0.9999
cdNV_S80_B vs. S80_A	0.02333	-0.7206 to 0.7673	No	ns	>0.9999
cdNV_S80_B vs. S80_B	-0.38	-1.124 to 0.364	No	ns	0.9769
cdNV_S80_B vs. S80_C	-0.22	-0.964 to 0.524	No	ns	>0.9999
cdNV_RolPA_B vs. cdNV_TGF_B	0.12	-0.624 to 0.864	No	ns	>0.9999
cdNV_RolPA_B vs. cdNV_DMEM_B	-0.003333	-0.7473 to 0.7406	No	ns	>0.9999
cdNV_RolPA_B vs. cdNV_S80_C	-0.2267	-0.9706 to 0.5173	No	ns	>0.9999
cdNV_RolPA_B vs. cdNV_RolPA_C	-0.06667	-0.8106 to 0.6773	No	ns	>0.9999
cdNV_RolPA_B vs. cdNV_TGF_C	0.01	-0.734 to 0.754	No	ns	>0.9999
cdNV_RolPA_B vs. cdNV_DMEM_C	0.003333	-0.7406 to 0.7473	No	ns	>0.9999
cdNV_RolPA_B vs. sn_S80_A	0.02833	-0.8034 to 0.8601	No	ns	>0.9999
cdNV_RolPA_B vs. sn_S80_B	-0.1817	-1.013 to 0.6501	No	ns	>0.9999
cdNV_RolPA_B vs. sn_S80_C	-0.09333	-0.8373 to 0.6506	No	ns	>0.9999
cdNV_RolPA_B vs. sn_RolPA_A	0.01333	-0.7306 to 0.7573	No	ns	>0.9999
cdNV_RolPA_B vs. sn_RolPA_B	-0.07667	-0.8206 to 0.6673	No	ns	>0.9999
cdNV_RolPA_B vs. sn_RolPA_C	0.06333	-0.6806 to 0.8073	No	ns	>0.9999
cdNV_RolPA_B vs. sn_TGF_A	0.03333	-0.7106 to 0.7773	No	ns	>0.9999
cdNV_RolPA_B vs. sn_TGF_B	-0.09667	-0.8406 to 0.6473	No	ns	>0.9999
cdNV_RolPA_B vs. sn_TGF_C	-0.1267	-0.8706 to 0.6173	No	ns	>0.9999
cdNV_RolPA_B vs. sn_DMEM_A	0.02667	-0.7173 to 0.7706	No	ns	>0.9999
cdNV_RolPA_B vs. sn_DMEM_B	-0.01333	-0.7573 to 0.7306	No	ns	>0.9999
cdNV_RolPA_B vs. sn_DMEM_C	-0.19	-0.934 to 0.554	No	ns	>0.9999
cdNV_RolPA_B vs. S80_A	-0.01667	-0.7606 to 0.7273	No	ns	>0.9999
cdNV_RolPA_B vs. S80_B	-0.42	-1.164 to 0.324	No	ns	0.9283

cdNV_RolPA_B vs. S80_C	-0.26	-1.004 to 0.484	No	ns	>0.9999
cdNV_TGF_B vs. cdNV_DMEM_B	-0.1233	-0.8673 to 0.6206	No	ns	>0.9999
cdNV_TGF_B vs. cdNV_S80_C	-0.3467	-1.091 to 0.3973	No	ns	0.9933
cdNV_TGF_B vs. cdNV_RolPA_C	-0.1867	-0.9306 to 0.5573	No	ns	>0.9999
cdNV_TGF_B vs. cdNV_TGF_C	-0.11	-0.854 to 0.634	No	ns	>0.9999
cdNV_TGF_B vs. cdNV_DMEM_C	-0.1167	-0.8606 to 0.6273	No	ns	>0.9999
cdNV_TGF_B vs. sn_S80_A	-0.09167	-0.9234 to 0.7401	No	ns	>0.9999
cdNV_TGF_B vs. sn_S80_B	-0.3017	-1.133 to 0.5301	No	ns	>0.9999
cdNV_TGF_B vs. sn_S80_C	-0.2133	-0.9573 to 0.5306	No	ns	>0.9999
cdNV_TGF_B vs. sn_RolPA_A	-0.1067	-0.8506 to 0.6373	No	ns	>0.9999
cdNV_TGF_B vs. sn_RolPA_B	-0.1967	-0.9406 to 0.5473	No	ns	>0.9999
cdNV_TGF_B vs. sn_RolPA_C	-0.05667	-0.8006 to 0.6873	No	ns	>0.9999
cdNV_TGF_B vs. sn_TGF_A	-0.08667	-0.8306 to 0.6573	No	ns	>0.9999
cdNV_TGF_B vs. sn_TGF_B	-0.2167	-0.9606 to 0.5273	No	ns	>0.9999
cdNV_TGF_B vs. sn_TGF_C	-0.2467	-0.9906 to 0.4973	No	ns	>0.9999
cdNV_TGF_B vs. sn_DMEM_A	-0.09333	-0.8373 to 0.6506	No	ns	>0.9999
cdNV_TGF_B vs. sn_DMEM_B	-0.1333	-0.8773 to 0.6106	No	ns	>0.9999
cdNV_TGF_B vs. sn_DMEM_C	-0.31	-1.054 to 0.434	No	ns	0.9988
cdNV_TGF_B vs. S80_A	-0.1367	-0.8806 to 0.6073	No	ns	>0.9999
cdNV_TGF_B vs. S80_B	-0.54	-1.284 to 0.204	No	ns	0.5438
cdNV_TGF_B vs. S80_C	-0.38	-1.124 to 0.364	No	ns	0.9769
cdNV_DMEM_B vs. cdNV_S80_C	-0.2233	-0.9673 to 0.5206	No	ns	>0.9999
cdNV_DMEM_B vs. cdNV_RolPA_C	-0.06333	-0.8073 to 0.6806	No	ns	>0.9999
cdNV_DMEM_B vs. cdNV_TGF_C	0.01333	-0.7306 to 0.7573	No	ns	>0.9999
cdNV_DMEM_B vs. cdNV_DMEM_C	0.006667	-0.7373 to 0.7506	No	ns	>0.9999
cdNV_DMEM_B vs. sn_S80_A	0.03167	-0.8001 to 0.8634	No	ns	>0.9999
cdNV_DMEM_B vs. sn_S80_B	-0.1783	-1.01 to 0.6534	No	ns	>0.9999
cdNV_DMEM_B vs. sn_S80_C	-0.09	-0.834 to 0.654	No	ns	>0.9999
cdNV_DMEM_B vs. sn_RolPA_A	0.01667	-0.7273 to 0.7606	No	ns	>0.9999
cdNV_DMEM_B vs. sn_RolPA_B	-0.07333	-0.8173 to 0.6706	No	ns	>0.9999
cdNV_DMEM_B vs. sn_RolPA_C	0.06667	-0.6773 to 0.8106	No	ns	>0.9999
cdNV_DMEM_B vs. sn_TGF_A	0.03667	-0.7073 to 0.7806	No	ns	>0.9999
cdNV_DMEM_B vs. sn_TGF_B	-0.09333	-0.8373 to 0.6506	No	ns	>0.9999
cdNV_DMEM_B vs. sn_TGF_C	-0.1233	-0.8673 to 0.6206	No	ns	>0.9999
cdNV_DMEM_B vs. sn_DMEM_A	0.03	-0.714 to 0.774	No	ns	>0.9999
cdNV_DMEM_B vs. sn_DMEM_B	-0.01	-0.754 to 0.734	No	ns	>0.9999
cdNV_DMEM_B vs. sn_DMEM_C	-0.1867	-0.9306 to 0.5573	No	ns	>0.9999
cdNV_DMEM_B vs. S80_A	-0.01333	-0.7573 to 0.7306	No	ns	>0.9999
cdNV_DMEM_B vs. S80_B	-0.4167	-1.161 to 0.3273	No	ns	0.9339
cdNV_DMEM_B vs. S80_C	-0.2567	-1.001 to 0.4873	No	ns	>0.9999
cdNV_S80_C vs. cdNV_RolPA_C	0.16	-0.584 to 0.904	No	ns	>0.9999
cdNV_S80_C vs. cdNV_TGF_C	0.2367	-0.5073 to 0.9806	No	ns	>0.9999

cdNV_S80_C vs. cdNV_DMEM_C	0.23	-0.514 to 0.974	No	ns	>0.9999
cdNV_S80_C vs. sn_S80_A	0.255	-0.5768 to 1.087	No	ns	>0.9999
cdNV_S80_C vs. sn_S80_B	0.045	-0.7868 to 0.8768	No	ns	>0.9999
cdNV_S80_C vs. sn_S80_C	0.1333	-0.6106 to 0.8773	No	ns	>0.9999
cdNV_S80_C vs. sn_RolPA_A	0.24	-0.504 to 0.984	No	ns	>0.9999
cdNV_S80_C vs. sn_RolPA_B	0.15	-0.594 to 0.894	No	ns	>0.9999
cdNV_S80_C vs. sn_RolPA_C	0.29	-0.454 to 1.034	No	ns	0.9996
cdNV_S80_C vs. sn_TGF_A	0.26	-0.484 to 1.004	No	ns	>0.9999
cdNV_S80_C vs. sn_TGF_B	0.13	-0.614 to 0.874	No	ns	>0.9999
cdNV_S80_C vs. sn_TGF_C	0.1	-0.644 to 0.844	No	ns	>0.9999
cdNV_S80_C vs. sn_DMEM_A	0.2533	-0.4906 to 0.9973	No	ns	>0.9999
cdNV_S80_C vs. sn_DMEM_B	0.2133	-0.5306 to 0.9573	No	ns	>0.9999
cdNV_S80_C vs. sn_DMEM_C	0.03667	-0.7073 to 0.7806	No	ns	>0.9999
cdNV_S80_C vs. S80_A	0.21	-0.534 to 0.954	No	ns	>0.9999
cdNV_S80_C vs. S80_B	-0.1933	-0.9373 to 0.5506	No	ns	>0.9999
cdNV_S80_C vs. S80_C	-0.03333	-0.7773 to 0.7106	No	ns	>0.9999
cdNV_RolPA_C vs. cdNV_TGF_C	0.07667	-0.6673 to 0.8206	No	ns	>0.9999
cdNV_RolPA_C vs. cdNV_DMEM_C	0.07	-0.674 to 0.814	No	ns	>0.9999
cdNV_RolPA_C vs. sn_S80_A	0.095	-0.7368 to 0.9268	No	ns	>0.9999
cdNV_RolPA_C vs. sn_S80_B	-0.115	-0.9468 to 0.7168	No	ns	>0.9999
cdNV_RolPA_C vs. sn_S80_C	-0.02667	-0.7706 to 0.7173	No	ns	>0.9999
cdNV_RolPA_C vs. sn_RolPA_A	0.08	-0.664 to 0.824	No	ns	>0.9999
cdNV_RolPA_C vs. sn_RolPA_B	-0.01	-0.754 to 0.734	No	ns	>0.9999
cdNV_RolPA_C vs. sn_RolPA_C	0.13	-0.614 to 0.874	No	ns	>0.9999
cdNV_RolPA_C vs. sn_TGF_A	0.1	-0.644 to 0.844	No	ns	>0.9999
cdNV_RolPA_C vs. sn_TGF_B	-0.03	-0.774 to 0.714	No	ns	>0.9999
cdNV_RolPA_C vs. sn_TGF_C	-0.06	-0.804 to 0.684	No	ns	>0.9999
cdNV_RolPA_C vs. sn_DMEM_A	0.09333	-0.6506 to 0.8373	No	ns	>0.9999
cdNV_RolPA_C vs. sn_DMEM_B	0.05333	-0.6906 to 0.7973	No	ns	>0.9999
cdNV_RolPA_C vs. sn_DMEM_C	-0.1233	-0.8673 to 0.6206	No	ns	>0.9999
cdNV_RolPA_C vs. S80_A	0.05	-0.694 to 0.794	No	ns	>0.9999
cdNV_RolPA_C vs. S80_B	-0.3533	-1.097 to 0.3906	No	ns	0.9912
cdNV_RolPA_C vs. S80_C	-0.1933	-0.9373 to 0.5506	No	ns	>0.9999
cdNV_TGF_C vs. cdNV_DMEM_C	-0.006667	-0.7506 to 0.7373	No	ns	>0.9999
cdNV_TGF_C vs. sn_S80_A	0.01833	-0.8134 to 0.8501	No	ns	>0.9999
cdNV_TGF_C vs. sn_S80_B	-0.1917	-1.023 to 0.6401	No	ns	>0.9999
cdNV_TGF_C vs. sn_S80_C	-0.1033	-0.8473 to 0.6406	No	ns	>0.9999
cdNV_TGF_C vs. sn_RolPA_A	0.003333	-0.7406 to 0.7473	No	ns	>0.9999
cdNV_TGF_C vs. sn_RolPA_B	-0.08667	-0.8306 to 0.6573	No	ns	>0.9999
cdNV_TGF_C vs. sn_RolPA_C	0.05333	-0.6906 to 0.7973	No	ns	>0.9999
cdNV_TGF_C vs. sn_TGF_A	0.02333	-0.7206 to 0.7673	No	ns	>0.9999
cdNV_TGF_C vs. sn_TGF_B	-0.1067	-0.8506 to 0.6373	No	ns	>0.9999
cdNV_TGF_C vs. sn_TGF_C	-0.1367	-0.8806 to 0.6073	No	ns	>0.9999

cdNV_TGF_C vs. sn_DMEM_A	0.01667	-0.7273 to 0.7606	No	ns	>0.9999
cdNV_TGF_C vs. sn_DMEM_B	-0.02333	-0.7673 to 0.7206	No	ns	>0.9999
cdNV_TGF_C vs. sn_DMEM_C	-0.2	-0.944 to 0.544	No	ns	>0.9999
cdNV_TGF_C vs. S80_A	-0.02667	-0.7706 to 0.7173	No	ns	>0.9999
cdNV_TGF_C vs. S80_B	-0.43	-1.174 to 0.314	No	ns	0.9095
cdNV_TGF_C vs. S80_C	-0.27	-1.014 to 0.474	No	ns	>0.9999
cdNV_DMEM_C vs. sn_S80_A	0.025	-0.8068 to 0.8568	No	ns	>0.9999
cdNV_DMEM_C vs. sn_S80_B	-0.185	-1.017 to 0.6468	No	ns	>0.9999
cdNV_DMEM_C vs. sn_S80_C	-0.09667	-0.8406 to 0.6473	No	ns	>0.9999
cdNV_DMEM_C vs. sn_RolPA_A	0.01	-0.734 to 0.754	No	ns	>0.9999
cdNV_DMEM_C vs. sn_RolPA_B	-0.08	-0.824 to 0.664	No	ns	>0.9999
cdNV_DMEM_C vs. sn_RolPA_C	0.06	-0.684 to 0.804	No	ns	>0.9999
cdNV_DMEM_C vs. sn_TGF_A	0.03	-0.714 to 0.774	No	ns	>0.9999
cdNV_DMEM_C vs. sn_TGF_B	-0.1	-0.844 to 0.644	No	ns	>0.9999
cdNV_DMEM_C vs. sn_TGF_C	-0.13	-0.874 to 0.614	No	ns	>0.9999
cdNV_DMEM_C vs. sn_DMEM_A	0.02333	-0.7206 to 0.7673	No	ns	>0.9999
cdNV_DMEM_C vs. sn_DMEM_B	-0.01667	-0.7606 to 0.7273	No	ns	>0.9999
cdNV_DMEM_C vs. sn_DMEM_C	-0.1933	-0.9373 to 0.5506	No	ns	>0.9999
cdNV_DMEM_C vs. S80_A	-0.02	-0.764 to 0.724	No	ns	>0.9999
cdNV_DMEM_C vs. S80_B	-0.4233	-1.167 to 0.3206	No	ns	0.9224
cdNV_DMEM_C vs. S80_C	-0.2633	-1.007 to 0.4806	No	ns	>0.9999
sn_S80_A vs. sn_S80_B	-0.21	-1.121 to 0.7012	No	ns	>0.9999
sn_S80_A vs. sn_S80_C	-0.1217	-0.9534 to 0.7101	No	ns	>0.9999
sn_S80_A vs. sn_RolPA_A	-0.015	-0.8468 to 0.8168	No	ns	>0.9999
sn_S80_A vs. sn_RolPA_B	-0.105	-0.9368 to 0.7268	No	ns	>0.9999
sn_S80_A vs. sn_RolPA_C	0.035	-0.7968 to 0.8668	No	ns	>0.9999
sn_S80_A vs. sn_TGF_A	0.005	-0.8268 to 0.8368	No	ns	>0.9999
sn_S80_A vs. sn_TGF_B	-0.125	-0.9568 to 0.7068	No	ns	>0.9999
sn_S80_A vs. sn_TGF_C	-0.155	-0.9868 to 0.6768	No	ns	>0.9999
sn_S80_A vs. sn_DMEM_A	-0.001667	-0.8334 to 0.8301	No	ns	>0.9999
sn_S80_A vs. sn_DMEM_B	-0.04167	-0.8734 to 0.7901	No	ns	>0.9999
sn_S80_A vs. sn_DMEM_C	-0.2183	-1.05 to 0.6134	No	ns	>0.9999
sn_S80_A vs. S80_A	-0.045	-0.8768 to 0.7868	No	ns	>0.9999
sn_S80_A vs. S80_B	-0.4483	-1.28 to 0.3834	No	ns	0.9563
sn_S80_A vs. S80_C	-0.2883	-1.12 to 0.5434	No	ns	>0.9999
sn_S80_B vs. sn_S80_C	0.08833	-0.7434 to 0.9201	No	ns	>0.9999
sn_S80_B vs. sn_RolPA_A	0.195	-0.6368 to 1.027	No	ns	>0.9999
sn_S80_B vs. sn_RolPA_B	0.105	-0.7268 to 0.9368	No	ns	>0.9999
sn_S80_B vs. sn_RolPA_C	0.245	-0.5868 to 1.077	No	ns	>0.9999
sn_S80_B vs. sn_TGF_A	0.215	-0.6168 to 1.047	No	ns	>0.9999
sn_S80_B vs. sn_TGF_B	0.085	-0.7468 to 0.9168	No	ns	>0.9999
sn_S80_B vs. sn_TGF_C	0.055	-0.7768 to 0.8868	No	ns	>0.9999
sn_S80_B vs. sn_DMEM_A	0.2083	-0.6234 to 1.04	No	ns	>0.9999
sn_S80_B vs. sn_DMEM_B	0.1683	-0.6634 to 1	No	ns	>0.9999

sn_S80_B vs. sn_DMEM_C	-0.008333	-0.8401 to 0.8234	No	ns	>0.9999
sn_S80_B vs. S80_A	0.165	-0.6668 to 0.9968	No	ns	>0.9999
sn_S80_B vs. S80_B	-0.2383	-1.07 to 0.5934	No	ns	>0.9999
sn_S80_B vs. S80_C	-0.07833	-0.9101 to 0.7534	No	ns	>0.9999
sn_S80_C vs. sn_RolPA_A	0.1067	-0.6373 to 0.8506	No	ns	>0.9999
sn_S80_C vs. sn_RolPA_B	0.01667	-0.7273 to 0.7606	No	ns	>0.9999
sn_S80_C vs. sn_RolPA_C	0.1567	-0.5873 to 0.9006	No	ns	>0.9999
sn_S80_C vs. sn_TGF_A	0.1267	-0.6173 to 0.8706	No	ns	>0.9999
sn_S80_C vs. sn_TGF_B	-0.003333	-0.7473 to 0.7406	No	ns	>0.9999
sn_S80_C vs. sn_TGF_C	-0.03333	-0.7773 to 0.7106	No	ns	>0.9999
sn_S80_C vs. sn_DMEM_A	0.12	-0.624 to 0.864	No	ns	>0.9999
sn_S80_C vs. sn_DMEM_B	0.08	-0.664 to 0.824	No	ns	>0.9999
sn_S80_C vs. sn_DMEM_C	-0.09667	-0.8406 to 0.6473	No	ns	>0.9999
sn_S80_C vs. S80_A	0.07667	-0.6673 to 0.8206	No	ns	>0.9999
sn_S80_C vs. S80_B	-0.3267	-1.071 to 0.4173	No	ns	0.9973
sn_S80_C vs. S80_C	-0.1667	-0.9106 to 0.5773	No	ns	>0.9999
sn_RolPA_A vs. sn_RolPA_B	-0.09	-0.834 to 0.654	No	ns	>0.9999
sn_RolPA_A vs. sn_RolPA_C	0.05	-0.694 to 0.794	No	ns	>0.9999
sn_RolPA_A vs. sn_TGF_A	0.02	-0.724 to 0.764	No	ns	>0.9999
sn_RolPA_A vs. sn_TGF_B	-0.11	-0.854 to 0.634	No	ns	>0.9999
sn_RolPA_A vs. sn_TGF_C	-0.14	-0.884 to 0.604	No	ns	>0.9999
sn_RolPA_A vs. sn_DMEM_A	0.01333	-0.7306 to 0.7573	No	ns	>0.9999
sn_RolPA_A vs. sn_DMEM_B	-0.02667	-0.7706 to 0.7173	No	ns	>0.9999
sn_RolPA_A vs. sn_DMEM_C	-0.2033	-0.9473 to 0.5406	No	ns	>0.9999
sn_RolPA_A vs. S80_A	-0.03	-0.774 to 0.714	No	ns	>0.9999
sn_RolPA_A vs. S80_B	-0.4333	-1.177 to 0.3106	No	ns	0.9026
sn_RolPA_A vs. S80_C	-0.2733	-1.017 to 0.4706	No	ns	0.9999
sn_RolPA_B vs. sn_RolPA_C	0.14	-0.604 to 0.884	No	ns	>0.9999
sn_RolPA_B vs. sn_TGF_A	0.11	-0.634 to 0.854	No	ns	>0.9999
sn_RolPA_B vs. sn_TGF_B	-0.02	-0.764 to 0.724	No	ns	>0.9999
sn_RolPA_B vs. sn_TGF_C	-0.05	-0.794 to 0.694	No	ns	>0.9999
sn_RolPA_B vs. sn_DMEM_A	0.1033	-0.6406 to 0.8473	No	ns	>0.9999
sn_RolPA_B vs. sn_DMEM_B	0.06333	-0.6806 to 0.8073	No	ns	>0.9999
sn_RolPA_B vs. sn_DMEM_C	-0.1133	-0.8573 to 0.6306	No	ns	>0.9999
sn_RolPA_B vs. S80_A	0.06	-0.684 to 0.804	No	ns	>0.9999
sn_RolPA_B vs. S80_B	-0.3433	-1.087 to 0.4006	No	ns	0.9942
sn_RolPA_B vs. S80_C	-0.1833	-0.9273 to 0.5606	No	ns	>0.9999
sn_RolPA_C vs. sn_TGF_A	-0.03	-0.774 to 0.714	No	ns	>0.9999
sn_RolPA_C vs. sn_TGF_B	-0.16	-0.904 to 0.584	No	ns	>0.9999
sn_RolPA_C vs. sn_TGF_C	-0.19	-0.934 to 0.554	No	ns	>0.9999
sn_RolPA_C vs. sn_DMEM_A	-0.03667	-0.7806 to 0.7073	No	ns	>0.9999
sn_RolPA_C vs. sn_DMEM_B	-0.07667	-0.8206 to 0.6673	No	ns	>0.9999
sn_RolPA_C vs. sn_DMEM_C	-0.2533	-0.9973 to 0.4906	No	ns	>0.9999
sn_RolPA_C vs. S80_A	-0.08	-0.824 to 0.664	No	ns	>0.9999
sn_RolPA_C vs. S80_B	-0.4833	-1.227 to 0.2606	No	ns	0.7612

sn_RolPA_C vs. S80_C	-0.3233	-1.067 to 0.4206	No	ns	0.9977
sn_TGF_A vs. sn_TGF_B	-0.13	-0.874 to 0.614	No	ns	>0.9999
sn_TGF_A vs. sn_TGF_C	-0.16	-0.904 to 0.584	No	ns	>0.9999
sn_TGF_A vs. sn_DMEM_A	-0.006667	-0.7506 to 0.7373	No	ns	>0.9999
sn_TGF_A vs. sn_DMEM_B	-0.04667	-0.7906 to 0.6973	No	ns	>0.9999
sn_TGF_A vs. sn_DMEM_C	-0.2233	-0.9673 to 0.5206	No	ns	>0.9999
sn_TGF_A vs. S80_A	-0.05	-0.794 to 0.694	No	ns	>0.9999
sn_TGF_A vs. S80_B	-0.4533	-1.197 to 0.2906	No	ns	0.8543
sn_TGF_A vs. S80_C	-0.2933	-1.037 to 0.4506	No	ns	0.9996
sn_TGF_B vs. sn_TGF_C	-0.03	-0.774 to 0.714	No	ns	>0.9999
sn_TGF_B vs. sn_DMEM_A	0.1233	-0.6206 to 0.8673	No	ns	>0.9999
sn_TGF_B vs. sn_DMEM_B	0.08333	-0.6606 to 0.8273	No	ns	>0.9999
sn_TGF_B vs. sn_DMEM_C	-0.09333	-0.8373 to 0.6506	No	ns	>0.9999
sn_TGF_B vs. S80_A	0.08	-0.664 to 0.824	No	ns	>0.9999
sn_TGF_B vs. S80_B	-0.3233	-1.067 to 0.4206	No	ns	0.9977
sn_TGF_B vs. S80_C	-0.1633	-0.9073 to 0.5806	No	ns	>0.9999
sn_TGF_C vs. sn_DMEM_A	0.1533	-0.5906 to 0.8973	No	ns	>0.9999
sn_TGF_C vs. sn_DMEM_B	0.1133	-0.6306 to 0.8573	No	ns	>0.9999
sn_TGF_C vs. sn_DMEM_C	-0.06333	-0.8073 to 0.6806	No	ns	>0.9999
sn_TGF_C vs. S80_A	0.11	-0.634 to 0.854	No	ns	>0.9999
sn_TGF_C vs. S80_B	-0.2933	-1.037 to 0.4506	No	ns	0.9996
sn_TGF_C vs. S80_C	-0.1333	-0.8773 to 0.6106	No	ns	>0.9999
sn_DMEM_A vs. sn_DMEM_B	-0.04	-0.784 to 0.704	No	ns	>0.9999
sn_DMEM_A vs. sn_DMEM_C	-0.2167	-0.9606 to 0.5273	No	ns	>0.9999
sn_DMEM_A vs. S80_A	-0.04333	-0.7873 to 0.7006	No	ns	>0.9999
sn_DMEM_A vs. S80_B	-0.4467	-1.191 to 0.2973	No	ns	0.8717
sn_DMEM_A vs. S80_C	-0.2867	-1.031 to 0.4573	No	ns	0.9997
sn_DMEM_B vs. sn_DMEM_C	-0.1767	-0.9206 to 0.5673	No	ns	>0.9999
sn_DMEM_B vs. S80_A	-0.003333	-0.7473 to 0.7406	No	ns	>0.9999
sn_DMEM_B vs. S80_B	-0.4067	-1.151 to 0.3373	No	ns	0.949
sn_DMEM_B vs. S80_C	-0.2467	-0.9906 to 0.4973	No	ns	>0.9999
sn_DMEM_C vs. S80_A	0.1733	-0.5706 to 0.9173	No	ns	>0.9999
sn_DMEM_C vs. S80_B	-0.23	-0.974 to 0.514	No	ns	>0.9999
sn_DMEM_C vs. S80_C	-0.07	-0.814 to 0.674	No	ns	>0.9999
S80_A vs. S80_B	-0.4033	-1.147 to 0.3406	No	ns	0.9534
S80_A vs. S80_C	-0.2433	-0.9873 to 0.5006	No	ns	>0.9999
S80_B vs. S80_C	0.16	-0.584 to 0.904	No	ns	>0.9999

Table S9. Statistical analysis.
Results of one-way ANOVA test with a post-hoc Tukey test of PLIN2 mRNA transcription in Figure 4d and Figure S5.

Number of families	1				
Number of comparisons per family	528				
Alpha	0.05				
**Tukey's multiple comparisons test**	**Mean Diff.**	**95.00% CI of diff.**	**Significant ?**	**Summary**	**Adjusted P Value**
DMEM vs. RolPA	-1.687	-2.291 to -1.083	Yes	****	<0.0001
DMEM vs. TGF-ß1	-0.09267	-0.6591 to 0.4738	No	ns	>0.9999
DMEM vs. S80	-1.782	-2.386 to -1.178	Yes	****	<0.0001
DMEM vs. PBS	-0.07467	-0.6411 to 0.4918	No	ns	>0.9999
DMEM vs. HEPES	-0.2042	-0.808 to 0.3997	No	ns	>0.9999
DMEM vs. cdNV_S80_A	-0.59	-1.251 to 0.07148	No	ns	0.1555
DMEM vs. cdNV_RolPA_A	-0.34	-1.001 to 0.3215	No	ns	0.9756
DMEM vs. cdNV_TGF_A	-0.08333	-0.7448 to 0.5781	No	ns	>0.9999
DMEM vs. cdNV_DMEM_A	-0.4767	-1.138 to 0.1848	No	ns	0.5615
DMEM vs. cdNV_S80_B	-0.3433	-1.005 to 0.3181	No	ns	0.9725
DMEM vs. cdNV_RolPA_B	-0.1433	-0.8048 to 0.5181	No	ns	>0.9999
DMEM vs. cdNV_TGF_B	-0.05	-0.7115 to 0.6115	No	ns	>0.9999
DMEM vs. cdNV_DMEM_B	-0.39	-1.051 to 0.2715	No	ns	0.8925
DMEM vs. cdNV_S80_C	-0.16	-0.8215 to 0.5015	No	ns	>0.9999
DMEM vs. cdNV_RolPA_C	0.08333	-0.5781 to 0.7448	No	ns	>0.9999
DMEM vs. cdNV_TGF_C	0.02667	-0.6348 to 0.6881	No	ns	>0.9999
DMEM vs. cdNV_DMEM_C	-0.11	-0.7715 to 0.5515	No	ns	>0.9999
DMEM vs. sn_S80_A	0.03667	-0.6248 to 0.6981	No	ns	>0.9999
DMEM vs. sn_S80_B	0.1233	-0.5381 to 0.7848	No	ns	>0.9999
DMEM vs. sn_S80_C	0.06667	-0.5948 to 0.7281	No	ns	>0.9999
DMEM vs. sn_RolPA_A	0.07667	-0.5848 to 0.7381	No	ns	>0.9999
DMEM vs. sn_RolPA_B	0.05667	-0.6048 to 0.7181	No	ns	>0.9999
DMEM vs. sn_RolPA_C	0.1633	-0.4981 to 0.8248	No	ns	>0.9999
DMEM vs. sn_TGF_A	0.1533	-0.5081 to 0.8148	No	ns	>0.9999
DMEM vs. sn_TGF_B	0.3483	-0.4155 to 1.112	No	ns	0.9953
DMEM vs. sn_TGF_C	0.22	-0.4415 to 0.8815	No	ns	>0.9999
DMEM vs. sn_DMEM_A	0.08	-0.5815 to 0.7415	No	ns	>0.9999
DMEM vs. sn_DMEM_B	0.11	-0.5515 to 0.7715	No	ns	>0.9999
DMEM vs. sn_DMEM_C	-0.02333	-0.6848 to 0.6381	No	ns	>0.9999
DMEM vs. S80_A	-0.3567	-1.018 to 0.3048	No	ns	0.957

DMEM vs. S80_B	-0.25	-0.9115 to 0.4115	No	ns	0.9998
DMEM vs. S80_C	-0.06667	-0.7281 to 0.5948	No	ns	>0.9999
RolPA vs. TGF-ß1	1.594	0.9665 to 2.222	Yes	****	<0.0001
RolPA vs. S80	-0.095	-0.7565 to 0.5665	No	ns	>0.9999
RolPA vs. PBS	1.612	0.9845 to 2.24	Yes	****	<0.0001
RolPA vs. HEPES	1.483	0.821 to 2.144	Yes	****	<0.0001
RolPA vs. cdNV_S80_A	1.097	0.3822 to 1.811	Yes	****	<0.0001
RolPA vs. cdNV_RolPA_A	1.347	0.6322 to 2.061	Yes	****	<0.0001
RolPA vs. cdNV_TGF_A	1.603	0.8889 to 2.318	Yes	****	<0.0001
RolPA vs. cdNV_DMEM_A	1.21	0.4955 to 1.924	Yes	****	<0.0001
RolPA vs. cdNV_S80_B	1.343	0.6289 to 2.058	Yes	****	<0.0001
RolPA vs. cdNV_RolPA_B	1.543	0.8289 to 2.258	Yes	****	<0.0001
RolPA vs. cdNV_TGF_B	1.637	0.9222 to 2.351	Yes	****	<0.0001
RolPA vs. cdNV_DMEM_B	1.297	0.5822 to 2.011	Yes	****	<0.0001
RolPA vs. cdNV_S80_C	1.527	0.8122 to 2.241	Yes	****	<0.0001
RolPA vs. cdNV_RolPA_C	1.77	1.056 to 2.484	Yes	****	<0.0001
RolPA vs. cdNV_TGF_C	1.713	0.9989 to 2.428	Yes	****	<0.0001
RolPA vs. cdNV_DMEM_C	1.577	0.8622 to 2.291	Yes	****	<0.0001
RolPA vs. sn_S80_A	1.723	1.009 to 2.438	Yes	****	<0.0001
RolPA vs. sn_S80_B	1.81	1.096 to 2.524	Yes	****	<0.0001
RolPA vs. sn_S80_C	1.753	1.039 to 2.468	Yes	****	<0.0001
RolPA vs. sn_RolPA_A	1.763	1.049 to 2.478	Yes	****	<0.0001
RolPA vs. sn_RolPA_B	1.743	1.029 to 2.458	Yes	****	<0.0001
RolPA vs. sn_RolPA_C	1.85	1.136 to 2.564	Yes	****	<0.0001
RolPA vs. sn_TGF_A	1.84	1.126 to 2.554	Yes	****	<0.0001
RolPA vs. sn_TGF_B	2.035	1.225 to 2.845	Yes	****	<0.0001
RolPA vs. sn_TGF_C	1.907	1.192 to 2.621	Yes	****	<0.0001
RolPA vs. sn_DMEM_A	1.767	1.052 to 2.481	Yes	****	<0.0001
RolPA vs. sn_DMEM_B	1.797	1.082 to 2.511	Yes	****	<0.0001
RolPA vs. sn_DMEM_C	1.663	0.9489 to 2.378	Yes	****	<0.0001
RolPA vs. S80_A	1.33	0.6155 to 2.044	Yes	****	<0.0001
RolPA vs. S80_B	1.437	0.7222 to 2.151	Yes	****	<0.0001
RolPA vs. S80_C	1.62	0.9055 to 2.334	Yes	****	<0.0001
TGF-ß1 vs. S80	-1.689	-2.317 to -1.061	Yes	****	<0.0001
TGF-ß1 vs. PBS	0.018	-0.5736 to 0.6096	No	ns	>0.9999
TGF-ß1 vs. HEPES	-0.1115	-0.739 to 0.516	No	ns	>0.9999
TGF-ß1 vs. cdNV_S80_A	-0.4973	-1.181 to 0.1858	No	ns	0.5395
TGF-ß1 vs. cdNV_RolPA_A	-0.2473	-0.9305 to 0.4358	No	ns	>0.9999
TGF-ß1 vs. cdNV_TGF_A	0.009333	-0.6738 to 0.6925	No	ns	>0.9999
TGF-ß1 vs. cdNV_DMEM_A	-0.384	-1.067 to 0.2992	No	ns	0.9326
TGF-ß1 vs. cdNV_S80_B	-0.2507	-0.9338 to 0.4325	No	ns	0.9999
TGF-ß1 vs. cdNV_RolPA_B	-0.05067	-0.7338 to 0.6325	No	ns	>0.9999
TGF-ß1 vs. cdNV_TGF_B	0.04267	-0.6405 to 0.7258	No	ns	>0.9999

TGF-ß1 vs. cdNV_DMEM_B	-0.2973	-0.9805 to 0.3858	No	ns	0.9977
TGF-ß1 vs. cdNV_S80_C	-0.06733	-0.7505 to 0.6158	No	ns	>0.9999
TGF-ß1 vs. cdNV_RolPA_C	0.176	-0.5072 to 0.8592	No	ns	>0.9999
TGF-ß1 vs. cdNV_TGF_C	0.1193	-0.5638 to 0.8025	No	ns	>0.9999
TGF-ß1 vs. cdNV_DMEM_C	-0.01733	-0.7005 to 0.6658	No	ns	>0.9999
TGF-ß1 vs. sn_S80_A	0.1293	-0.5538 to 0.8125	No	ns	>0.9999
TGF-ß1 vs. sn_S80_B	0.216	-0.4672 to 0.8992	No	ns	>0.9999
TGF-ß1 vs. sn_S80_C	0.1593	-0.5238 to 0.8425	No	ns	>0.9999
TGF-ß1 vs. sn_RolPA_A	0.1693	-0.5138 to 0.8525	No	ns	>0.9999
TGF-ß1 vs. sn_RolPA_B	0.1493	-0.5338 to 0.8325	No	ns	>0.9999
TGF-ß1 vs. sn_RolPA_C	0.256	-0.4272 to 0.9392	No	ns	0.9998
TGF-ß1 vs. sn_TGF_A	0.246	-0.4372 to 0.9292	No	ns	>0.9999
TGF-ß1 vs. sn_TGF_B	0.441	-0.3417 to 1.224	No	ns	0.9309
TGF-ß1 vs. sn_TGF_C	0.3127	-0.3705 to 0.9958	No	ns	0.995
TGF-ß1 vs. sn_DMEM_A	0.1727	-0.5105 to 0.8558	No	ns	>0.9999
TGF-ß1 vs. sn_DMEM_B	0.2027	-0.4805 to 0.8858	No	ns	>0.9999
TGF-ß1 vs. sn_DMEM_C	0.06933	-0.6138 to 0.7525	No	ns	>0.9999
TGF-ß1 vs. S80_A	-0.264	-0.9472 to 0.4192	No	ns	0.9997
TGF-ß1 vs. S80_B	-0.1573	-0.8405 to 0.5258	No	ns	>0.9999
TGF-ß1 vs. S80_C	0.026	-0.6572 to 0.7092	No	ns	>0.9999
S80 vs. PBS	1.707	1.079 to 2.335	Yes	****	<0.0001
S80 vs. HEPES	1.578	0.916 to 2.239	Yes	****	<0.0001
S80 vs. cdNV_S80_A	1.192	0.4772 to 1.906	Yes	****	<0.0001
S80 vs. cdNV_RolPA_A	1.442	0.7272 to 2.156	Yes	****	<0.0001
S80 vs. cdNV_TGF_A	1.698	0.9839 to 2.413	Yes	****	<0.0001
S80 vs. cdNV_DMEM_A	1.305	0.5905 to 2.019	Yes	****	<0.0001
S80 vs. cdNV_S80_B	1.438	0.7239 to 2.153	Yes	****	<0.0001
S80 vs. cdNV_RolPA_B	1.638	0.9239 to 2.353	Yes	****	<0.0001
S80 vs. cdNV_TGF_B	1.732	1.017 to 2.446	Yes	****	<0.0001
S80 vs. cdNV_DMEM_B	1.392	0.6772 to 2.106	Yes	****	<0.0001
S80 vs. cdNV_S80_C	1.622	0.9072 to 2.336	Yes	****	<0.0001
S80 vs. cdNV_RolPA_C	1.865	1.151 to 2.579	Yes	****	<0.0001
S80 vs. cdNV_TGF_C	1.808	1.094 to 2.523	Yes	****	<0.0001
S80 vs. cdNV_DMEM_C	1.672	0.9572 to 2.386	Yes	****	<0.0001
S80 vs. sn_S80_A	1.818	1.104 to 2.533	Yes	****	<0.0001
S80 vs. sn_S80_B	1.905	1.191 to 2.619	Yes	****	<0.0001
S80 vs. sn_S80_C	1.848	1.134 to 2.563	Yes	****	<0.0001
S80 vs. sn_RolPA_A	1.858	1.144 to 2.573	Yes	****	<0.0001
S80 vs. sn_RolPA_B	1.838	1.124 to 2.553	Yes	****	<0.0001
S80 vs. sn_RolPA_C	1.945	1.231 to 2.659	Yes	****	<0.0001
S80 vs. sn_TGF_A	1.935	1.221 to 2.649	Yes	****	<0.0001

S80 vs. sn_TGF_B	2.13	1.32 to 2.94	Yes	****	<0.0001
S80 vs. sn_TGF_C	2.002	1.287 to 2.716	Yes	****	<0.0001
S80 vs. sn_DMEM_A	1.862	1.147 to 2.576	Yes	****	<0.0001
S80 vs. sn_DMEM_B	1.892	1.177 to 2.606	Yes	****	<0.0001
S80 vs. sn_DMEM_C	1.758	1.044 to 2.473	Yes	****	<0.0001
S80 vs. S80_A	1.425	0.7105 to 2.139	Yes	****	<0.0001
S80 vs. S80_B	1.532	0.8172 to 2.246	Yes	****	<0.0001
S80 vs. S80_C	1.715	1.001 to 2.429	Yes	****	<0.0001
PBS vs. HEPES	-0.1295	-0.757 to 0.498	No	ns	>0.9999
PBS vs. cdNV_S80_A	-0.5153	-1.199 to 0.1678	No	ns	0.4623
PBS vs. cdNV_RolPA_A	-0.2653	-0.9485 to 0.4178	No	ns	0.9997
PBS vs. cdNV_TGF_A	-0.008667	-0.6918 to 0.6745	No	ns	>0.9999
PBS vs. cdNV_DMEM_A	-0.402	-1.085 to 0.2812	No	ns	0.8944
PBS vs. cdNV_S80_B	-0.2687	-0.9518 to 0.4145	No	ns	0.9996
PBS vs. cdNV_RolPA_B	-0.06867	-0.7518 to 0.6145	No	ns	>0.9999
PBS vs. cdNV_TGF_B	0.02467	-0.6585 to 0.7078	No	ns	>0.9999
PBS vs. cdNV_DMEM_B	-0.3153	-0.9985 to 0.3678	No	ns	0.9943
PBS vs. cdNV_S80_C	-0.08533	-0.7685 to 0.5978	No	ns	>0.9999
PBS vs. cdNV_RolPA_C	0.158	-0.5252 to 0.8412	No	ns	>0.9999
PBS vs. cdNV_TGF_C	0.1013	-0.5818 to 0.7845	No	ns	>0.9999
PBS vs. cdNV_DMEM_C	-0.03533	-0.7185 to 0.6478	No	ns	>0.9999
PBS vs. sn_S80_A	0.1113	-0.5718 to 0.7945	No	ns	>0.9999
PBS vs. sn_S80_B	0.198	-0.4852 to 0.8812	No	ns	>0.9999
PBS vs. sn_S80_C	0.1413	-0.5418 to 0.8245	No	ns	>0.9999
PBS vs. sn_RolPA_A	0.1513	-0.5318 to 0.8345	No	ns	>0.9999
PBS vs. sn_RolPA_B	0.1313	-0.5518 to 0.8145	No	ns	>0.9999
PBS vs. sn_RolPA_C	0.238	-0.4452 to 0.9212	No	ns	>0.9999
PBS vs. sn_TGF_A	0.228	-0.4552 to 0.9112	No	ns	>0.9999
PBS vs. sn_TGF_B	0.423	-0.3597 to 1.206	No	ns	0.9558
PBS vs. sn_TGF_C	0.2947	-0.3885 to 0.9778	No	ns	0.998
PBS vs. sn_DMEM_A	0.1547	-0.5285 to 0.8378	No	ns	>0.9999
PBS vs. sn_DMEM_B	0.1847	-0.4985 to 0.8678	No	ns	>0.9999
PBS vs. sn_DMEM_C	0.05133	-0.6318 to 0.7345	No	ns	>0.9999
PBS vs. S80_A	-0.282	-0.9652 to 0.4012	No	ns	0.9991
PBS vs. S80_B	-0.1753	-0.8585 to 0.5078	No	ns	>0.9999
PBS vs. S80_C	0.008	-0.6752 to 0.6912	No	ns	>0.9999
HEPES vs. cdNV_S80_A	-0.3858	-1.1 to 0.3286	No	ns	0.9562
HEPES vs. cdNV_RolPA_A	-0.1358	-0.8503 to 0.5786	No	ns	>0.9999
HEPES vs. cdNV_TGF_A	0.1208	-0.5936 to 0.8353	No	ns	>0.9999

HEPES vs. cdNV_DMEM_A	-0.2725	-0.987 to 0.442	No	ns	0.9998
HEPES vs. cdNV_S80_B	-0.1392	-0.8536 to 0.5753	No	ns	>0.9999
HEPES vs. cdNV_RolPA_B	0.06083	-0.6536 to 0.7753	No	ns	>0.9999
HEPES vs. cdNV_TGF_B	0.1542	-0.5603 to 0.8686	No	ns	>0.9999
HEPES vs. cdNV_DMEM_B	-0.1858	-0.9003 to 0.5286	No	ns	>0.9999
HEPES vs. cdNV_S80_C	0.04417	-0.6703 to 0.7586	No	ns	>0.9999
HEPES vs. cdNV_RolPA_C	0.2875	-0.427 to 1.002	No	ns	0.9994
HEPES vs. cdNV_TGF_C	0.2308	-0.4836 to 0.9453	No	ns	>0.9999
HEPES vs. cdNV_DMEM_C	0.09417	-0.6203 to 0.8086	No	ns	>0.9999
HEPES vs. sn_S80_A	0.2408	-0.4736 to 0.9553	No	ns	>0.9999
HEPES vs. sn_S80_B	0.3275	-0.387 to 1.042	No	ns	0.9949
HEPES vs. sn_S80_C	0.2708	-0.4436 to 0.9853	No	ns	0.9998
HEPES vs. sn_RolPA_A	0.2808	-0.4336 to 0.9953	No	ns	0.9996
HEPES vs. sn_RolPA_B	0.2608	-0.4536 to 0.9753	No	ns	>0.9999
HEPES vs. sn_RolPA_C	0.3675	-0.347 to 1.082	No	ns	0.9753
HEPES vs. sn_TGF_A	0.3575	-0.357 to 1.072	No	ns	0.9826
HEPES vs. sn_TGF_B	0.5525	-0.2576 to 1.363	No	ns	0.6751
HEPES vs. sn_TGF_C	0.4242	-0.2903 to 1.139	No	ns	0.8855
HEPES vs. sn_DMEM_A	0.2842	-0.4303 to 0.9986	No	ns	0.9995
HEPES vs. sn_DMEM_B	0.3142	-0.4003 to 1.029	No	ns	0.9973
HEPES vs. sn_DMEM_C	0.1808	-0.5336 to 0.8953	No	ns	>0.9999
HEPES vs. S80_A	-0.1525	-0.867 to 0.562	No	ns	>0.9999
HEPES vs. S80_B	-0.04583	-0.7603 to 0.6686	No	ns	>0.9999
HEPES vs. S80_C	0.1375	-0.577 to 0.852	No	ns	>0.9999
cdNV_S80_A vs. cdNV_RolPA_A	0.25	-0.5138 to 1.014	No	ns	>0.9999
cdNV_S80_A vs. cdNV_TGF_A	0.5067	-0.2571 to 1.27	No	ns	0.7272
cdNV_S80_A vs. cdNV_DMEM_A	0.1133	-0.6505 to 0.8771	No	ns	>0.9999
cdNV_S80_A vs. cdNV_S80_B	0.2467	-0.5171 to 1.01	No	ns	>0.9999
cdNV_S80_A vs. cdNV_RolPA_B	0.4467	-0.3171 to 1.21	No	ns	0.9003
cdNV_S80_A vs. cdNV_TGF_B	0.54	-0.2238 to 1.304	No	ns	0.602
cdNV_S80_A vs. cdNV_DMEM_B	0.2	-0.5638 to 0.9638	No	ns	>0.9999
cdNV_S80_A vs. cdNV_S80_C	0.43	-0.3338 to 1.194	No	ns	0.9315
cdNV_S80_A vs. cdNV_RolPA_C	0.6733	-0.09048 to 1.437	No	ns	0.1714
cdNV_S80_A vs. cdNV_TGF_C	0.6167	-0.1471 to 1.38	No	ns	0.3205
cdNV_S80_A vs. cdNV_DMEM_C	0.48	-0.2838 to 1.244	No	ns	0.815
cdNV_S80_A vs. sn_S80_A	0.6267	-0.1371 to 1.39	No	ns	0.2897
cdNV_S80_A vs. sn_S80_B	0.7133	-0.05048 to 1.477	No	ns	0.1026
cdNV_S80_A vs. sn_S80_C	0.6567	-0.1071 to 1.42	No	ns	0.2087
cdNV_S80_A vs. sn_RolPA_A	0.6667	-0.09714 to 1.43	No	ns	0.1856
cdNV_S80_A vs. sn_RolPA_B	0.6467	-0.1171 to 1.41	No	ns	0.2337
cdNV_S80_A vs. sn_RolPA_C	0.7533	-0.01048 to 1.517	No	ns	0.0584

cdNV_S80_A vs. sn_TGF_A	0.7433	-0.02048 to 1.507	No	ns	0.0675
cdNV_S80_A vs. sn_TGF_B	0.9383	0.08437 to 1.792	Yes	*	0.015
cdNV_S80_A vs. sn_TGF_C	0.81	0.04619 to 1.574	Yes	*	0.0244
cdNV_S80_A vs. sn_DMEM_A	0.67	-0.09381 to 1.434	No	ns	0.1784
cdNV_S80_A vs. sn_DMEM_B	0.7	-0.06381 to 1.464	No	ns	0.1225
cdNV_S80_A vs. sn_DMEM_C	0.5667	-0.1971 to 1.33	No	ns	0.4984
cdNV_S80_A vs. S80_A	0.2333	-0.5305 to 0.9971	No	ns	>0.9999
cdNV_S80_A vs. S80_B	0.34	-0.4238 to 1.104	No	ns	0.9967
cdNV_S80_A vs. S80_C	0.5233	-0.2405 to 1.287	No	ns	0.666
cdNV_RolPA_A vs. cdNV_TGF_A	0.2567	-0.5071 to 1.02	No	ns	>0.9999
cdNV_RolPA_A vs. cdNV_DMEM_A	-0.1367	-0.9005 to 0.6271	No	ns	>0.9999
cdNV_RolPA_A vs. cdNV_S80_B	-0.003333	-0.7671 to 0.7605	No	ns	>0.9999
cdNV_RolPA_A vs. cdNV_RolPA_B	0.1967	-0.5671 to 0.9605	No	ns	>0.9999
cdNV_RolPA_A vs. cdNV_TGF_B	0.29	-0.4738 to 1.054	No	ns	0.9998
cdNV_RolPA_A vs. cdNV_DMEM_B	-0.05	-0.8138 to 0.7138	No	ns	>0.9999
cdNV_RolPA_A vs. cdNV_S80_C	0.18	-0.5838 to 0.9438	No	ns	>0.9999
cdNV_RolPA_A vs. cdNV_RolPA_C	0.4233	-0.3405 to 1.187	No	ns	0.9418
cdNV_RolPA_A vs. cdNV_TGF_C	0.3667	-0.3971 to 1.13	No	ns	0.99
cdNV_RolPA_A vs. cdNV_DMEM_C	0.23	-0.5338 to 0.9938	No	ns	>0.9999
cdNV_RolPA_A vs. sn_S80_A	0.3767	-0.3871 to 1.14	No	ns	0.9856
cdNV_RolPA_A vs. sn_S80_B	0.4633	-0.3005 to 1.227	No	ns	0.8615
cdNV_RolPA_A vs. sn_S80_C	0.4067	-0.3571 to 1.17	No	ns	0.9627
cdNV_RolPA_A vs. sn_RolPA_A	0.4167	-0.3471 to 1.18	No	ns	0.951
cdNV_RolPA_A vs. sn_RolPA_B	0.3967	-0.3671 to 1.16	No	ns	0.9723
cdNV_RolPA_A vs. sn_RolPA_C	0.5033	-0.2605 to 1.267	No	ns	0.739
cdNV_RolPA_A vs. sn_TGF_A	0.4933	-0.2705 to 1.257	No	ns	0.773
cdNV_RolPA_A vs. sn_TGF_B	0.6883	-0.1656 to 1.542	No	ns	0.3237
cdNV_RolPA_A vs. sn_TGF_C	0.56	-0.2038 to 1.324	No	ns	0.5241
cdNV_RolPA_A vs. sn_DMEM_A	0.42	-0.3438 to 1.184	No	ns	0.9465
cdNV_RolPA_A vs. sn_DMEM_B	0.45	-0.3138 to 1.214	No	ns	0.8932
cdNV_RolPA_A vs. sn_DMEM_C	0.3167	-0.4471 to 1.08	No	ns	0.999
cdNV_RolPA_A vs. S80_A	-0.01667	-0.7805 to 0.7471	No	ns	>0.9999
cdNV_RolPA_A vs. S80_B	0.09	-0.6738 to 0.8538	No	ns	>0.9999
cdNV_RolPA_A vs. S80_C	0.2733	-0.4905 to 1.037	No	ns	>0.9999
cdNV_TGF_A vs. cdNV_DMEM_A	-0.3933	-1.157 to 0.3705	No	ns	0.975
cdNV_TGF_A vs. cdNV_S80_B	-0.26	-1.024 to 0.5038	No	ns	>0.9999
cdNV_TGF_A vs. cdNV_RolPA_B	-0.06	-0.8238 to 0.7038	No	ns	>0.9999
cdNV_TGF_A vs. cdNV_TGF_B	0.03333	-0.7305 to 0.7971	No	ns	>0.9999
cdNV_TGF_A vs. cdNV_DMEM_B	-0.3067	-1.07 to 0.4571	No	ns	0.9994
cdNV_TGF_A vs. cdNV_S80_C	-0.07667	-0.8405 to 0.6871	No	ns	>0.9999
cdNV_TGF_A vs. cdNV_RolPA_C	0.1667	-0.5971 to 0.9305	No	ns	>0.9999

cdNV_TGF_A vs. cdNV_TGF_C	0.11	-0.6538 to 0.8738	No	ns	>0.9999
cdNV_TGF_A vs. cdNV_DMEM_C	-0.02667	-0.7905 to 0.7371	No	ns	>0.9999
cdNV_TGF_A vs. sn_S80_A	0.12	-0.6438 to 0.8838	No	ns	>0.9999
cdNV_TGF_A vs. sn_S80_B	0.2067	-0.5571 to 0.9705	No	ns	>0.9999
cdNV_TGF_A vs. sn_S80_C	0.15	-0.6138 to 0.9138	No	ns	>0.9999
cdNV_TGF_A vs. sn_RolPA_A	0.16	-0.6038 to 0.9238	No	ns	>0.9999
cdNV_TGF_A vs. sn_RolPA_B	0.14	-0.6238 to 0.9038	No	ns	>0.9999
cdNV_TGF_A vs. sn_RolPA_C	0.2467	-0.5171 to 1.01	No	ns	>0.9999
cdNV_TGF_A vs. sn_TGF_A	0.2367	-0.5271 to 1	No	ns	>0.9999
cdNV_TGF_A vs. sn_TGF_B	0.4317	-0.4223 to 1.286	No	ns	0.9802
cdNV_TGF_A vs. sn_TGF_C	0.3033	-0.4605 to 1.067	No	ns	0.9995
cdNV_TGF_A vs. sn_DMEM_A	0.1633	-0.6005 to 0.9271	No	ns	>0.9999
cdNV_TGF_A vs. sn_DMEM_B	0.1933	-0.5705 to 0.9571	No	ns	>0.9999
cdNV_TGF_A vs. sn_DMEM_C	0.06	-0.7038 to 0.8238	No	ns	>0.9999
cdNV_TGF_A vs. S80_A	-0.2733	-1.037 to 0.4905	No	ns	>0.9999
cdNV_TGF_A vs. S80_B	-0.1667	-0.9305 to 0.5971	No	ns	>0.9999
cdNV_TGF_A vs. S80_C	0.01667	-0.7471 to 0.7805	No	ns	>0.9999
cdNV_DMEM_A vs. cdNV_S80_B	0.1333	-0.6305 to 0.8971	No	ns	>0.9999
cdNV_DMEM_A vs. cdNV_RolPA_B	0.3333	-0.4305 to 1.097	No	ns	0.9976
cdNV_DMEM_A vs. cdNV_TGF_B	0.4267	-0.3371 to 1.19	No	ns	0.9368
cdNV_DMEM_A vs. cdNV_DMEM_B	0.08667	-0.6771 to 0.8505	No	ns	>0.9999
cdNV_DMEM_A vs. cdNV_S80_C	0.3167	-0.4471 to 1.08	No	ns	0.999
cdNV_DMEM_A vs. cdNV_RolPA_C	0.56	-0.2038 to 1.324	No	ns	0.5241
cdNV_DMEM_A vs. cdNV_TGF_C	0.5033	-0.2605 to 1.267	No	ns	0.739
cdNV_DMEM_A vs. cdNV_DMEM_C	0.3667	-0.3971 to 1.13	No	ns	0.99
cdNV_DMEM_A vs. sn_S80_A	0.5133	-0.2505 to 1.277	No	ns	0.7032
cdNV_DMEM_A vs. sn_S80_B	0.6	-0.1638 to 1.364	No	ns	0.3759
cdNV_DMEM_A vs. sn_S80_C	0.5433	-0.2205 to 1.307	No	ns	0.589
cdNV_DMEM_A vs. sn_RolPA_A	0.5533	-0.2105 to 1.317	No	ns	0.55
cdNV_DMEM_A vs. sn_RolPA_B	0.5333	-0.2305 to 1.297	No	ns	0.6278
cdNV_DMEM_A vs. sn_RolPA_C	0.64	-0.1238 to 1.404	No	ns	0.2515
cdNV_DMEM_A vs. sn_TGF_A	0.63	-0.1338 to 1.394	No	ns	0.2798
cdNV_DMEM_A vs. sn_TGF_B	0.825	-0.02897 to 1.679	No	ns	0.073
cdNV_DMEM_A vs. sn_TGF_C	0.6967	-0.06714 to 1.46	No	ns	0.1279
cdNV_DMEM_A vs. sn_DMEM_A	0.5567	-0.2071 to 1.32	No	ns	0.5371
cdNV_DMEM_A vs. sn_DMEM_B	0.5867	-0.1771 to 1.35	No	ns	0.4233
cdNV_DMEM_A vs. sn_DMEM_C	0.4533	-0.3105 to 1.217	No	ns	0.8857
cdNV_DMEM_A vs. S80_A	0.12	-0.6438 to 0.8838	No	ns	>0.9999
cdNV_DMEM_A vs. S80_B	0.2267	-0.5371 to 0.9905	No	ns	>0.9999
cdNV_DMEM_A vs. S80_C	0.41	-0.3538 to 1.174	No	ns	0.9591
cdNV_S80_B vs. cdNV_RolPA_B	0.2	-0.5638 to 0.9638	No	ns	>0.9999

cdNV_S80_B vs. cdNV_TGF_B	0.2933	-0.4705 to 1.057	No	ns	0.9997
cdNV_S80_B vs. cdNV_DMEM_B	-0.04667	-0.8105 to 0.7171	No	ns	>0.9999
cdNV_S80_B vs. cdNV_S80_C	0.1833	-0.5805 to 0.9471	No	ns	>0.9999
cdNV_S80_B vs. cdNV_RolPA_C	0.4267	-0.3371 to 1.19	No	ns	0.9368
cdNV_S80_B vs. cdNV_TGF_C	0.37	-0.3938 to 1.134	No	ns	0.9887
cdNV_S80_B vs. cdNV_DMEM_C	0.2333	-0.5305 to 0.9971	No	ns	>0.9999
cdNV_S80_B vs. sn_S80_A	0.38	-0.3838 to 1.144	No	ns	0.9839
cdNV_S80_B vs. sn_S80_B	0.4667	-0.2971 to 1.23	No	ns	0.8528
cdNV_S80_B vs. sn_S80_C	0.41	-0.3538 to 1.174	No	ns	0.9591
cdNV_S80_B vs. sn_RolPA_A	0.42	-0.3438 to 1.184	No	ns	0.9465
cdNV_S80_B vs. sn_RolPA_B	0.4	-0.3638 to 1.164	No	ns	0.9693
cdNV_S80_B vs. sn_RolPA_C	0.5067	-0.2571 to 1.27	No	ns	0.7272
cdNV_S80_B vs. sn_TGF_A	0.4967	-0.2671 to 1.26	No	ns	0.7618
cdNV_S80_B vs. sn_TGF_B	0.6917	-0.1623 to 1.546	No	ns	0.3143
cdNV_S80_B vs. sn_TGF_C	0.5633	-0.2005 to 1.327	No	ns	0.5112
cdNV_S80_B vs. sn_DMEM_A	0.4233	-0.3405 to 1.187	No	ns	0.9418
cdNV_S80_B vs. sn_DMEM_B	0.4533	-0.3105 to 1.217	No	ns	0.8857
cdNV_S80_B vs. sn_DMEM_C	0.32	-0.4438 to 1.084	No	ns	0.9988
cdNV_S80_B vs. S80_A	-0.01333	-0.7771 to 0.7505	No	ns	>0.9999
cdNV_S80_B vs. S80_B	0.09333	-0.6705 to 0.8571	No	ns	>0.9999
cdNV_S80_B vs. S80_C	0.2767	-0.4871 to 1.04	No	ns	>0.9999
cdNV_RolPA_B vs. cdNV_TGF_B	0.09333	-0.6705 to 0.8571	No	ns	>0.9999
cdNV_RolPA_B vs. cdNV_DMEM_B	-0.2467	-1.01 to 0.5171	No	ns	>0.9999
cdNV_RolPA_B vs. cdNV_S80_C	-0.01667	-0.7805 to 0.7471	No	ns	>0.9999
cdNV_RolPA_B vs. cdNV_RolPA_C	0.2267	-0.5371 to 0.9905	No	ns	>0.9999
cdNV_RolPA_B vs. cdNV_TGF_C	0.17	-0.5938 to 0.9338	No	ns	>0.9999
cdNV_RolPA_B vs. cdNV_DMEM_C	0.03333	-0.7305 to 0.7971	No	ns	>0.9999
cdNV_RolPA_B vs. sn_S80_A	0.18	-0.5838 to 0.9438	No	ns	>0.9999
cdNV_RolPA_B vs. sn_S80_B	0.2667	-0.4971 to 1.03	No	ns	>0.9999
cdNV_RolPA_B vs. sn_S80_C	0.21	-0.5538 to 0.9738	No	ns	>0.9999
cdNV_RolPA_B vs. sn_RolPA_A	0.22	-0.5438 to 0.9838	No	ns	>0.9999
cdNV_RolPA_B vs. sn_RolPA_B	0.2	-0.5638 to 0.9638	No	ns	>0.9999
cdNV_RolPA_B vs. sn_RolPA_C	0.3067	-0.4571 to 1.07	No	ns	0.9994
cdNV_RolPA_B vs. sn_TGF_A	0.2967	-0.4671 to 1.06	No	ns	0.9997
cdNV_RolPA_B vs. sn_TGF_B	0.4917	-0.3623 to 1.346	No	ns	0.9142
cdNV_RolPA_B vs. sn_TGF_C	0.3633	-0.4005 to 1.127	No	ns	0.9912
cdNV_RolPA_B vs. sn_DMEM_A	0.2233	-0.5405 to 0.9871	No	ns	>0.9999
cdNV_RolPA_B vs. sn_DMEM_B	0.2533	-0.5105 to 1.017	No	ns	>0.9999
cdNV_RolPA_B vs. sn_DMEM_C	0.12	-0.6438 to 0.8838	No	ns	>0.9999
cdNV_RolPA_B vs. S80_A	-0.2133	-0.9771 to 0.5505	No	ns	>0.9999
cdNV_RolPA_B vs. S80_B	-0.1067	-0.8705 to 0.6571	No	ns	>0.9999

cdNV_RolPA_B vs. S80_C	0.07667	-0.6871 to 0.8405	No	ns	>0.9999
cdNV_TGF_B vs. cdNV_DMEM_B	-0.34	-1.104 to 0.4238	No	ns	0.9967
cdNV_TGF_B vs. cdNV_S80_C	-0.11	-0.8738 to 0.6538	No	ns	>0.9999
cdNV_TGF_B vs. cdNV_RolPA_C	0.1333	-0.6305 to 0.8971	No	ns	>0.9999
cdNV_TGF_B vs. cdNV_TGF_C	0.07667	-0.6871 to 0.8405	No	ns	>0.9999
cdNV_TGF_B vs. cdNV_DMEM_C	-0.06	-0.8238 to 0.7038	No	ns	>0.9999
cdNV_TGF_B vs. sn_S80_A	0.08667	-0.6771 to 0.8505	No	ns	>0.9999
cdNV_TGF_B vs. sn_S80_B	0.1733	-0.5905 to 0.9371	No	ns	>0.9999
cdNV_TGF_B vs. sn_S80_C	0.1167	-0.6471 to 0.8805	No	ns	>0.9999
cdNV_TGF_B vs. sn_RolPA_A	0.1267	-0.6371 to 0.8905	No	ns	>0.9999
cdNV_TGF_B vs. sn_RolPA_B	0.1067	-0.6571 to 0.8705	No	ns	>0.9999
cdNV_TGF_B vs. sn_RolPA_C	0.2133	-0.5505 to 0.9771	No	ns	>0.9999
cdNV_TGF_B vs. sn_TGF_A	0.2033	-0.5605 to 0.9671	No	ns	>0.9999
cdNV_TGF_B vs. sn_TGF_B	0.3983	-0.4556 to 1.252	No	ns	0.9934
cdNV_TGF_B vs. sn_TGF_C	0.27	-0.4938 to 1.034	No	ns	>0.9999
cdNV_TGF_B vs. sn_DMEM_A	0.13	-0.6338 to 0.8938	No	ns	>0.9999
cdNV_TGF_B vs. sn_DMEM_B	0.16	-0.6038 to 0.9238	No	ns	>0.9999
cdNV_TGF_B vs. sn_DMEM_C	0.02667	-0.7371 to 0.7905	No	ns	>0.9999
cdNV_TGF_B vs. S80_A	-0.3067	-1.07 to 0.4571	No	ns	0.9994
cdNV_TGF_B vs. S80_B	-0.2	-0.9638 to 0.5638	No	ns	>0.9999
cdNV_TGF_B vs. S80_C	-0.01667	-0.7805 to 0.7471	No	ns	>0.9999
cdNV_DMEM_B vs. cdNV_S80_C	0.23	-0.5338 to 0.9938	No	ns	>0.9999
cdNV_DMEM_B vs. cdNV_RolPA_C	0.4733	-0.2905 to 1.237	No	ns	0.8345
cdNV_DMEM_B vs. cdNV_TGF_C	0.4167	-0.3471 to 1.18	No	ns	0.951
cdNV_DMEM_B vs. cdNV_DMEM_C	0.28	-0.4838 to 1.044	No	ns	0.9999
cdNV_DMEM_B vs. sn_S80_A	0.4267	-0.3371 to 1.19	No	ns	0.9368
cdNV_DMEM_B vs. sn_S80_B	0.5133	-0.2505 to 1.277	No	ns	0.7032
cdNV_DMEM_B vs. sn_S80_C	0.4567	-0.3071 to 1.22	No	ns	0.8779
cdNV_DMEM_B vs. sn_RolPA_A	0.4667	-0.2971 to 1.23	No	ns	0.8528
cdNV_DMEM_B vs. sn_RolPA_B	0.4467	-0.3171 to 1.21	No	ns	0.9003
cdNV_DMEM_B vs. sn_RolPA_C	0.5533	-0.2105 to 1.317	No	ns	0.55
cdNV_DMEM_B vs. sn_TGF_A	0.5433	-0.2205 to 1.307	No	ns	0.589
cdNV_DMEM_B vs. sn_TGF_B	0.7383	-0.1156 to 1.592	No	ns	0.1999
cdNV_DMEM_B vs. sn_TGF_C	0.61	-0.1538 to 1.374	No	ns	0.3421
cdNV_DMEM_B vs. sn_DMEM_A	0.47	-0.2938 to 1.234	No	ns	0.8438
cdNV_DMEM_B vs. sn_DMEM_B	0.5	-0.2638 to 1.264	No	ns	0.7505
cdNV_DMEM_B vs. sn_DMEM_C	0.3667	-0.3971 to 1.13	No	ns	0.99
cdNV_DMEM_B vs. S80_A	0.03333	-0.7305 to 0.7971	No	ns	>0.9999
cdNV_DMEM_B vs. S80_B	0.14	-0.6238 to 0.9038	No	ns	>0.9999
cdNV_DMEM_B vs. S80_C	0.3233	-0.4405 to 1.087	No	ns	0.9985
cdNV_S80_C vs. cdNV_RolPA_C	0.2433	-0.5205 to 1.007	No	ns	>0.9999

cdNV_S80_C vs. cdNV_TGF_C	0.1867	-0.5771 to 0.9505	No	ns	>0.9999
cdNV_S80_C vs. cdNV_DMEM_C	0.05	-0.7138 to 0.8138	No	ns	>0.9999
cdNV_S80_C vs. sn_S80_A	0.1967	-0.5671 to 0.9605	No	ns	>0.9999
cdNV_S80_C vs. sn_S80_B	0.2833	-0.4805 to 1.047	No	ns	0.9999
cdNV_S80_C vs. sn_S80_C	0.2267	-0.5371 to 0.9905	No	ns	>0.9999
cdNV_S80_C vs. sn_RolPA_A	0.2367	-0.5271 to 1	No	ns	>0.9999
cdNV_S80_C vs. sn_RolPA_B	0.2167	-0.5471 to 0.9805	No	ns	>0.9999
cdNV_S80_C vs. sn_RolPA_C	0.3233	-0.4405 to 1.087	No	ns	0.9985
cdNV_S80_C vs. sn_TGF_A	0.3133	-0.4505 to 1.077	No	ns	0.9991
cdNV_S80_C vs. sn_TGF_B	0.5083	-0.3456 to 1.362	No	ns	0.8827
cdNV_S80_C vs. sn_TGF_C	0.38	-0.3838 to 1.144	No	ns	0.9839
cdNV_S80_C vs. sn_DMEM_A	0.24	-0.5238 to 1.004	No	ns	>0.9999
cdNV_S80_C vs. sn_DMEM_B	0.27	-0.4938 to 1.034	No	ns	>0.9999
cdNV_S80_C vs. sn_DMEM_C	0.1367	-0.6271 to 0.9005	No	ns	>0.9999
cdNV_S80_C vs. S80_A	-0.1967	-0.9605 to 0.5671	No	ns	>0.9999
cdNV_S80_C vs. S80_B	-0.09	-0.8538 to 0.6738	No	ns	>0.9999
cdNV_S80_C vs. S80_C	0.09333	-0.6705 to 0.8571	No	ns	>0.9999
cdNV_RolPA_C vs. cdNV_TGF_C	-0.05667	-0.8205 to 0.7071	No	ns	>0.9999
cdNV_RolPA_C vs. cdNV_DMEM_C	-0.1933	-0.9571 to 0.5705	No	ns	>0.9999
cdNV_RolPA_C vs. sn_S80_A	-0.04667	-0.8105 to 0.7171	No	ns	>0.9999
cdNV_RolPA_C vs. sn_S80_B	0.04	-0.7238 to 0.8038	No	ns	>0.9999
cdNV_RolPA_C vs. sn_S80_C	-0.01667	-0.7805 to 0.7471	No	ns	>0.9999
cdNV_RolPA_C vs. sn_RolPA_A	-0.006667	-0.7705 to 0.7571	No	ns	>0.9999
cdNV_RolPA_C vs. sn_RolPA_B	-0.02667	-0.7905 to 0.7371	No	ns	>0.9999
cdNV_RolPA_C vs. sn_RolPA_C	0.08	-0.6838 to 0.8438	No	ns	>0.9999
cdNV_RolPA_C vs. sn_TGF_A	0.07	-0.6938 to 0.8338	No	ns	>0.9999
cdNV_RolPA_C vs. sn_TGF_B	0.265	-0.589 to 1.119	No	ns	>0.9999
cdNV_RolPA_C vs. sn_TGF_C	0.1367	-0.6271 to 0.9005	No	ns	>0.9999
cdNV_RolPA_C vs. sn_DMEM_A	-0.003333	-0.7671 to 0.7605	No	ns	>0.9999
cdNV_RolPA_C vs. sn_DMEM_B	0.02667	-0.7371 to 0.7905	No	ns	>0.9999
cdNV_RolPA_C vs. sn_DMEM_C	-0.1067	-0.8705 to 0.6571	No	ns	>0.9999
cdNV_RolPA_C vs. S80_A	-0.44	-1.204 to 0.3238	No	ns	0.9137
cdNV_RolPA_C vs. S80_B	-0.3333	-1.097 to 0.4305	No	ns	0.9976
cdNV_RolPA_C vs. S80_C	-0.15	-0.9138 to 0.6138	No	ns	>0.9999
cdNV_TGF_C vs. cdNV_DMEM_C	-0.1367	-0.9005 to 0.6271	No	ns	>0.9999
cdNV_TGF_C vs. sn_S80_A	0.01	-0.7538 to 0.7738	No	ns	>0.9999
cdNV_TGF_C vs. sn_S80_B	0.09667	-0.6671 to 0.8605	No	ns	>0.9999
cdNV_TGF_C vs. sn_S80_C	0.04	-0.7238 to 0.8038	No	ns	>0.9999
cdNV_TGF_C vs. sn_RolPA_A	0.05	-0.7138 to 0.8138	No	ns	>0.9999

cdNV_TGF_C vs. sn_RolPA_B	0.03	-0.7338 to 0.7938	No	ns	>0.9999
cdNV_TGF_C vs. sn_RolPA_C	0.1367	-0.6271 to 0.9005	No	ns	>0.9999
cdNV_TGF_C vs. sn_TGF_A	0.1267	-0.6371 to 0.8905	No	ns	>0.9999
cdNV_TGF_C vs. sn_TGF_B	0.3217	-0.5323 to 1.176	No	ns	0.9998
cdNV_TGF_C vs. sn_TGF_C	0.1933	-0.5705 to 0.9571	No	ns	>0.9999
cdNV_TGF_C vs. sn_DMEM_A	0.05333	-0.7105 to 0.8171	No	ns	>0.9999
cdNV_TGF_C vs. sn_DMEM_B	0.08333	-0.6805 to 0.8471	No	ns	>0.9999
cdNV_TGF_C vs. sn_DMEM_C	-0.05	-0.8138 to 0.7138	No	ns	>0.9999
cdNV_TGF_C vs. S80_A	-0.3833	-1.147 to 0.3805	No	ns	0.9819
cdNV_TGF_C vs. S80_B	-0.2767	-1.04 to 0.4871	No	ns	>0.9999
cdNV_TGF_C vs. S80_C	-0.09333	-0.8571 to 0.6705	No	ns	>0.9999
cdNV_DMEM_C vs. sn_S80_A	0.1467	-0.6171 to 0.9105	No	ns	>0.9999
cdNV_DMEM_C vs. sn_S80_B	0.2333	-0.5305 to 0.9971	No	ns	>0.9999
cdNV_DMEM_C vs. sn_S80_C	0.1767	-0.5871 to 0.9405	No	ns	>0.9999
cdNV_DMEM_C vs. sn_RolPA_A	0.1867	-0.5771 to 0.9505	No	ns	>0.9999
cdNV_DMEM_C vs. sn_RolPA_B	0.1667	-0.5971 to 0.9305	No	ns	>0.9999
cdNV_DMEM_C vs. sn_RolPA_C	0.2733	-0.4905 to 1.037	No	ns	>0.9999
cdNV_DMEM_C vs. sn_TGF_A	0.2633	-0.5005 to 1.027	No	ns	>0.9999
cdNV_DMEM_C vs. sn_TGF_B	0.4583	-0.3956 to 1.312	No	ns	0.9592
cdNV_DMEM_C vs. sn_TGF_C	0.33	-0.4338 to 1.094	No	ns	0.998
cdNV_DMEM_C vs. sn_DMEM_A	0.19	-0.5738 to 0.9538	No	ns	>0.9999
cdNV_DMEM_C vs. sn_DMEM_B	0.22	-0.5438 to 0.9838	No	ns	>0.9999
cdNV_DMEM_C vs. sn_DMEM_C	0.08667	-0.6771 to 0.8505	No	ns	>0.9999
cdNV_DMEM_C vs. S80_A	-0.2467	-1.01 to 0.5171	No	ns	>0.9999
cdNV_DMEM_C vs. S80_B	-0.14	-0.9038 to 0.6238	No	ns	>0.9999
cdNV_DMEM_C vs. S80_C	0.04333	-0.7205 to 0.8071	No	ns	>0.9999
sn_S80_A vs. sn_S80_B	0.08667	-0.6771 to 0.8505	No	ns	>0.9999
sn_S80_A vs. sn_S80_C	0.03	-0.7338 to 0.7938	No	ns	>0.9999
sn_S80_A vs. sn_RolPA_A	0.04	-0.7238 to 0.8038	No	ns	>0.9999
sn_S80_A vs. sn_RolPA_B	0.02	-0.7438 to 0.7838	No	ns	>0.9999
sn_S80_A vs. sn_RolPA_C	0.1267	-0.6371 to 0.8905	No	ns	>0.9999
sn_S80_A vs. sn_TGF_A	0.1167	-0.6471 to 0.8805	No	ns	>0.9999
sn_S80_A vs. sn_TGF_B	0.3117	-0.5423 to 1.166	No	ns	>0.9999
sn_S80_A vs. sn_TGF_C	0.1833	-0.5805 to 0.9471	No	ns	>0.9999
sn_S80_A vs. sn_DMEM_A	0.04333	-0.7205 to 0.8071	No	ns	>0.9999
sn_S80_A vs. sn_DMEM_B	0.07333	-0.6905 to 0.8371	No	ns	>0.9999
sn_S80_A vs. sn_DMEM_C	-0.06	-0.8238 to 0.7038	No	ns	>0.9999
sn_S80_A vs. S80_A	-0.3933	-1.157 to 0.3705	No	ns	0.975
sn_S80_A vs. S80_B	-0.2867	-1.05 to 0.4771	No	ns	0.9998

sn_S80_A vs. S80_C	-0.1033	-0.8671 to 0.6605	No	ns	>0.9999
sn_S80_B vs. sn_S80_C	-0.05667	-0.8205 to 0.7071	No	ns	>0.9999
sn_S80_B vs. sn_RolPA_A	-0.04667	-0.8105 to 0.7171	No	ns	>0.9999
sn_S80_B vs. sn_RolPA_B	-0.06667	-0.8305 to 0.6971	No	ns	>0.9999
sn_S80_B vs. sn_RolPA_C	0.04	-0.7238 to 0.8038	No	ns	>0.9999
sn_S80_B vs. sn_TGF_A	0.03	-0.7338 to 0.7938	No	ns	>0.9999
sn_S80_B vs. sn_TGF_B	0.225	-0.629 to 1.079	No	ns	>0.9999
sn_S80_B vs. sn_TGF_C	0.09667	-0.6671 to 0.8605	No	ns	>0.9999
sn_S80_B vs. sn_DMEM_A	-0.04333	-0.8071 to 0.7205	No	ns	>0.9999
sn_S80_B vs. sn_DMEM_B	-0.01333	-0.7771 to 0.7505	No	ns	>0.9999
sn_S80_B vs. sn_DMEM_C	-0.1467	-0.9105 to 0.6171	No	ns	>0.9999
sn_S80_B vs. S80_A	-0.48	-1.244 to 0.2838	No	ns	0.815
sn_S80_B vs. S80_B	-0.3733	-1.137 to 0.3905	No	ns	0.9873
sn_S80_B vs. S80_C	-0.19	-0.9538 to 0.5738	No	ns	>0.9999
sn_S80_C vs. sn_RolPA_A	0.01	-0.7538 to 0.7738	No	ns	>0.9999
sn_S80_C vs. sn_RolPA_B	-0.01	-0.7738 to 0.7538	No	ns	>0.9999
sn_S80_C vs. sn_RolPA_C	0.09667	-0.6671 to 0.8605	No	ns	>0.9999
sn_S80_C vs. sn_TGF_A	0.08667	-0.6771 to 0.8505	No	ns	>0.9999
sn_S80_C vs. sn_TGF_B	0.2817	-0.5723 to 1.136	No	ns	>0.9999
sn_S80_C vs. sn_TGF_C	0.1533	-0.6105 to 0.9171	No	ns	>0.9999
sn_S80_C vs. sn_DMEM_A	0.01333	-0.7505 to 0.7771	No	ns	>0.9999
sn_S80_C vs. sn_DMEM_B	0.04333	-0.7205 to 0.8071	No	ns	>0.9999
sn_S80_C vs. sn_DMEM_C	-0.09	-0.8538 to 0.6738	No	ns	>0.9999
sn_S80_C vs. S80_A	-0.4233	-1.187 to 0.3405	No	ns	0.9418
sn_S80_C vs. S80_B	-0.3167	-1.08 to 0.4471	No	ns	0.999
sn_S80_C vs. S80_C	-0.1333	-0.8971 to 0.6305	No	ns	>0.9999
sn_RolPA_A vs. sn_RolPA_B	-0.02	-0.7838 to 0.7438	No	ns	>0.9999
sn_RolPA_A vs. sn_RolPA_C	0.08667	-0.6771 to 0.8505	No	ns	>0.9999
sn_RolPA_A vs. sn_TGF_A	0.07667	-0.6871 to 0.8405	No	ns	>0.9999
sn_RolPA_A vs. sn_TGF_B	0.2717	-0.5823 to 1.126	No	ns	>0.9999
sn_RolPA_A vs. sn_TGF_C	0.1433	-0.6205 to 0.9071	No	ns	>0.9999
sn_RolPA_A vs. sn_DMEM_A	0.003333	-0.7605 to 0.7671	No	ns	>0.9999
sn_RolPA_A vs. sn_DMEM_B	0.03333	-0.7305 to 0.7971	No	ns	>0.9999
sn_RolPA_A vs. sn_DMEM_C	-0.1	-0.8638 to 0.6638	No	ns	>0.9999
sn_RolPA_A vs. S80_A	-0.4333	-1.197 to 0.3305	No	ns	0.9259
sn_RolPA_A vs. S80_B	-0.3267	-1.09 to 0.4371	No	ns	0.9983
sn_RolPA_A vs. S80_C	-0.1433	-0.9071 to 0.6205	No	ns	>0.9999
sn_RolPA_B vs. sn_RolPA_C	0.1067	-0.6571 to 0.8705	No	ns	>0.9999
sn_RolPA_B vs. sn_TGF_A	0.09667	-0.6671 to 0.8605	No	ns	>0.9999

sn_RolPA_B vs. sn_TGF_B	0.2917	-0.5623 to 1.146	No	ns	>0.9999
sn_RolPA_B vs. sn_TGF_C	0.1633	-0.6005 to 0.9271	No	ns	>0.9999
sn_RolPA_B vs. sn_DMEM_A	0.02333	-0.7405 to 0.7871	No	ns	>0.9999
sn_RolPA_B vs. sn_DMEM_B	0.05333	-0.7105 to 0.8171	No	ns	>0.9999
sn_RolPA_B vs. sn_DMEM_C	-0.08	-0.8438 to 0.6838	No	ns	>0.9999
sn_RolPA_B vs. S80_A	-0.4133	-1.177 to 0.3505	No	ns	0.9552
sn_RolPA_B vs. S80_B	-0.3067	-1.07 to 0.4571	No	ns	0.9994
sn_RolPA_B vs. S80_C	-0.1233	-0.8871 to 0.6405	No	ns	>0.9999
sn_RolPA_C vs. sn_TGF_A	-0.01	-0.7738 to 0.7538	No	ns	>0.9999
sn_RolPA_C vs. sn_TGF_B	0.185	-0.669 to 1.039	No	ns	>0.9999
sn_RolPA_C vs. sn_TGF_C	0.05667	-0.7071 to 0.8205	No	ns	>0.9999
sn_RolPA_C vs. sn_DMEM_A	-0.08333	-0.8471 to 0.6805	No	ns	>0.9999
sn_RolPA_C vs. sn_DMEM_B	-0.05333	-0.8171 to 0.7105	No	ns	>0.9999
sn_RolPA_C vs. sn_DMEM_C	-0.1867	-0.9505 to 0.5771	No	ns	>0.9999
sn_RolPA_C vs. S80_A	-0.52	-1.284 to 0.2438	No	ns	0.6785
sn_RolPA_C vs. S80_B	-0.4133	-1.177 to 0.3505	No	ns	0.9552
sn_RolPA_C vs. S80_C	-0.23	-0.9938 to 0.5338	No	ns	>0.9999
sn_TGF_A vs. sn_TGF_B	0.195	-0.659 to 1.049	No	ns	>0.9999
sn_TGF_A vs. sn_TGF_C	0.06667	-0.6971 to 0.8305	No	ns	>0.9999
sn_TGF_A vs. sn_DMEM_A	-0.07333	-0.8371 to 0.6905	No	ns	>0.9999
sn_TGF_A vs. sn_DMEM_B	-0.04333	-0.8071 to 0.7205	No	ns	>0.9999
sn_TGF_A vs. sn_DMEM_C	-0.1767	-0.9405 to 0.5871	No	ns	>0.9999
sn_TGF_A vs. S80_A	-0.51	-1.274 to 0.2538	No	ns	0.7153
sn_TGF_A vs. S80_B	-0.4033	-1.167 to 0.3605	No	ns	0.9662
sn_TGF_A vs. S80_C	-0.22	-0.9838 to 0.5438	No	ns	>0.9999
sn_TGF_B vs. sn_TGF_C	-0.1283	-0.9823 to 0.7256	No	ns	>0.9999
sn_TGF_B vs. sn_DMEM_A	-0.2683	-1.122 to 0.5856	No	ns	>0.9999
sn_TGF_B vs. sn_DMEM_B	-0.2383	-1.092 to 0.6156	No	ns	>0.9999
sn_TGF_B vs. sn_DMEM_C	-0.3717	-1.226 to 0.4823	No	ns	0.9977
sn_TGF_B vs. S80_A	-0.705	-1.559 to 0.149	No	ns	0.2781
sn_TGF_B vs. S80_B	-0.5983	-1.452 to 0.2556	No	ns	0.6207
sn_TGF_B vs. S80_C	-0.415	-1.269 to 0.439	No	ns	0.9882
sn_TGF_C vs. sn_DMEM_A	-0.14	-0.9038 to 0.6238	No	ns	>0.9999
sn_TGF_C vs. sn_DMEM_B	-0.11	-0.8738 to 0.6538	No	ns	>0.9999
sn_TGF_C vs. sn_DMEM_C	-0.2433	-1.007 to 0.5205	No	ns	>0.9999
sn_TGF_C vs. S80_A	-0.5767	-1.34 to 0.1871	No	ns	0.4604
sn_TGF_C vs. S80_B	-0.47	-1.234 to 0.2938	No	ns	0.8438
sn_TGF_C vs. S80_C	-0.2867	-1.05 to 0.4771	No	ns	0.9998
sn_DMEM_A vs. sn_DMEM_B	0.03	-0.7338 to 0.7938	No	ns	>0.9999
sn_DMEM_A vs. sn_DMEM_C	-0.1033	-0.8671 to 0.6605	No	ns	>0.9999
sn_DMEM_A vs. S80_A	-0.4367	-1.2 to 0.3271	No	ns	0.9199

sn_DMEM_A vs. S80_B	-0.33	-1.094 to 0.4338	No	ns	0.998
sn_DMEM_A vs. S80_C	-0.1467	-0.9105 to 0.6171	No	ns	>0.9999
sn_DMEM_B vs. sn_DMEM_C	-0.1333	-0.8971 to 0.6305	No	ns	>0.9999
sn_DMEM_B vs. S80_A	-0.4667	-1.23 to 0.2971	No	ns	0.8528
sn_DMEM_B vs. S80_B	-0.36	-1.124 to 0.4038	No	ns	0.9923
sn_DMEM_B vs. S80_C	-0.1767	-0.9405 to 0.5871	No	ns	>0.9999
sn_DMEM_C vs. S80_A	-0.3333	-1.097 to 0.4305	No	ns	0.9976
sn_DMEM_C vs. S80_B	-0.2267	-0.9905 to 0.5371	No	ns	>0.9999
sn_DMEM_C vs. S80_C	-0.04333	-0.8071 to 0.7205	No	ns	>0.9999
S80_A vs. S80_B	0.1067	-0.6571 to 0.8705	No	ns	>0.9999
S80_A vs. S80_C	0.29	-0.4738 to 1.054	No	ns	0.9998
S80_B vs. S80_C	0.1833	-0.5805 to 0.9471	No	ns	>0.9999

Table S10. Statistical analysis.
Results of one-way ANOVA test with a post-hoc Tukey test of the zeta potential in Figure 6a.

**Number of families**	1				
**Number of comparisons per family**	3				
**Alpha**	0.05				
**Tukey's multiple comparisons test**	**Mean Diff.**	**95.00% CI of diff.**	**Significant?**	**Summary**	**Adjusted P Value**
cdNV-L vs. PL	-6.96	-14.58 to 0.6634	No	ns	0.073
cdNV-L vs. PL-200	-7.815	-15.44 to -0.1916	Yes	*	0.0448
PL vs. PL-200	-0.855	-8.478 to 6.768	No	ns	0.9477

Table S11. Statistical analysis.
Results of one-way ANOVA test with a post-hoc Tukey test of COL1A1 mRNA transcription in Figure 6e.

**Number of families**	1				
**Number of comparisons per family**	6				
**Alpha**	0.05				
**Tukey's multiple comparisons test**	**Mean Diff.**	**95.00% CI of diff.**	**Significant?**	**Summary**	**Adjusted P Value**
DMEM vs. PL_200_A	0.1788	0.007695 to 0.3499	Yes	*	0.0408
DMEM vs. sn_200	0.03878	-0.1323 to 0.2099	No	ns	0.8841
DMEM vs. cdNV_A	0.2521	0.08103 to 0.4232	Yes	**	0.0065
PL_200_A vs. sn_200	-0.14	-0.3111 to 0.03109	No	ns	0.1136
PL_200_A vs. cdNV_A	0.07333	-0.09775 to 0.2444	No	ns	0.5477
sn_200 vs. cdNV_A	0.2133	0.04225 to 0.3844	Yes	*	0.0168

Table S12. *Statistical analysis.*
Results of one-way ANOVA test with a post-hoc Tukey test of SPARC mRNA transcription in Figure 6e.

**Number of families**	1				
**Number of comparisons per family**	6				
**Alpha**	0.05				
**Tukey's multiple comparisons test**	**Mean Diff.**	**95.00% CI of diff.**	**Significant?**	**Summary**	**Adjusted P Value**
DMEM vs. PL_200_A	0.4136	0.2356 to 0.5916	Yes	***	0.0003
DMEM vs. sn_200	0.4076	0.2296 to 0.5856	Yes	***	0.0004
DMEM vs. cdNV_A	0.2345	0.05655 to 0.4125	Yes	*	0.0124
PL_200_A vs. sn_200	-0.005999	-0.184 to 0.172	No	ns	0.9995
PL_200_A vs. cdNV_A	-0.1791	-0.357 to -0.001139	Yes	*	0.0486
sn_200 vs. cdNV_A	-0.1731	-0.351 to 0.00486	No	ns	0.0566

Table S13. Statistical analysis.
Results of one-way ANOVA test with a post-hoc Tukey test of cellular particle uptake in naïve (n) and perpetuated (p) LX-2 cells shown in Figure 7b.

Number of families	1				
Number of comparisons per family	28				
Alpha	0.05				
Tukey's multiple comparisons test	Mean Diff.	95.00% CI of diff.	Significant ?	Summar y	Adjusted P Value
p_cdNV-L vs. p_cdNV	-0.1303	-0.228 to -0.03266	Yes	**	0.0051
p_cdNV-L vs. p_PL_200	-0.01233	-0.1168 to 0.09209	No	ns	0.9999
p_cdNV-L vs. p_PL	0.07333	-0.03109 to 0.1778	No	ns	0.2955
p_cdNV-L vs. n_cdNV-L	0.08067	-0.02376 to 0.1851	No	ns	0.2031
p_cdNV-L vs. n_cdNV	-0.035	-0.1394 to 0.06942	No	ns	0.9352
p_cdNV-L vs. n_PL_200	0.069	-0.03542 to 0.1734	No	ns	0.3621
p_cdNV-L vs. n_PL	0.08133	-0.02309 to 0.1858	No	ns	0.196
p_cdNV vs. p_PL_200	0.118	0.02032 to 0.2157	Yes	*	0.0122
p_cdNV vs. p_PL	0.2037	0.106 to 0.3013	Yes	****	<0.0001
p_cdNV vs. n_cdNV-L	0.211	0.1133 to 0.3087	Yes	****	<0.0001
p_cdNV vs. n_cdNV	0.09533	-0.002345 to 0.193	No	ns	0.0585
p_cdNV vs. n_PL_200	0.1993	0.1017 to 0.297	Yes	****	<0.0001
p_cdNV vs. n_PL	0.2117	0.114 to 0.3093	Yes	****	<0.0001
p_PL_200 vs. p_PL	0.08567	-0.01876 to 0.1901	No	ns	0.1543
p_PL_200 vs. n_cdNV-L	0.093	-0.01142 to 0.1974	No	ns	0.1009
p_PL_200 vs. n_cdNV	-0.02267	-0.1271 to 0.08176	No	ns	0.9939
p_PL_200 vs. n_PL_200	0.08133	-0.02309 to 0.1858	No	ns	0.196
p_PL_200 vs. n_PL	0.09367	-0.01076 to 0.1981	No	ns	0.097
p_PL vs. n_cdNV-L	0.007333	-0.09709 to 0.1118	No	ns	>0.9999
p_PL vs. n_cdNV	-0.1083	-0.2128 to -0.003911	Yes	*	0.039
p_PL vs. n_PL_200	-0.004333	-0.1088 to 0.1001	No	ns	>0.9999
p_PL vs. n_PL	0.008	-0.09642 to 0.1124	No	ns	>0.9999
n_cdNV-L vs. n_cdNV	-0.1157	-0.2201 to -0.01124	Yes	*	0.0243
n_cdNV-L vs. n_PL_200	-0.01167	-0.1161 to 0.09276	No	ns	>0.9999
n_cdNV-L vs. n_PL	0.0006667	-0.1038 to 0.1051	No	ns	>0.9999
n_cdNV vs. n_PL_200	0.104	-0.0004221 to 0.2084	No	ns	0.0513
n_cdNV vs. n_PL	0.1163	0.01191 to 0.2208	Yes	*	0.0232
n_PL_200 vs. n_PL	0.01233	-0.09209 to 0.1168	No	ns	0.9999
